# Biosynthesis-Guided Discovery and Engineering of α-Pyrone Natural Products from Type I Polyketide Synthases

**DOI:** 10.1021/acschembio.3c00081

**Published:** 2023-04-19

**Authors:** Dongqi Yi, Vinayak Agarwal

**Affiliations:** † School of Chemistry and Biochemistry, Georgia Institute of Technology, Atlanta, Georgia 30332, United States; ‡ School of Biological Sciences, Georgia Institute of Technology, Atlanta, Georgia 30332, United States

## Abstract

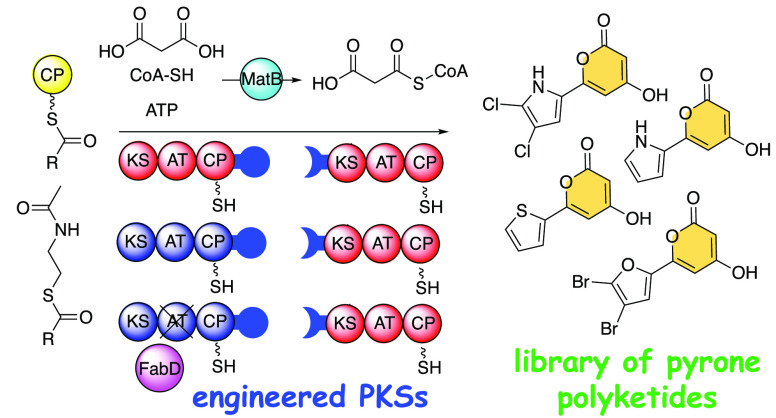

Natural products containing the α-pyrone moiety are produced by polyketide synthases (PKSs) in bacteria, fungi, and plants. The conserved biosynthetic logic for the production of the α-pyrone moiety involves the cyclization of a triketide intermediate which also off-loads the polyketide from the activating thioester. In this study, we show that truncating a tetraketide natural product producing PKS assembly line allows for a thioesterase-independent off-loading of an α-pyrone polyketide natural product, one which we find to be natively present in the extracts of the bacterium that otherwise furnishes the tetraketide natural product. By engineering the truncated PKS *in vitro*, we demonstrate that a ketosynthase (KS) domain with relaxed substrate selectivity when coupled with in trans acylation of polyketide extender units can expand the chemical space of α-pyrone polyketide natural products. Findings from this study point toward heterologous intermolecular protein–protein interactions being detrimental to the efficiency of engineered PKS assembly lines.

Polyketide natural products containing the α-pyrone moiety are biosynthesized by modular type I polyketide synthases (PKSs), iterative type II and type III PKSs, and fungal nonreducing PKSs (NRPKSs).^1−5^ Examples of natural products furnished by the above-mentioned three PKS types include venemycin (**1**, Figure 1A), triacetic acid lactone (**2**), and pyrophen (**3**), respectively. In each case, progressing from a carboxylic acid thioesterified either to coenzyme A (CoA-SH) or to the phosphopantetheine thiol of a carrier protein (CP), two polyketide extension reactions furnish a triketide intermediate that is then off-loaded via intramolecular annulation to furnish the pyrone natural product (Figure 1B). Reductive tailoring of the triketone prior to off-loading produces lactones rather than pyrones.

**Figure 1:**
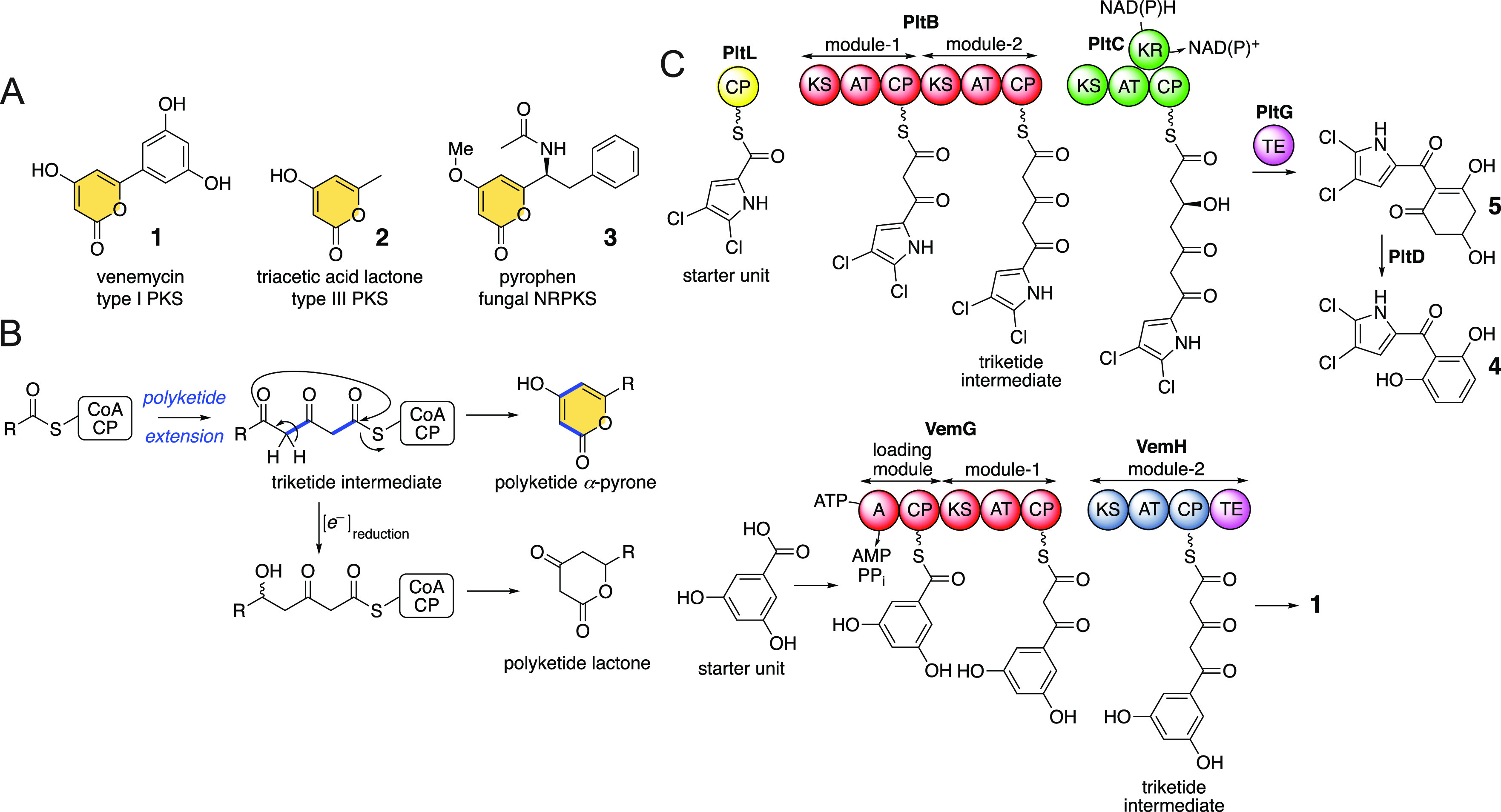
(A) Examples of polyketide natural products containing the α-pyrone moiety (shaded). (B) Formation of the α-pyrones involves triketide cyclization and off-loading; reduction of the triketide leads to lactone production. (C) The Plt and Vem assembly lines.

We recently described the *in vitro* reconstitution of the pyoluteorin (**4**, Figure 1C) biosynthetic pathway.^6,7^ Here, three modules of the type I PKSs PltB and PltC afford a tetraketide that is reduced by the PltC ketoreductase (KR) domain. Off-loading by the standalone thioesterase (TE) PltG furnished **5** that was dehydrated and aromatized to **4**. Curiously, organization of the first two modules of the Plt PKSs is similar to the Vem PKSs that produce **1** (Figure 1C).^1,8^ Despite the differences in the starter units (4,5-dichloropyrrole carboxylic acid for **4**; 3,5-dihydroxybenzoic acid for **1**) and the mechanism of the delivery of these starter units to the initiating ketosynthase (KS) domains of the respective PKSs, no reductive tailoring of the triketide intermediate occurs in either assembly line. For the Plt assembly line, the triketide intermediate is further extended to a tetraketide followed by a Dieckmann cyclization likely catalyzed by the PltG TE to afford **5**, while for the Vem assembly line, the terminal TE domain embedded in the VemH PKS off-loads the triketide via an intramolecular esterification to furnish **1**.

Motivated by the similarities in module organization of Plt and Vem PKSs, we asked if the Plt assembly line could be engineered to deliver α-pyrone polyketide products akin to the Vem pathway. For the Vem pathway, selectivity for the starter unit is generated by the adenylation (A) domain of the VemG loading module wherein this A-domain adenylates 3,5-dihydroxybenzoic acid and thioesterifies it to the phosphopantetheine arm of the VemG loading module CP (Figure 1C).^1^ For the Plt pathway, the PltB module-1 KS screens and selects for the starter unit that is delivered by the upstream pyrrole maturation and halogenating enzymes. In light of the differential mechanism for starter unit delivery to the PltB PKS as compared to the VemG PKS, we also explored if PKS engineering could expand the chemical space of the delivered α-pyrone products.

With the observation that both the Vem and Plt PKSs produce a nonreduced triketide intermediate, we explored, *in vitro*, whether the first two modules of the Plt PKS could mimic the activity of the Vem PKS (Figure 1C). The two Plt modules were separated by appending docking domains from the 6-deoxyerythronolide B synthase (DEBS) assembly line to the C-terminus of PltB module-1 and to the N-terminus of PltB module-2 and purified along with the TE PltG (Figure 2A).^6^ The starter unit, 4,5-dichloropyrrole carboxylic acid thioesterified to the CP PltL, 4,5-dichloropyrrolyl-*S*-PltL, was chemoenzymatically synthesized (Figure S1).^9^ The enzyme MatB was employed to generate malonyl-CoA *in situ* using malonate, CoA-SH, and ATP;^10^ malonyl-CoA is used as the substrate by the acyltransferase (AT) domains of the PltB PKS modules. Upon incubation of the assay components, we observed the accumulation of a dichlorinated product (Figures 2B and S2–S4). The production of **4** or **5** was not detected using liquid chromatography/mass spectrometry (LC/MS).

**Figure 2:**
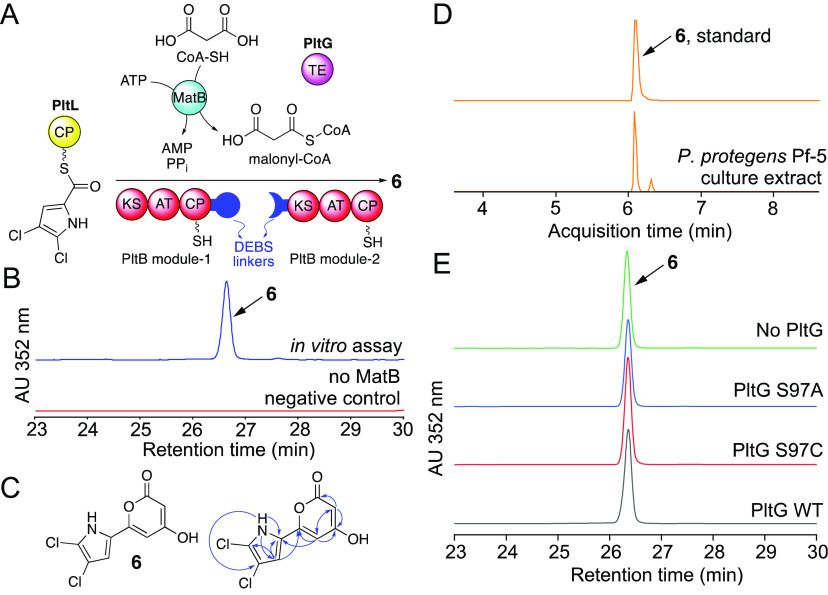
(A) Assay design for production of **6**. (B) UV-absorbance chromatograms demonstrating the production of **6**. A negative control reaction in which MatB was omitted did not produce **6**. (C) Structure of **6** with key HMBC correlations shown as blue arrows. (D) Extracted ion chromatograms demonstrating the presence of **6** in *P. protegens* Pf-5 culture extracts, as compared to a purified standard of **6**. (E) UV-absorbance chromatograms for end point assays demonstrating similar abundance of **6** in reactions carried out as illustrated in panel A, with PltG variants S97A and S97C and when PltG was omitted from the reaction.

The abundance of the conceivable α-pyrone produced in this assay was limited by the amount of 4,5-dichloropyrrolyl-*S*-PltL substrate that we could provide in an *in vitro* assay. To circumvent this challenge, we coexpressed genes encoding the PltB PKS modules in *E. coli* together with genes encoding MatB and the phosphopantetheinyl transferase Sfp.^11^ The cell lysate of this *E. coli* strain was then directly used as a catalyst in an *in vitro* assay wherein we replaced 4,5-dichloropyrrolyl-*S*-PltL with 4,5-dichloropyrrolyl-*S*,*N*-acetylcysteamine (4,5-dichloropyrrolyl-SNAC, **8a**, Scheme S1, Figure S5–S6). These modifications allowed for preparative isolation and structural characterization of the product as the α-pyrone **6** (Figures 2C and S7–S11, Table S1). These data demonstrate that truncating the Plt assembly line to two modules is sufficient to change its output from the dihydrophloroglucinol **5** to an α-pyrone **6**.

Components added to the assay illustrated in Figure 2A are all present *in situ* in the *Pseudomonas protegens* Pf-5 bacterium that produces **4**. However, no pyrone natural products have been reported from *P. protegens* Pf-5. Using LC/MS, we could indeed detect the presence of **6** in the extracts of *P. protegens* Pf-5 (Figure 2D). This result establishes the *in vitro* enzymatically synthesized **6** as a physiologically produced natural product that had evaded prior detection and characterization.

The production of triketide pyrones by the Vem assembly line involves a TE domain that is embedded within the VemH PKS (Figure 1C).^1,8^ The Plt assembly line presents a different scenario; here the standalone thioesterase PltG is involved in the production of **5**. In complete contrast, no embedded or standalone TEs are partnered with either the fungal NRPKSs that produce **3** or the bacterial type II PKS that produces α-pyrone polyketides enterocins and wailupemycins.^3,4^ In light of these differences, we sought to evaluate the role of PltG in the production of **6**. Variants of PltG were prepared in which the serine residue in the PltG active site was replaced with alanine (PltG S97A) and cysteine (PltG S97C). While the S97A mutation would abolish the thioesterase activity, the S97C mutation could preserve or even enhance^12^ the thioesterase activity of PltG. The PltG variants were trialed in the above-mentioned assay with the 4,5-dichloropyrrolyl-*S*-PltL initiator substrate and purified PltB PKS modules. Chromatographic detection of **6** at similar abundance in all four assays demonstrates that the formation of the α-pyrone product did not require the TE participation (Figure 2E). This observation allows us to posit that off-loading of a triketide as an α-pyrone is noncatalytic, likely driven by the thermodynamic stability of the aromatic product.^3,4^ This then calls into question the role of the terminal thioesterase domain embedded in the VemH PKS module in the production of **1**.

We have reported previously that the substrate selectivity of the PltB module-1 KS domain constrains the diversity of starter units that can progress along Plt PKS assembly line.^13^ In the experimental setup illustrated in Figure 3A, which did not include the PltG TE, we provided the dichloropyrrolyl- and the pyrrolyl-*S*-PltL initiator substrates to the PltB PKS modules. Progressing from the physiological substrate dichloropyrrolyl-*S*-PltL, we observed a time-dependent accumulation of **6**. In contrast, starting from pyrrolyl-*S*-PltL, the level of production of the deschloro derivative of **6**, molecule **7**, was much lower (Figures 3B and S12–S14).

**Figure 3:**
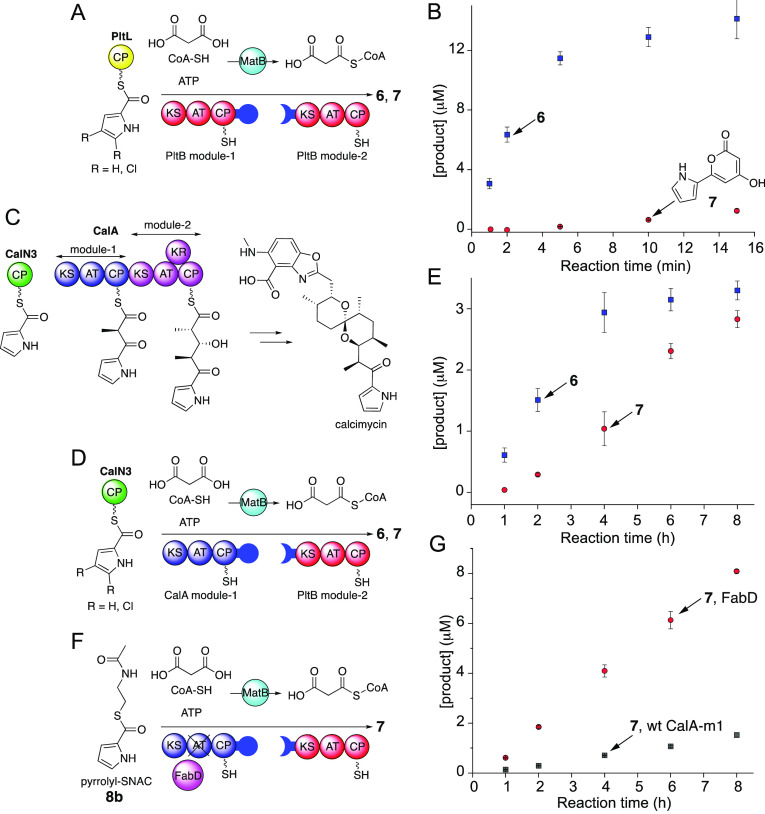
(A) Assay design to test substrate preference of the Plt PKS assembly line. The dichloropyrrolyl- and the pyrrolyl-*S*-PltL substrates lead to **6** and **7**, respectively. (B) Time-dependent accumulation of **6** and **7** produced in the assay illustrated in panel A. (C) The Cal PKS; only the first two modules of the Cal PKS are shown. The pyrrole carboxylic acid starter unit is delivered via the CP CalN3. (D) Assay design in which the PltB module-1 is replaced with the CalA module-1. (E) Time-dependent accumulation of **6** and **7** produced in the assay illustrated in panel D. (F) Assay design in which the CalA module-1 AT domain is inactivated and the *E. coli* FabD enzyme is provided to acylate the CalA module-1 CP in trans. (G) Time-dependent accumulation of **7** produced in the assay illustrated in panel F and with wild-type CalA module-1 with a functional AT domain.

In addition to the substrate selectivity of the PltB module-1 KS domain, in our previous study, we had characterized the substrate promiscuity of the CalA module-1 KS domain.^13^ The Cal PKS assembly line uses the pyrrolyl-*S*-CalN3 initiator substrate to furnish the pyrrolic antibiotic calcimycin (Figure 3C).^14^ Unlike the Plt PKSs which use malonyl extender units, the Cal PKS module-1 and module-2, which are both present within the CalA polypeptide, use methylmalonyl extender units. The CalA module-1 KS domain was found to accept both pyrrolyl- (its physiological substrate) and the dichloropyrrolyl starter units.^13^ In light of these observations, we asked if the CalA module-1 KS domain could be used to circumvent the substrate selectivity of the PltB module-1 KS domain. With this motivation, we swapped the PltB module-1 with the CalA module-1 (Figure 3D). To maintain physiological intermolecular protein–protein contacts, in this assay, the initiator substrates were delivered by the Cal CP, CalN3. Upon incubation of reaction components, we observed a higher level of production of **7** relative to **6** (Figure 3E), as compared to the pyrone production assay with the PltB module-1 (Figure 3B). Thus, the relaxed substrate selectivity of the CalA module-1 KS domain indeed allows for expansion of the α-pyrone product chemical space.

While the abundance of **7** relative to **6** was enhanced by swapping out the PltB module-1 and replacing it with CalA module-1, the product abundances and rates of product formation were diminished (Figure 3B,E). We have previously demonstrated that the delivery of the dichloropyrrolyl molecular cargo to the initiating PltB module-1 and CalA module-1 KSs from their cognate CPs (PltL and CalN3, respectively) was equally efficient and was thus not a likely contributing factor to the reduction in abundance and rate of product formation, at the very least for **6**.^13^ Progressing along the PKS assembly lines, there could then be three possible reasons for the decrease in abundance and rate of product formation. First, in the assay set up illustrated in Figure 3D, despite the presence of the DEBS linker domains, the intermolecular protein–protein interactions between the CalA module-1 and the PltB module-2 are non-native (Figure 3D). This mismatch could compromise the efficiency of the transthioesterification of the diketide intermediate from CalA module-1 CP to the PltB module-2 KS.^15,16^ Second, the downstream PltB module-2 KS domain could gatekeep against the extension of a nonphysiological diketide intermediate furnished by the upstream PKS module-1.^17^ However, the potential gatekeeping activity of PltB module-2 KS is unlikely to affect biosynthesis of **6**, which we *also* observed to be negatively impacted (rate and abundance of **6** in Figure 3B,E). Third, the physiological extender unit incorporated by the CalA module-1 AT domain is methylmalonyl-CoA and not malonyl-CoA. The forced incorporation of the non-native malonyl extender unit by the CalA module-1 AT could compromise the efficiency of the entire engineered bimodular PKS.

To test if the non-native extender unit incorporation by the CalA module-1 AT domain compromised the efficiency of the assay illustrated in Figure 3D, we inactivated the CalA module-1 AT domain by a serine to alanine mutation in the AT active site. To then acylate the CalA module-1 CP in trans, we added purified *E. coli* fatty acid malonyl-CoA:CP transacylase (MAT) FabD to the assay. The physiological substrate for FabD is malonyl-CoA, same as the PltB module-2 AT domain.^18^ In line with previous reports where trans-acting ATs have substituted for inactivated cis-ATs,^19^ the pyrrole carboxylic acid starter unit was thioesterified to SNAC (**8b**), rather than the CP CalN3 (Figures 3F and S15, Scheme S2). This change also potentially ameliorates substrate degradation due to the transacylating activity of FabD.

By observing an enhancement in the abundance of product **7** when FabD substituted in trans for the inactivated cis-acting CalA module-1 AT domain (Figure 3G), we could confirm that the mismatch in the extender units in the assay illustrated in Figure 3D was indeed one of the contributing factors that compromised rate and abundance of product formation. Engineering of the PKS AT domains is of intense contemporary interest and AT domains in collinear PKS modules have been replaced with cis-acting substrate promiscuous MATs to facilitate the delivery of engineered polyketides.^20−22^ Here, we have used a trans-acting MAT to substitute for an inactivated cis-acting AT domain to partially circumvent the regularly observed reduction in yields for engineered PKSs.

With an engineered system in hand to increase the α-pyrone product formation upon replacement of the CalA module-1 AT with FabD, we explored if we could expand the acylpyrone product profile. With this motivation, a panel of thioesterified SNAC starter units was developed (**8a**–**8s**; Figures 4A and S16–S44; Scheme S3–S19). In addition to derivatized pyrroles, the panel of SNAC-thioesters included pentacyclic thiophenes, furans, thiazole, oxazole, hexacyclic pyridines, a phenyl, and branched short chain alkanes. These starter units were provided to the *native* bimodular Plt PKSs and to the *engineered* CalA/PltB/FabD hybrid PKS assembly lines (Figure 4B). Product formation was monitored by mass spectrometry, including the characteristic MS/MS fragmentation of the acyl pyrones (Figures S45–S61). The product abundances were normalized to the abundance of **6**, produced starting from **8a**, as the dichloropyrrolyl starter unit was demonstrated to be competent substrate for both the PltB module-1 KS and the CalA module-1 KS (Figure 3).

**Figure 4:**
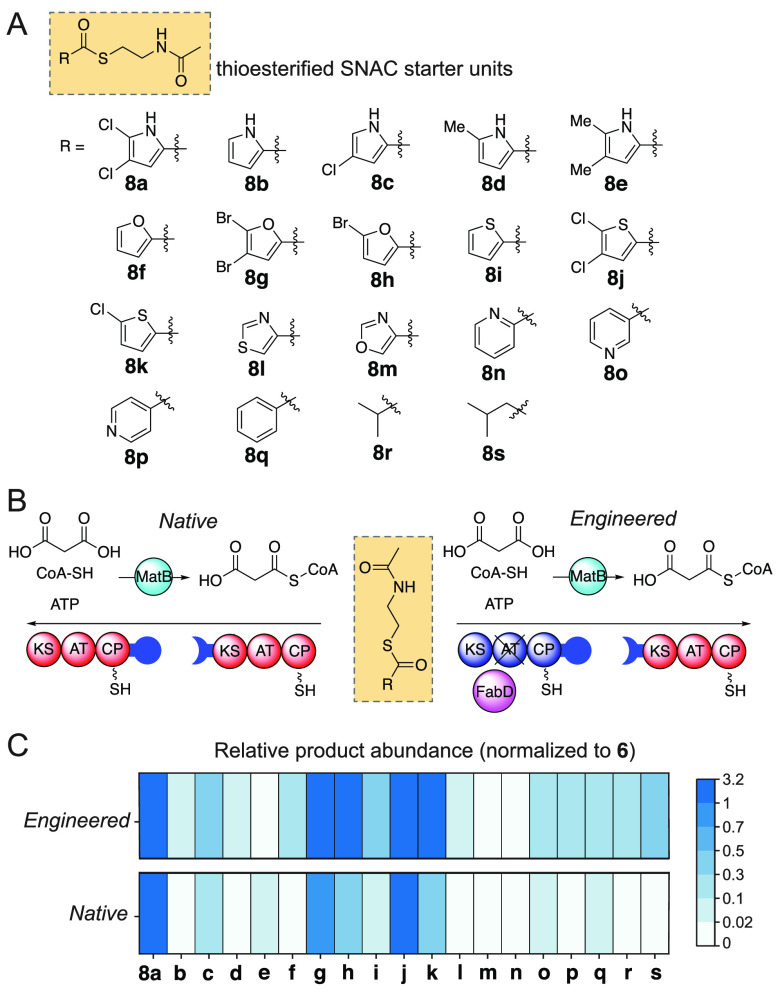
(A) Panel of synthetic thioesterified SNAC starter units prepared in this study. (B) The *Native* and *Engineered* PKS systems evaluated for α-pyrone polyketide product formation. (C) Heat map representing the abundance of α-pyrone products produced from the *Engineered* (top) and *Native* (bottom) PKS systems. The abundance of each product is normalized to **6**, which is produced starting from **8a**.

The normalized product abundances, illustrated as a heat map in Figure 4C, reveal trends for starter unit specificities (Table S2). In general, as compared to the native PKSs, the engineered PKSs/FabD indeed demonstrated an expanded product profile, with pyrone products detected in modest abundances starting from **8o**–**8s** which were poor substrates to the native PKS system. Interestingly, as for pyrroles, halogenated thiophenes and furans were competent substrates for both systems, with the engineered PKS system demonstrating greater relative product abundance as compared to the native PKS system for these substrates as well. The oxazole and thiazole were poor substrates in both cases.

Taken together, data described herein demonstrate that truncated type I PKSs can produce α-pyrones without the involvement of an off-loading thioesterase and that engineering efforts directed at circumventing the specificities of the KS and AT domains can expand the scope and yield of α-pyrone products. Several pyrone natural products possess validated pharmaceutical potential and also serve as precursors in synthetic schemes.^23^ The above-mentioned efforts thus could serve to generate a biocatalyst toolbox for the targeted delivery of substituted pyrones starting from simple thioesterified substrates with *in situ* enzymatic production of polyketide extender units, as has been achieved for other commodity chemicals.^24^ The promise of polyketide engineering, facile as it may seem given the vectorial nature of collinear assembly lines, is yet to be fully realized.^25,26^ Even for the simple bimodular extension necessary for the construction of α-pyrones, our study reveals that the selectivity of the KSs and the ATs and the heterologous intermodular interactions can constrain product yield. In addition to chemical delivery to satisfy medicinal, synthetic, or other commercial needs, the simple bimodular PKS systems described in this study could serve as testing systems to evaluate and evolve PKS engineering efforts in the future.

## Supporting Information Available

Experimental details for recombinant protein production, synthetic procedures, and analytical procedures for compound characterization, Figures S1–S61, Schemes S1–S19, Tables S1–S2, and Supplementary References (PDF)
