# Supplementary material for: Biosynthesis-Guided Discovery and Engineering of α-Pyrone Natural Products from Type I Polyketide Synthases
Source: ACS Chem Biol. 2023 Apr 19;18(5):1060–5. doi: 10.1021/acschembio.3c00081 (PMC10204065; doi:10.1021/acschembio.3c00081)
Supplement: Supplementary file 1 — cb3c00081_si_001.pdf [file cb3c00081_si_001.pdf]

SUPPLEMENTARY INFORMATION FOR:

Biosynthesis-guided discovery and engineering of α-pyrone natural products from type I polyketide synthases

Dongqi Yi[1] and Vinayak Agarwal[1,2,*]

[1]School of Chemistry and Biochemistry, Georgia Institute of Technology, Atlanta, GA 30332, USA

[2]School of Biological Sciences, Georgia Institute of Technology, Atlanta, GA 30332, USA

*Correspondence: vagarwal@gatech.edu; Ph: (+1)404-385-378

Supplementary Information document contains:

Supplementary Materials and Methods

Supplementary Schemes S1–S19

Supplementary Tables S1–S2

Supplementary Figures S1–S61

Supplementary References

SUPPLEMENTARY MATERIALS AND METHODS

General materials and instrumentation

All chemicals, solvents, and media components were obtained commercially from Sigma-Aldrich, Fisher Scientific, and VWR, and used without further purification. Phusion high-fidelity DNA polymerase and Gibson assembly Master Mix were purchased from New England Biolabs. PrimeSTAR DNA polymerase Master Mix was purchased from Takaro Bio. Synthetic DNA fragments were ordered from Twist Biosciences. Reactions were monitored by thin layer chromatography (TLC) carried out on Supelco silica gel (60 F_{254}) glass plates visualized by UV light. Silica gel (SiliaFlash GE60, 60–200 µm) was used for flash chromatography. Nuclear magnetic resonance (NMR) spectra were recorded on Bruker Avance III HD 400, 500, or 700 MHz instruments and calibrated using residual undeuterated solvent as the internal reference ($CDCl_3$ δ_H 7.26 and δ_C 77.16, MeOD δ_H 3.31 and δ_C 49.00, DMSO-d_6 δ_H 2.50 and δ_C 39.52). The splitting patterns were reported as s=singlet, d=doublet, t=triplet, q=quartet, m=multiplet, br=broad. Mass spectra were recorded on a Bruker Impact II high resolution time of flight (ToF) mass spectrometer with an electrospray ionization (ESI) source coupled to an Agilent 1290 ultra high-performance liquid chromatography system equipped with a diode array detector.

Vector construction

Standard molecular biology techniques were used to carry out plasmid construction. Amplification of target DNA fragments were carried out with either Phusion or PrimeSTAR high-fidelity DNA polymerases. Gibson assembly was used to subclone genes encoding for proteins of interest into the target vectors. The sequences of recombinant plasmids were confirmed by Sanger sequencing at Eton Biosciences.

The construction of PltB module1 with DEBS2 C-terminal docking domain (dd) sequences in pET28(+), PltB module2 with DEBS3 N-terminal docking domain sequences in pET28(+), PltG in pET24(+), Sfp in the first multiple cloning site (MCS) of pCDFDuet-1, MatB in pET28-MBP expression vectors has been described previously.[1-2] To construct the expression vector for CalA module1 fused with DEBS2 dd, *calA* module1 gene was amplified from previously synthesized pET28(+)-CalA module1 vector,[1] while the pET28(+) vector backbone with DEBS2dd sequences ligated was amplified using pET28(+)-PltB module1-DEBS2dd as the template. The amplified DNA fragment encoding for CalA module1 was then inserted into pET28(+) vector containing C-terminal DEBS2dd sequences using Gibson assembly. For the construction of FabD overexpression vector, the DNA fragment encoding *fabD* gene was first amplified from the *Escherichia coli* BL21Gold(DE3) genomic DNA and then assembled into pET28(+)

vector. Plasmids for PltG and CalA module1 mutants were generated by site-directed mutagenesis using standard procedures.

For production of compound **6**, a four-gene, three-plasmid system was designed for co-overexpression of PltB module1-DEBS2dd, DEBS3dd-PltB module2, Sfp, and MatB. The DNA fragments encoding for MatB, PltB module1 fused with DEBS2dd and PltB module2 linked to DEBS3dd were amplified using vectors constructed above as the template. The gene encoding for PltB module1-DEBS2dd was first inserted into the second MCS (between NdeI and XhoI restriction sites) of pETDuet-1 vector, followed by the introduction of the *matB* gene into the first MCS (between NcoI and HindIII restriction sites) to construct the recombinant pETDuet-1 vector expressing both MatB and PltB module1 DEBS2dd. The DNA fragment containing PltB module2 DEBS3dd was inserted into the MCS2 between NdeI and XhoI restriction sites of pACYCDuet-1 vector.

Recombinant protein expression and purification

For overexpression of holo-PKSs, the pET28(+) vector carrying PKS modules and the pCDFDuet-1 vector carrying Sfp were co-transformed into *E. coli* BL21Gold(DE3). Overnight cultures were inoculated into 2–4 L terrific both media supplemented with appropriate antibiotics. The cells were grown at 30 ˚C until OD_{600} reached 0.4–0.5 at which time growth temperature was reduced to 18 ˚C. When OD_{600} reached 0.7–0.8, protein expression was induced by the addition of 0.05–0.1 mM IPTG together with 0.25 mM calcium pantothenate. Bacterial cells were cultured at 18 ˚C for an addition of 18 h before harvested by centrifugation at 2,000×gfor 25 min. Cell pellets were stored at −80 ˚C until purification. For MatB, PltG (wild type and mutants), and FabD, expression vectors encoding for proteins of interest were transformed into *E. coli* BL21Gold(DE3). Similar growth condition was used as described above, except that 1 L terrific broth medium was used for cell culture and protein expression was induced with 0.2 mM IPTG.

All steps for protein purifications were performed at 4 ˚C or on ice. Cell pellets were resuspended in binding buffer (20 mM Tris-HCl pH=8.0, 500 mM NaCl, 10% glycerol) and lysed by sonication. The lysate was clarified by centrifugation at 25,000×g for 45 min, and then applied to a 5 mL HisTrap HP column. The column was washed extensively with wash buffer (20 mM Tris-HCl pH=8.0, 30 mM imidazole, 500 mM NaCl, 10% glycerol) till a stable UV-absorbance base line was observed, and then eluted with a linear gradient to 100 % of elution buffer (20 mM Tris-HCl pH=8.0, 250 mM imidazole, 500 mM NaCl, 10% glycerol) over 8 column volumes using ÄKTAprime plus FPLC system. Purity of eluent protein fractions were checked by SDS-PAGE, and fractions containing desired purified proteins were combined. For holo-PKSs, pooled protein solutions were concentrated using 50 kDa Amicon centrifugal filters and

desalted into binding buffer with PD-10 columns. Combined FabD protein solution was dialyzed overnight in 2 L binding buffer before storage. For PltG wild type and mutants, combined protein fractions were dialyzed in 2 L buffer composed of 20 mM Tris-HCl pH=8.9, 50 mM KCl and 10% glycerol overnight. The protein samples after dialysis were loaded to a 5 mL Hi-Trap Q column, washed with 5 column volumes of buffer A (20 mM Tris-HCl pH=8.9, 50 mM KCl), and then eluted with buffer B (20 mM Tris-HCl pH=8.9, 1 M KCl). Eluted protein fractions were checked by SDS-PAGE. Fractions containing protein of interest were combined, glycerol was added to a final concentration of 10% v/v and stored in small aliquots at −80˚C. Fresh aliquots were used each time for enzyme assays.

Enzyme assay for pyrone formation

In PKS assays with CPs, PltB module1 was incubated with its cognate CP partner-PltL, while CalA module1 was paired with CalN3. The preparation of pyrrolyl-*S*-PltL, dichloropyrrolyl-*S*-PltL, pyrrolyl-*S*-CalN3, and dichloropyrrolyl-*S*-CalN3 has been described previously (Fig. S1).[3] Briefly, the assay was composed of 1.2 mM synthesized (dichloro)pyrrolyl acyl-*S*-pantetheines, 10 mM $MgCl_2$, 50 mM HEPES-Na (pH=7.9), 400 μM CPs, 3 μM Sfp, 2 μM CoaA, 2 μM CoaD, and 2 μM CoaE in a total volume of 2.5 mL. The enzymatic reaction was initiated by the addition of 10 mM ATP after incubation at 30 °C for 5 min. The assay was incubated at 30 ˚C for 4.5 h, followed by 4 ˚C overnight, and then desalted into binding buffer (20 mM Tris-HCl pH=8.0, 500 mM NaCl, 10% glycerol) with PD-10 columns.

The PKS assay was performed in a total volume of 200 μL containing 50 μM CP substrates, 5 mM ATP, 10 mM $MgCl_2$, 1.2 mM Na-malonate, 0.6 mM coenzyme A, 5 mM TCEP, 5 μM MatB, 5 μM holo-PltB module1 or 10 μM holo-CalA module1 with equimolar holo-PltB module2, 0 or 5 μM PltG (wild type or mutants), and 400 mM potassium phosphate buffer (pH=7.5). The enzyme assays were incubated at 30 ˚C for 1 h before being quenched with 25 μL 3 M HCl. The quenched reaction mixture was extracted using EtOAc (250 μL, 3×). The combined organic layers were concentrated under vacuum, reconstituted in 100 μL MeOH, and then analyzed by HPLC or LC-MS after clarification by centrifugation. For time-course competitive assay, the assay was performed in a total volume of 1.2 mL supplied with equimolar dichloropyrrolyl- and unsubstituted pyrrolyl-*S*-CPs (25 μM each). For PltB, 200 μL samples were taken from the reaction mixture and quenched with 25 μL 3 M HCl at 1, 2, 5, 10, 15 min. 200 μL samples were taken and quenched at 1, 2, 4, 6, 8 h for CalA reactions. The quenched reaction mixture was extracted with EtOAc (250 μL, 3×), concentrated, and reconstituted in 100 μL MeOH before HPLC analysis. The data points were collected from experiments performed in triplicate.

When acyl-SNACs were used as substrates, the assays were performed in a total volume of 150 µL containing 0.15 mM acyl-SNACs, 5 mM ATP, 10 mM $MgCl_2$, 1.2 mM Na-malonate, 0.6 mM coenzyme A, 5 mM TCEP, 5 µM MatB, 7.5 µM holo-PltB module1 or holo-CalA module1, 7.5 µM holo-PltB module2, and 400 mM potassium phosphate buffer (pH=7.5). For *trans*-AT system, 7.5 µM holo-CalA module1 S633A together with equimolar FabD were added to the enzyme reaction instead of wild-type holo-CalA. The reactions were incubated at 30 ˚C for 4.5 h before being quenched with 75 µL MeOH containing 10% v/v formic acid and 0.03 mM internal standard (4-hydroxy-6-methyl-2-pyrone). For time-course assay, the reactions were performed in a total volume of 400 µL; 70 µL aliquots were withdrawn at 1, 2, 4, 6, 8 h and added to 30 µL MeOH with 10% v/v formic acid. Each experiment was conduct in triplicate. The quenched reactions were analyzed by LC-MS or HPLC.

Organic extraction of *Pseudomonas protegens* Pf-5

The bacterium *P. protegens* Pf-5 was inoculated into 50 mL LB media from glycerol stock and grown at 30 ˚C for 24 h. Cell pellets and liquid media were separated by centrifugation at 2,000×g for 20 min. The supernatant was acidified with 3 M HCl to pH = 2.0, and then extracted with EtOAc (50 mL, 3×). The organic layers were combined, dried with anhydrous Na_2SO_4, and concentrated under vacuum. The concentrated residue was dissolved in 1 mL MeOH and analyzed by HPLC-HRMS in the negative ionization mode.

LC-MS and HPLC analysis

High resolution mass spectrometry (HRMS) data for synthesized acyl-SNACs were collected on an Agilent 1290 Infinity II UHPLC system coupled to a Bruker Impact II ToF mass spectrometer operating at room temperature with Kinetex 1.7 µm C18 100 Å column (50×2.1 mm). Mixture of water and acetonitrile with 0.1% formic acid was used as the mobile phase at a flow rate of 0.5 $mL \cdot min^{-1}$. All MS data for acyl-SNACs were collected in positive mode.

Production of pyrone analogs were monitored by either LC-MS or HPLC. HPLC analyses to monitor the production of **6** and **7** were carried out using Phenomenex Luna 5 µm C8(2) 100 Å LC column (250×4.6 mm) using Agilent 1260 Infinity II HPLC system. Water (solvent A) and MeCN (solvent B) with 0.1 % TFA were used as the mobile phase. A flow rate of 0.5 $mL \cdot min^{-1}$ was used with the following gradient: 0–5 min: 5% B, 5–30 min: linear gradient to 100% B, 30–34 min: 100% B, 34–35 min: linear gradient to 5% B, 35–36 min: 5 % B, 36–37 min: linear gradient to 100% B, 37–38 min: 100% B, 38–39 min: linear

gradient to 5% B. UV absorbance was monitored at 352 nm. LC-MS analyses were carried out on Agilent Poroshell 120 EC-C18 column (2.7 µm, 4.6 × 100 mm) using Agilent 1260 Infinity HPLC coupled to a Bruker amaZon SL operating in the negative ionization mode. Water (solvent A) and MeCN (solvent B) with 0.1 % formic acid were used as the mobile phase. A flow rate of 0.5 mL·min^{-1} was used with the following gradient: 0–3 min: 5% B, 3–16 min: linear gradient to 100% B, 16–20 min: 100% B, 20–21 min: linear gradient to 5% B, 21–22 min: 5% B, 22–23 min: linear gradient to 100% B, 23–24 min: 100% B, 24–25 min: linear gradient to 5% B, 25–27 min: 5% B. Ions corresponding to pyrones and internal standard were extracted, and peak areas for extracted ion counts were integrated. Ion count ratio between pyrone product and internal standard was calculated, normalized based on monoisotopic abundance, and averaged between three triplicate experiments. To represent relative activity, activity of each Plt PKS system (native or engineered) towards dichloropyrrole-2-carbonyl-SNAC is set as 1. Production level of other pyrone analogs by native or engineered PKSs was normalized to the production level of **6** by each PKS system. The same UHPLC-HRMS system was used to collect HRMS data for pyrone analogs except that the MS data for pyrone analogs were collected in the negative mode.

Organic extracts from *P. protegens* Pf-5 were analyzed by UHPLC-HRMS system operating in negative mode on Thermo Scientific™ Accucore™ Phenyl-X column (2.6 µm, 2.1×50 mm) with the following gradient: 0–2 min: 5% B, 2–12 min: linear gradient to 100% B, 12–16 min: 100% B, 16–17 min: linear gradient to 5% B, 17–18 min: 5% B, 18–19 min: linear gradient to 100% B, 19–20 min: 100% B, 20–21 min: linear gradient to 5% B, 21–22 min: 5% B.

Large scale production of dichloropyrrolyl pyrone

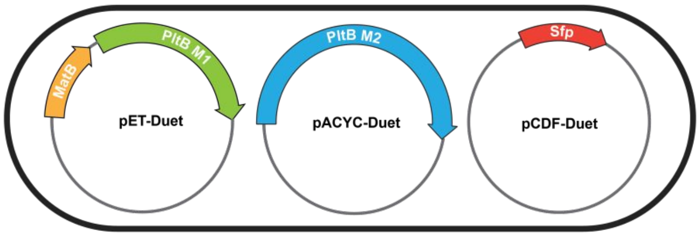

Expression vectors, pETDuet-1 carrying MatB and PltB module1 DEBS2dd, pACYCDuet-DEBS3dd PltB module2 and pCDFDuet-Sfp, were co-transformed into *E. coli* BL21Gold(DE3). BL21 cells with expression plasmids were cultured under the same conditions as described for protein overexpression. After 24 h induction, *E. coli* cells were harvested by centrifugation at 2,000×g for 25 min. Cell pellets collected from 2 L culture were combined, resuspended in 60 mL buffer containing 400 mM potassium phosphate pH=7.5 and 10% glycerol, and sonicated for 40 min. The lysate was clarified by centrifugation

and used directly for large scale enzyme assays which was composed of 10 mM ATP, 10 mM $MgCl_2$, 10 mM Na-malonate, 0.2 mM coenzyme A, 0.4 mM dichloropyrrole-2-carbonyl-SNAC, 5 mM TCEP, 5 mM calcium pantothenate, 400 mM potassium phosphate pH=7.5 and 10% glycerol in a total volume of 100 mL. ATP, Na-malonate and dichloropyrrole-2-carbonyl-SNAC were divided into 4 batches and added every 2 h. The reaction mixture was shaken at 60 rpm at 30 ˚C for 24 h before quenched with TFA to pH = 2.0. The acidified mixture with precipitate was extracted with EtOAC (100 mL, 3×). The organic and aqueous layers were separated by centrifugation at 1,000×g for 5 min. The organic layers were combined, dried with anhydrous Na_2SO_4, and concentrated under vacuum. The crude extract was first purified by silica flash column (0–100% EtOAc in hexane). Fractions contain desired products (checked by LC-MS) were pooled, concentrated, and further purified by two rounds of preparative HPLC. For the first round of preparative HPLC purification, it was carried out on Luna 5 µm C18(2) 100 Å LC column (250×10 mm) using Agilent 1260 Infinity II HPLC system, using mixture of water (solvent A) and acetonitrile (solvent B) with 0.1 % TFA as the mobile phase. A flow rate of 2 $mL·min^{-1}$ was used with the following gradient: 0–5 min: 5% B, 5–30 min: linear gradient to 50% B, 30–36 min: 50% B, 36–42 min: linear gradient to 100% B, 42–46 min: 100% B, 46–47 min: linear gradient to 5% B, 47–48 min: 5 % B, 48–49 min: linear gradient to 100% B, 49–50 min: 100% B, 50–51 min: linear gradient to 5% B. The second round of purification was achieved on Luna 5 µm C8(2) 100 Å LC column (250×4.6 mm) with isocratic elution at 50% B using the same solvent system at a flow rate of 0.5 $mL·min^{-1}$. The product was monitored at UV 352 and 254 nm. Purified molecule was analyzed by HRMS and NMR spectra.

SUPPLEMENTARY SCHEMES

Synthesis of acyl-SNACS

Scheme S1 synthesis of 4,5-dichloro-1H-pyrrole-2-carbonyl-SNAC (**8a**)

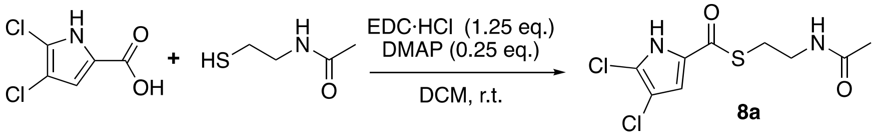

The compound 4,5-dichloro-1H-pyrrole-2-carboxylic acid was synthesized as previous described.[1, 4] Dichloropyrrolyl-2-carboxylic acid (72.7 mg, 0.61 mmol, 1 eq.), N-acetylcysteamine (109.7 mg, 0.61 mmol, 1 eq.), EDC·HCl (146.1 mg, 0.76 mmol, 1.25 eq.), and DMAP (22.3 mg, 0.18 mmol, 0.3 eq.) were dissolved in 3 mL DCM at room temperature. The reaction was stirred at room temperature overnight. The reaction mixture was concentrated under vacuum and purified by silica flash column (50% EtOAc in hexane to 5% MeOH in EtOAc) to give **8a** as yellow solid (27.5 mg, 16 %). ^{1}H NMR (500 MHz, MeOD) δ 8.26 (br s, 1H), 6.93 (s, 1H), 3.39 (td, J = 6.7, 5.0 Hz, 2H), 3.15 (t, J = 6.6 Hz, 2H), 1.93 (s, 3H). ^{13}C NMR (126 MHz, MeOD) δ 180.91, 173.53, 128.98, 120.91, 115.16, 111.75, 40.41, 28.47, 22.48. HRMS (ESI) *m/z* calculated for $C_9H_{11}Cl_2N_2O_2S$ ($[M+H]^+$) 280.9913, found 280.9915.

Scheme S2 synthesis of 1H-pyrrole-2-carbonyl-SNAC (**8b**)

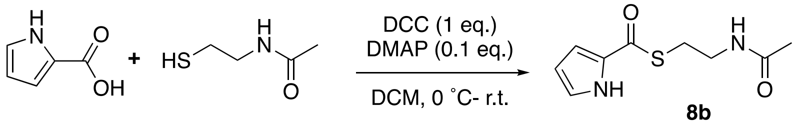

The molecule pyrrolyl-2-carbonyl-SNAC was prepared as described in literature and the ^{1}H NMR spectra matched the previous report.[5] Pyrrole-2-carboxylic acid (300 mg, 2.7 mmol, 1 eq.), N-acetylcysteamine (321.9 mg, 2.7 mmol, 1 eq.), and DMAP (33.0 mg, 0.27 mmol, 0.1 eq.) were dissolved in 8.2 mL dry DCM at 0 ˚C. A solution of DCC (557.2 mg, 2.7 mmol, 1 eq.) dissolved in 5.4 mL dry DCM was then added dropwise at 0 ˚C. The reaction mixture was stirred overnight from 0 ˚C to room temperature before concentration under vacuum. The crude mixture was purified by silica flash column (50% EtOAc in hexane to EtOAc) to give **8b** as white solid (272.3 mg, 48 %). ^{1}H NMR (400 MHz, $CDCl_3$) δ 9.34 (br s, 1H), 7.03 (tdd, J = 3.9, 2.6, 1.3 Hz, 2H), 6.29 (dt, J = 3.9, 2.5 Hz, 1H), 6.13 (br s, 1H), 3.52 (dt, J = 6.5, 5.6 Hz, 2H), 3.19 (dd, J = 6.9, 5.7 Hz, 2H), 1.98 (s, 3H). HRMS (ESI) *m/z* calculated for $C_9H_{13}N_2O_2S$ ($[M+H]^+$) 213.0692, found 213.0694.

Scheme S3 synthesis of 4-chloro-1H-pyrrole-2-carbonyl-SNAC (**8c**)

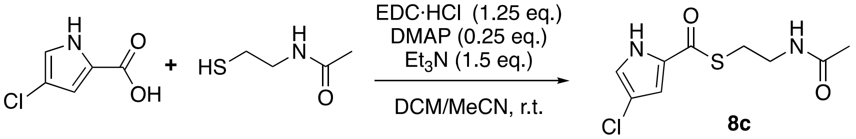

4-Chloro-1H-pyrrole-2-carboxylic acid (60 mg, 0.41 mmol, 1 eq.), N-acetylcysteamine (49.1 mg, 0.41 mmol, 1 eq.), EDC·HCl(98.8 mg, 0.52 mmol, 1.25 eq.), and DMAP (12.6 mg, 0.10 mmol, 0.25 eq.) were dissolved in 4.2 mL 2:1 DCM/acetonitrile at room temperature, followed by the addition of Et_3N (62.6 mg, 0.62 mmol, 1.5 eq.). The reaction was stirred at room temperature overnight before concentrated under vacuum. The reaction mixture was first purified by silica flash chromatography (20% hexane in EtOAc to EtOAc), followed by preparative HPLC purification to give **8c** as white solid (4.1 mg, 4 %). Preparative HPLC purification was carried out on Luna 5 µm C18(2) 100 Å LC column (250×10 mm) using Agilent 1260 Infinity II HPLC system using mixture of water (buffer A) and acetonitrile (buffer A) containing 0.1% TFA as the mobile phase. A flow rate of 2 $mL{\cdot}min^{-1}$ was used with the following gradient: 0-5 min: 5% B, 5-30 min: linear gradient to 60% B, 30-36 min: 60% B, 36-38 min: linear gradient to 100% B, 38-43 min: 100% B, 43-44 min: linear gradient to 5% B, 44-45 min: 5 % B, 45-46 min: linear gradient to 100% B, 46-47 min: 100% B, 47-48 min: linear gradient to 5% B. ^1H NMR (500 MHz, MeOD) δ 7.02 (d, J = 1.6 Hz, 1H), 6.87 (d, J = 1.6 Hz, 1H), 3.39 (t, J = 6.6 Hz, 2H), 3.14 (t, J = 6.6 Hz, 2H), 1.93 (s, 3H). ^{13}C NMR (126 MHz, MeOD) δ 181.63, 173.52, 130.55, 123.02, 114.69, 114.60, 40.50, 28.40, 22.48. HRMS (ESI) *m/z* calculated for $C_9H_{12}ClN_2O_2S([M+H]^+)$ 247.0303, found 247.0306

Scheme S4 synthesis of 5-methyl-1H-pyrrole-2-carbonyl-SNAC (**8d**)

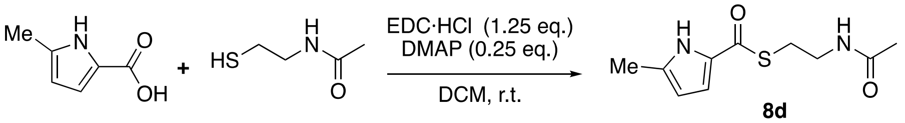

5-Methyl-1H-pyrrole-2-carboxylic acid was prepared as previously described.[1, 6] 5-Methyl-pyrrole-2-carboxylic acid (100 mg, 0.80 mmol, 1 eq.), N-acetylcysteamine (95.3 mg, 0.80 mmol, 1 eq.), EDC·HCl (191.5 mg, 1.0 mmol, 1.25 eq.), and DMAP (24.4 mg, 0.20 mmol, 0.25 eq.) were dissolved in 4 mL DCM at room temperature. The reaction was stirred at room temperature overnight before concentrated under vacuum. The crude mixture was purified by silica flash chromatography (DCM to 5% MeOH in DCM) to give **8d** as white solid (115.9 mg, 64 %). ^1H NMR (400 MHz, $CDCl_3$) δ 8.95 (br s, 1H), 6.94 (dd, J = 3.9, 2.5 Hz, 1H), 6.30 (br s, 1H), 5.99 (ddd, J = 3.7, 2.7, 0.8 Hz, 1H), 3.52 (q, J = 5.8 Hz, 2H), 3.17 (dd, J = 6.9, 5.6 Hz, 2H), 2.32 (s, 3H), 2.01 (s, 3H). ^{13}C NMR (126 MHz, $CDCl_3$) δ 180.83, 170.77, 135.85, 128.68,

117.27, 110.01, 40.49, 27.72, 23.26, 13.38. HRMS (ESI) *m/z* calculated for $C_{10}H_{15}N_2O_2S$ ($[M+H]^+$) 227.0849, found 227.0852.

Scheme S5 synthesis of 4,5-dimethyl-1H-pyrrole-2-carbonyl-SNAC (**8e**)

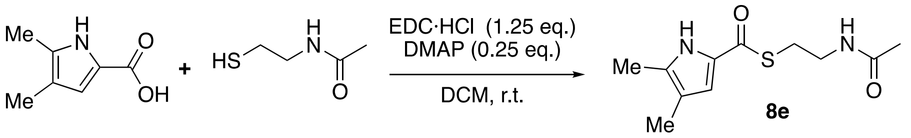

4, 5-Dimethyl-1H-pyrrole-2-carboxylic acid (60 mg, 0.43 mmol, 1 eq.), N-acetylcysteamine (51.4 mg, 0.43 mmol, 1 eq.), EDC·HCl (103.3 mg, 0.54 mmol, 1.25 eq.), and DMAP (13.2 mg, 0.11 mmol, 0.25 eq.) were dissolved in 2.2 mL DCM at room temperature. The reaction was stirred at room temperature overnight before concentrated under vacuum. The crude mixture was purified by silica flash chromatography (20% hexane in EtOAc to 2% MeOH in EtOAc) to give **8e** as pink solid (76.7 mg, 74 %). ^{1}H NMR (500 MHz, $CDCl_3$) δ 9.04 (br s, 1H), 6.80 (d, *J* = 2.7 Hz, 1H), 6.23 (br s, 1H), 3.50 (q, *J* = 5.8 Hz, 2H), 3.15 (dd, *J* = 6.8, 5.6 Hz, 2H), 2.22 (s, 3H), 2.01 (s, 3H), 1.97 (s, 3H). ^{13}C NMR (126 MHz, $CDCl_3$) δ 180.37, 170.72, 133.15, 126.95, 118.66, 117.91, 40.58, 27.66, 23.27, 11.57, 10.95. HRMS (ESI) *m/z* calculated for $C_{11}H_{17}N_2O_2S$ ($[M+H]^+$) 241.1005, found 241.1008

Scheme S6 synthesis of furan-2-carbonyl-SNAC (**8f**)

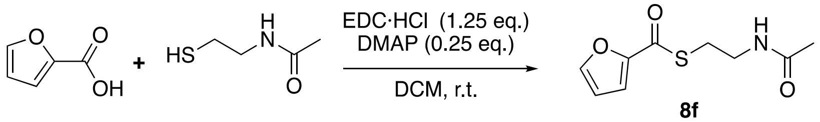

2-Furoic acid (200 mg, 1.78 mmol, 1 eq.), N-acetylcysteamine (255.2 mg, 2.14 mmol, 1.2 eq.), EDC·HCl (427.6 mg, 2.23 mmol, 1.25 eq.), and DMAP (54.5 mg, 0.45 mmol, 0.25 eq.) were dissolved in 8.9 mL DCM at room temperature. The reaction was stirred at room temperature overnight before the addition of saturated NH_4Cl (10 mL). The organic phase was separated and washed with saturated $NaHCO_3$ (10 mL). The aqueous layer was extracted with EtOAc (10 mL, 3×). The combined organic layers were dried with anhydrous Na_2SO_4, concentrated under vacuum and the purified by silica flash chromatography (90% EtOAc in hexane to 5% MeOH in EtOAc) to give **8f** as white solid (312.2 mg, 82 %). ^{1}H NMR (400 MHz, $CDCl_3$) δ 7.60 (dd, *J* = 1.7, 0.8 Hz, 1H), 7.22 (dd, *J* = 3.6, 0.8 Hz, 1H), 6.56 (dd, *J* = 3.6, 1.7 Hz, 1H), 6.07 (br s, 1H), 3.57 – 3.48 (m, 2H), 3.21 (dd, *J* = 6.8, 5.9 Hz, 2H), 1.99 (s, 3H). ^{13}C NMR (126 MHz, $CDCl_3$) δ

180.81, 170.71, 150.65, 146.70, 116.32, 112.56, 39.93, 27.84, 23.29. HRMS (ESI) *m/z* calculated for $C_9H_{12}NO_3S$ ([M+H]$^+$) 214.0532, found 214.0534

Scheme S7 synthesis of 4,5-dibromofuran-2-carbonyl-SNAC (**8g**)

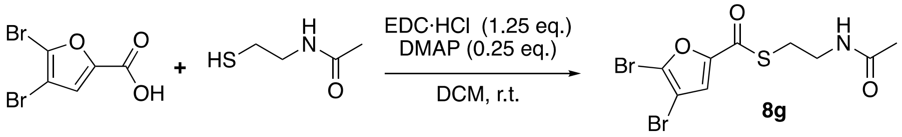

4,5-Dibromofuran-2-carboxylic acid (300 mg, 1.11 mmol, 1 eq.), N-acetylcysteamine (132.5 mg, 1.11 mmol, 1 eq.), EDC·HCl (266.4 mg, 1.39 mmol, 1.25 eq.), and DMAP (34.0 mg, 0.28 mmol, 0.25 eq.) were dissolved in 5.6 mL DCM at room temperature. The reaction was stirred at room temperature overnight before the addition of saturated NH_4Cl (10 mL). The organic phase was separated and washed with saturated $NaHCO_3$ (10 mL). The aqueous layer was extracted with EtOAc (10 mL, 3×). The combined organic layers were dried with anhydrous Na_2SO_4, concentrated under vacuum and the purified by silica flash chromatography (75% EtOAc in hexane to 5% MeOH in EtOAc) to give **8g** as white solid (249.9 mg, 61 %). ^{1}H NMR (400 MHz, $CDCl_3$) δ 7.20 (s, 1H), 6.01 (br s, 1H), 3.52 (q, *J* = 6.1 Hz, 2H), 3.22 (t, *J* = 6.4 Hz, 2H), 1.99 (s, 3H). ^{13}C NMR (126 MHz, $CDCl_3$) δ 179.21, 170.81, 151.62, 129.16, 119.86, 104.86, 39.65, 28.12, 23.25. HRMS (ESI) *m/z* calculated for $C_9H_{10}Br_2NO_3S$ ([M+H]$^+$) 369.8743, found 369.8739.

Scheme S8 synthesis of 5-bromofuran-2-carbonyl-SNAC (**8h**)

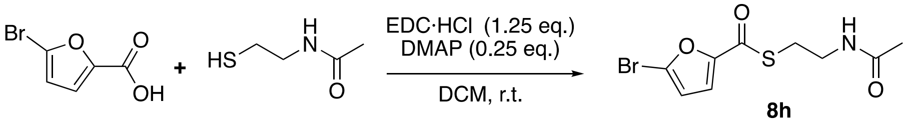

5-Bromofuran-2-carboxylic acid (180 mg, 0.94 mmol, 1 eq.), N-acetylcysteamine (112.3 mg, 0.94 mmol, 1 eq.), EDC·HCl (225.9 mg, 1.18 mmol, 1.25 eq.), and DMAP (28.8 mg, 0.24 mmol, 0.25 eq.) were dissolved in 4.7 mL DCM at room temperature. The reaction was stirred at room temperature for 2 days before quenched with saturated NH_4Cl (10 mL). The organic phase was then washed with saturated $NaHCO_3$ (10 mL). The aqueous layer was extracted with EtOAc (10 mL, 4×). The combined organic layers were dried with anhydrous Na_2SO_4, concentrated under vacuum and the purified by silica flash chromatography (80% EtOAc in hexane to 90% EtOAc in hexane) to give **8h** as white solid (207.8 mg, 75 %). ^{1}H NMR (400 MHz, $CDCl_3$) δ 7.15 (d, *J* = 3.6 Hz, 1H), 6.50 (d, *J* = 3.6 Hz, 1H), 6.05 (br s, 1H), 3.52 (q, *J* = 6.2 Hz, 2H), 3.21 (dd, *J* = 6.8, 5.9 Hz, 2H), 1.99 (s, 3H). ^{13}C NMR (126 MHz, $CDCl_3$) δ 179.54,

170.78, 152.14, 128.62, 118.27, 114.64, 39.80, 27.96, 23.28. HRMS (ESI) *m/z* calculated for $C_9H_{11}BrNO_3S$ ($[M+H]^+$) 291.9638, found 291.9636.

Scheme S9 synthesis of thiophene-2-carbonyl-SNAC (**8i**)

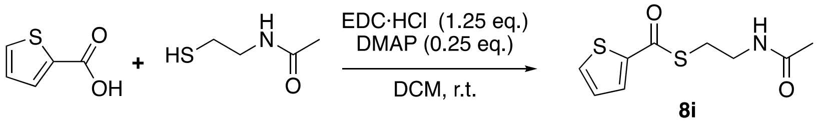

8i

Thiophene-2-carboxylic acid (100 mg, 0.78 mmol, 1 eq.), N-acetylcysteamine (93.0 mg, 0.78 mmol, 1 eq.), EDC·HCl (187.0 mg, 0.98 mmol, 1.25 eq.), and DMAP (23.8 mg, 0.20 mmol, 0.25 eq.) were dissolved in 3.9 mL DCM at room temperature. The reaction was stirred at room temperature overnight before the addition of saturated NH_4Cl (10 mL). The organic phase was separated and washed with saturated $NaHCO_3$ (10 mL). The aqueous layer was extracted with EtOAc (10 mL, 3×). The combined organic layers were dried with anhydrous Na_2SO_4, concentrated under vacuum and the purified by silica flash chromatography (90% EtOAc in hexane to 5% MeOH in EtOAc) to give **8i** as white solid (159.5 mg, 89 %). ^{1}H NMR (500 MHz, $CDCl_3$) δ 7.81 (d, J = 3.9 Hz, 1H), 7.65 (d, J = 4.9 Hz, 1H), 7.13 (t, J = 4.4 Hz, 1H), 6.08 (br s, 1H), 3.53 (q, J = 6.1 Hz, 2H), 3.22 (t, J = 6.4 Hz, 2H), 1.98 (s, 3H). ^{13}C NMR (126 MHz, $CDCl_3$) δ 184.40, 170.67, 141.69, 133.36, 131.75, 128.21, 39.96, 28.86, 23.35. HRMS (ESI) *m/z* calculated for $C_9H_{12}NO_2S_2$ ($[M+H]^+$) 230.0304, found 230.0301

Scheme S10 synthesis of 4,5-dichlorothiophene-2-carbonyl-SNAC (**8j**)

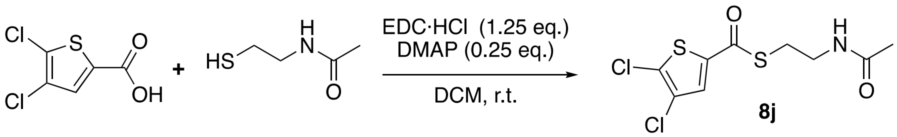

8j

4,5-Dichlorothiophene-2-carboxylic acid (180 mg, 0.91 mmol, 1 eq.), N-acetylcysteamine (108.9 mg, 0.91 mmol, 1 eq.), EDC·HCl (218.9 mg, 1.14 mmol, 1.25 eq.), and DMAP (27.9 mg, 0.23 mmol, 0.25 eq.) were dissolved in 4.6 mL DCM at room temperature. The reaction was stirred at room temperature for 2 days before the addition of saturated NH_4Cl (10 mL). The organic phase was separated and washed with saturated $NaHCO_3$ (10 mL). The aqueous layer was extracted with EtOAc (10 mL, 3×). The combined organic layers were dried with anhydrous Na_2SO_4, concentrated under vacuum and the purified by silica flash chromatography (80% EtOAc in hexane to 90% EtOAc in hexane) to give **8j** as white solid (209.6 mg, 77 %). ^{1}H NMR (500 MHz, $CDCl_3$) δ 7.58 (s, 1H), 5.97 (br s, 1H), 3.52 (q, J = 6.2 Hz, 2H), 3.23 (t, J = 6.4

Hz, 2H), 1.99 (s, 3H). ^{13}C NMR (126 MHz, $CDCl_3$) δ 183.01, 170.67, 137.37, 133.60, 130.70, 125.55, 39.67, 29.02, 23.33. HRMS (ESI) *m/z* calculated for $C_9H_{10}Cl_2NO_2S_2$ ($[M+H]^+$) 297.9525, found 297.9522.

Scheme S11 synthesis of 5-chlorothiophene-2-carbonyl-SNAC (**8k**)

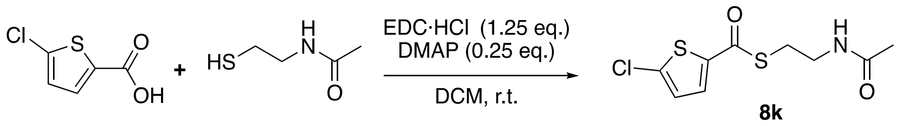

5-Chlorothiophene-2-carboxylic acid (200 mg, 1.23 mmol, 1 eq.), N-acetylcysteamine (146.6 mg, 1.23 mmol, 1 eq.), EDC·HCl (294.8 mg, 1.54 mmol, 1.25 eq.), and DMAP (37.6 mg, 0.31 mmol, 0.25 eq.) were dissolved in 6.2 mL DCM at room temperature. The reaction was stirred at room temperature overnight before the addition of saturated NH_4Cl (10 mL). The organic phase was separated and washed with saturated $NaHCO_3$ (10 mL). The aqueous layer was extracted with EtOAc (10 mL, 3×). The combined organic layers were dried with anhydrous Na_2SO_4, concentrated under vacuum and the purified by silica flash chromatography (80% DCM in hexane to 5% MeOH in DCM) to give **8k** as white solid (279.0 mg, 86 %). ^1H NMR (500 MHz, $CDCl_3$) δ 7.59 (d, J = 4.1 Hz, 1H), 6.95 (d, J = 4.1 Hz, 1H), 6.20 (br s, 1H), 3.53 – 3.49 (m, 2H), 3.21 (dd, J = 6.8, 6.1 Hz, 2H), 1.98 (s, 3H). ^{13}C NMR (126 MHz, $CDCl_3$) δ 183.39, 170.74, 139.88, 139.30, 131.20, 127.66, 40.43, 28.80, 23.25. HRMS (ESI) *m/z* calculated for $C_9H_{11}ClNO_2S_2$ ($[M+H]^+$) 263.9914, found 263.9911.

Scheme S12 synthesis of thiazole-4-carbonyl-SNAC (**8l**)

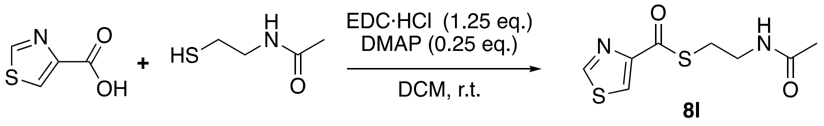

Thiazole-4-carbonyl-SNAC was synthesized as previously described and the spectra matched the previous report.[7] Thiazole-4-carboxylic acid (100 mg, 0.77 mmol, 1 eq.), N-acetylcysteamine (92.3 mg, 0.77 mmol, 1 eq.), EDC·HCl (185.6 mg, 0.97 mmol, 1.25 eq.), and DMAP (23.7 mg, 0.19 mmol, 0.25 eq.) were dissolved in 3.9 mL DCM at room temperature. The reaction was stirred at room temperature overnight before the addition of saturated NH_4Cl (10 mL). The organic phase was separated and washed with saturated $NaHCO_3$ (10 mL). The aqueous layer was extracted with EtOAc (10 mL, 3×). The combined organic layers were dried with anhydrous Na_2SO_4, concentrated under vacuum and the purified by silica flash chromatography (75% EtOAc in hexane to 10% MeOH in EtOAc) to give **8l** as white solid (126.9 mg, 71 %). ^1H NMR (400 MHz, $CDCl_3$) δ 8.87 (d, J = 2.1 Hz, 1H), 8.19 (d, J = 2.1 Hz, 1H), 5.99 (br s, 1H),

3.59 – 3.52 (m, 2H), 3.24 (dd, *J* = 6.8, 5.7 Hz, 2H), 1.99 (s, 5H). HRMS (ESI) *m/z* calculated for $C_8H_{11}N_2O_2S_2$ ($[M+H]^+$) 231.0256, found 231.0258

Scheme S13 synthesis of oxazole-4-carbonyl-SNAC (**8m**)

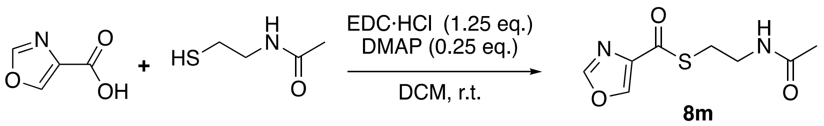

Oxazole-4-carbonyl-SNAC was synthesized as previously described and the spectra matched the previous report.[7] Oxazole-4-carboxylic acid (150 mg, 1.33 mmol, 1 eq.), N-acetylcysteamine (158.1 mg, 1.33 mmol, 1 eq.), EDC·HCl (317.9 mg, 1.66 mmol, 1.25 eq.), and DMAP (40.5 mg, 0.33 mmol, 0.25 eq.) were dissolved in 6.6 mL DCM at room temperature. The reaction was stirred at room temperature overnight before the addition of saturated NH_4Cl (10 mL). The organic phase was separated and washed with saturated $NaHCO_3$ (10 mL). The aqueous layer was extracted with EtOAc (10 mL, 3×). The combined organic layers were dried with anhydrous Na_2SO_4, concentrated under vacuum and the purified by silica flash chromatography (DCM to 5% MeOH in DCM) to give **8m** as white solid (217.9 mg, 77 %).^{1}H NMR (400 MHz, $CDCl_3$) δ 8.29 (d, *J* = 1.0 Hz, 1H), 7.96 (d, *J* = 1.0 Hz, 1H), 6.12 (br s, 1H), 3.59 – 3.53 (m, 2H), 3.25 (dd, *J* = 6.8, 5.8 Hz, 2H), 2.03 (s, 3H). HRMS (ESI) *m/z* calculated for $C_8H_{11}N_2O_3S$ ($[M+H]^+$) 215.0485, found 215.0486

Scheme S14 synthesis of picolinoyl-SNAC (**8n**)

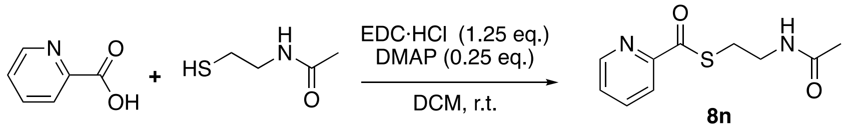

Picolinoyl-SNAC was synthesized as previously described and the spectra matched the previous report.[7] Picolinic acid (154.9 mg, 1.26 mmol, 1 eq.), N-acetylcysteamine (150 mg, 1.26 mmol, 1 eq.), EDC·HCl (301.6 mg, 1.57 mmol, 1.25 eq.), and DMAP (38.4 mg, 0.32 mmol, 0.25 eq.) were dissolved in 6.3 mL DCM at room temperature. The reaction was stirred at room temperature overnight before the addition of saturated NH_4Cl (10 mL). The organic phase was separated and washed with saturated $NaHCO_3$ (10 mL). The aqueous layer was extracted with EtOAc (10 mL, 3×). The combined organic layers were dried with anhydrous Na_2SO_4, concentrated under vacuum and the purified by silica flash chromatography (90% EtOAc in hexane to 10% MeOH in EtOAc) to give **8n** as white solid (226.0 mg, 80 %). ^{1}H NMR (400 MHz, $CDCl_3$) δ 8.71 (ddd, *J* = 4.8, 1.7, 0.9 Hz, 1H), 7.97 (dt, *J* = 7.8, 1.1 Hz, 1H), 7.88 (td, *J* = 7.7, 1.7 Hz,

1H), 7.55 (ddd, J = 7.5, 4.7, 1.3 Hz, 1H), 5.98 (br s, 1H), 3.58 – 3.51 (m, 2H), 3.22 (dd, J = 6.9, 5.7 Hz, 2H), 1.98 (s, 3H). HRMS (ESI) *m/z* calculated for $C_{10}H_{13}N_2O_2S$ ($[M+H]^+$) 225.0692, found 225.0694

Scheme S15 synthesis of nicotinoyl-SNAC (**8o**)

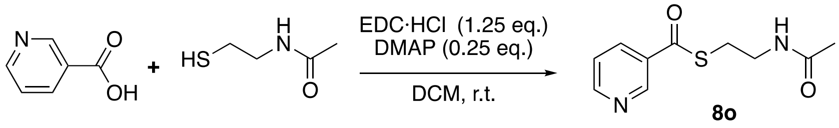

Nicotinoyl-SNAC was synthesized as previously described and the spectra matched the previous report.[7] Nicotinic acid (150 mg, 1.22 mmol, 1 eq.), N-acetylcysteamine (145.2 mg, 1.22 mmol, 1 eq.), EDC·HCl (292.0 mg, 1.52 mmol, 1.25 eq.), and DMAP (37.2 mg, 0.31 mmol, 0.25 eq.) were dissolved in 6.1 mL DCM at room temperature. The reaction was stirred at room temperature overnight before the addition of saturated NH_4Cl (10 mL). The organic phase was separated and washed with saturated $NaHCO_3$ (10 mL). The aqueous layer was extracted with EtOAc (10 mL, 3×). The combined organic layers were dried with anhydrous Na_2SO_4, concentrated under vacuum and the purified by silica flash chromatography (90% EtOAc in hexane to 15% MeOH in EtOAc) to give **8o** as white solid (231.8 mg, 85 %). ^{1}H NMR (500 MHz, $CDCl_3$) δ 9.21 (d, J = 2.1 Hz, 1H), 8.87 – 8.82 (m, 1H), 8.36 (dt, J = 8.0, 1.9 Hz, 1H), 7.61 – 7.54 (m, 1H), 5.98 (br s, 1H), 3.56 (q, J = 6.3 Hz, 2H), 3.30 (t, J = 6.5 Hz, 2H), 1.99 (s, 3H). HRMS (ESI) *m/z* calculated for $C_{10}H_{13}N_2O_2S$ ($[M+H]^+$) 225.0692, found 225.0695.

Scheme S16 synthesis of isonicotinoyl SNAC (**8p**)

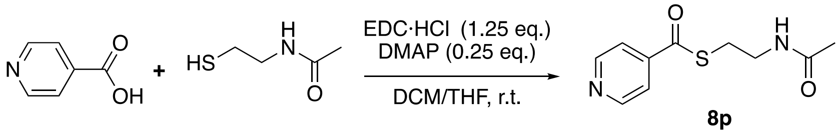

Isonicotinic acid (150 mg, 1.22 mmol, 1 eq.), N-acetylcysteamine (145.2 mg, 1.22 mmol, 1 eq.), EDC·HCl (292.0 mg, 1.52 mmol, 1.25 eq.), and DMAP (37.2 mg, 0.31 mmol, 0.25 eq.) were dissolved in 6.1 mL DCM and 1.5 mL THF at room temperature. The reaction was stirred at room temperature overnight before the addition of saturated NH_4Cl (10 mL). The organic phase was separated and washed with saturated $NaHCO_3$ (10 mL). The aqueous layer was extracted with EtOAc (10 mL, 3×). The combined organic layers were dried with anhydrous Na_2SO_4, concentrated under vacuum and the purified by silica flash chromatography (EtOAc to 10% MeOH in EtOAc) to give **8p** as white solid (231.8 mg, 85 %). ^{1}H NMR (400 MHz, $CDCl_3$) δ 8.84 – 8.79 (m, 2H), 7.82 – 7.77 (m, 2H), 5.94 (br s, 1H), 3.54 (q, J = 6.3 Hz, 2H),

3.28 (t, J = 6.5 Hz, 2H), 1.98 (s, 3H). ^{13}C NMR (126 MHz, $CDCl_3$) δ 191.24, 170.54, 150.18, 143.58, 120.82, 39.30, 29.12, 23.38. HRMS (ESI) *m/z* calculated for $C_{10}H_{13}N_2O_2S$ ($[M+H]^+$) 225.0692, found 225.0693.

Scheme S17 synthesis of benzoyl-SNAC (**8q**)

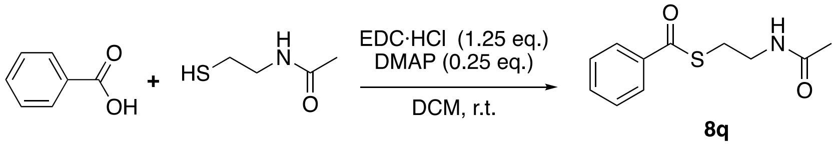

8q

Benzoyl-SNAC was synthesized as previously described and the spectra matched the previous report.[7] Benzoic acid (150 mg, 1.23 mmol, 1 eq.), N-acetylcysteamine (146.4 mg, 1.23 mmol, 1 eq.), EDC·HCl (294.3 mg, 1.54 mmol, 1.25 eq.), and DMAP (37.5 mg, 0.31 mmol, 0.25 eq.) were dissolved in 6.1 mL DCM at room temperature. The reaction was stirred at room temperature overnight before the addition of saturated NH_4Cl (10 mL). The organic phase was separated and washed with saturated $NaHCO_3$ (10 mL). The aqueous layer was extracted with EtOAc (10 mL, 3×). The combined organic layers were dried with anhydrous Na_2SO_4, concentrated under vacuum and the purified by silica flash chromatography (75% EtOAc in hexane to 5% MeOH in EtOAc) to give **8q** as white solid (260.5 mg, 95 %). ^1H NMR (400 MHz, $CDCl_3$) δ 7.99 – 7.94 (m, 2H), 7.62 – 7.57 (m, 1H), 7.50 – 7.44 (m, 2H), 5.93 (br s, 1H), 3.57 – 3.51 (m, 2H), 3.24 (dd, J = 6.9, 5.9 Hz, 2H), 1.98 (s, 3H). HRMS (ESI) *m/z* calculated for $C_{11}H_{14}NO_2S$ ($[M+H]^+$) 224.0740, found 224.0744

Scheme S18 synthesis of isobutyryl-SNAC (**8r**)

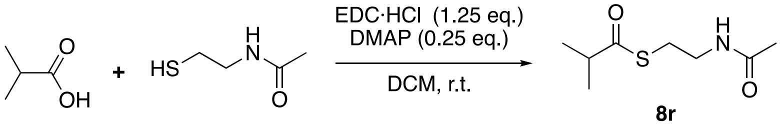

8r

Isobutyric acid (120 mg, 1.36 mmol, 1 eq.), N-acetylcysteamine (162.3 mg, 1.36 mmol, 1 eq.), EDC·HCl (326.4 mg, 1.70 mmol, 1.25 eq.), and DMAP (41.6 mg, 0.34 mmol, 0.25 eq.) were dissolved in 6.8 mL DCM at room temperature. The reaction was stirred at room temperature overnight. The reaction mixture was concentrated under vacuum and purified by silica flash column (80% EtOAc in hexane to 90% EtOAc in hexane) to give **8r** as colorless oil (207.4 mg, 80 %). ^1H NMR (400 MHz, $CDCl_3$) δ 5.97 (br s, 1H), 3.47 – 3.39 (m, 2H), 3.01 (dd, J = 6.9, 6.0 Hz, 2H), 2.76 (m, 1H), 1.97 (s, 3H), 1.19 (d, J = 6.9 Hz, 6H). ^{13}C

NMR (126 MHz, $CDCl_3$) δ 204.81, 170.61, 43.25, 39.85, 28.19, 23.19, 19.46. HRMS (ESI) *m/z* calculated for $C_8H_{16}NO_2S$ ($[M+H]^+$) 190.0896, found 190.0896.

Scheme S19 synthesis of isovaleryl-SNAC (**8s**)

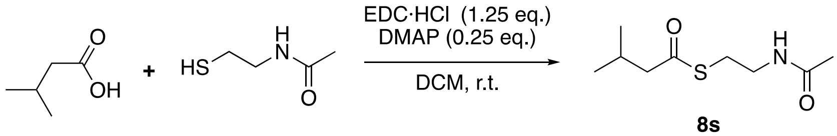

8s

Isovaleric acid (150 mg, 1.47 mmol, 1 eq.), N-acetylcysteamine (175.2 mg, 1.47 mmol, 1 eq.), EDC·HCl (352.2 mg, 1.84 mmol, 1.25 eq.), and DMAP (44.8 mg, 0.37 mmol, 0.25 eq.) were dissolved in 7.3 mL DCM at room temperature. The reaction was stirred at room temperature overnight. The reaction mixture was concentrated under vacuum and purified by silica flash column (80% EtOAc in hexane to 90% EtOAc in hexane) to give **8s** as colorless oil (293.3 mg, 98 %). ^{1}H NMR (500 MHz, $CDCl_3$) δ 6.14 (br s, 1H), 3.40 (q, J = 6.2 Hz, 2H), 3.00 (t, J = 6.4 Hz, 2H), 2.47 – 2.40 (m, 2H), 2.13 (m, 1H), 1.94 (s, 3H), 0.93 (dd, J = 6.8, 2.0 Hz, 6H). ^{13}C NMR (126 MHz, $CDCl_3$) δ 199.72, 170.57, 52.95, 39.90, 28.45, 26.59, 23.21, 22.31. HRMS (ESI) *m/z* calculated for $C_9H_{18}NO_2S$ ($[M+H]^+$) 204.1053, found 204.1055.

SUPPLEMENTARY TABLES

Table S1: NMR assignments of **6** in DMSO-d_6

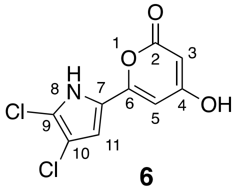

6

position	δ_H (*J* in Hz)	δ_C
2	-	162.5
3	5.26 (d, 2.0)	88.4
4	-	170.7
5	6.47 (d, 2.0)	95.0
6	-	153.4
7	-	122.8
9	-	116.0
10	-	109.7
11	6.86 (d, 2.7)	110.1
NH	12.99 – 12.90 (m)	-
OH	11.93 (s)	-

Table S2: Relative activity of native and engineered system towards a library of synthesized acyl-SNACs

Substrate	Engineered	Native
**8a**	1 ± 0.13	1 ± 0.04
**8b**	0.06 ± 0.01	0.00 ± 0.00
**8c**	0.39 ± 0.05	0.14 ± 0.04
**8d**	0.03 ± 0.00	0.00 ± 0.00
**8e**	0.01 ± 0.00	0.04 ± 0.01
**8f**	0.14 ± 0.02	0.02 ± 0.00
**8g**	3.16 ± 0.36	0.89 ± 0.23
**8h**	1.32 ± 0.17	0.36 ± 0.04
**8i**	0.45 ± 0.05	0.03 ± 0.01
**8j**	2.42 ± 0.60	1.03 ± 0.17
**8k**	2.96 ± 0.45	0.34 ± 0.08
**8l**	0.06 ± 0.00	0.01 ± 0.00
**8m**	0.02 ± 0.00	0.00 ± 0.00
**8n**	0.00 ± 0.00	0.00 ± 0.00
**8o**	0.23 ± 0.04	0.02 ± 0.00
**8p**	0.23 ± 0.00	0.02 ± 0.00
**8q**	0.28 ± 0.03	0.03 ± 0.00
**8r**	0.12 ± 0.00	Not detected
**8s**	0.38 ± 0.02	0.00 ± 0.00

SUPPLEMENTARY FIGURES

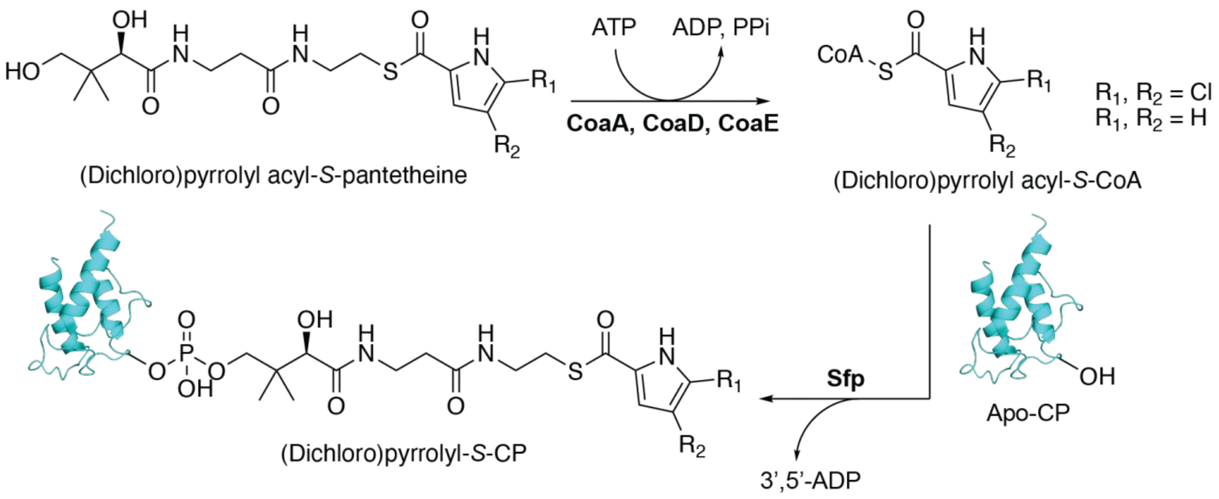

Fig. S1: Scheme for the one-pot chemoenzymatic synthesis of (dichloro)pyrrolyl-*S*-CPs starting from acyl-*S*-pantetheines using CoaA, CoaD, CoaE, and Sfp enzymes.

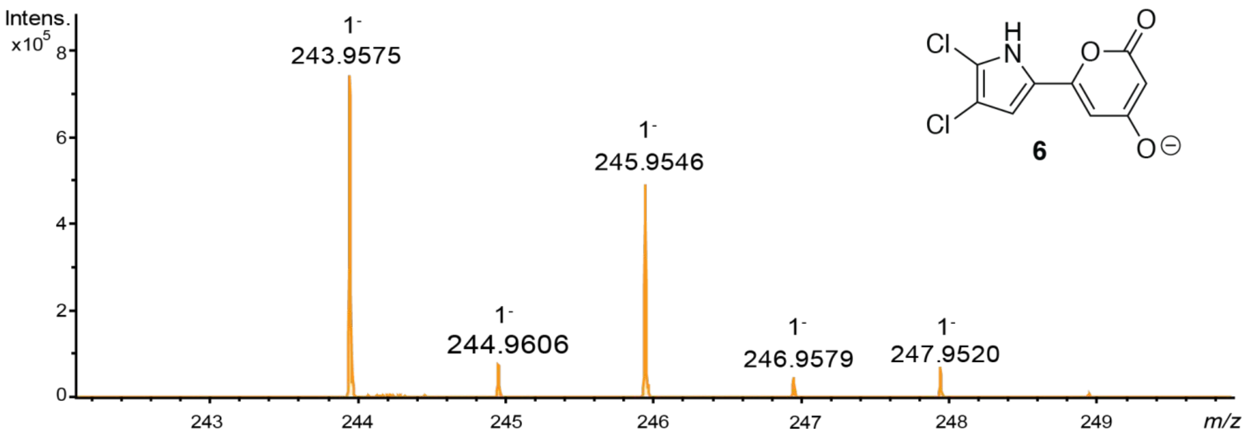

Fig. S2: $[M\text{-}H]^{1-}$ MS^1 spectra of compound **6**. HRMS (ESI) *m/z* calculated for $C_9H_4Cl_2NO_3$ ($[M\text{-}H]^-$) 243.9574, found 243.9575. Isotopic distribution pattern indicates the presence of two chlorine atoms.

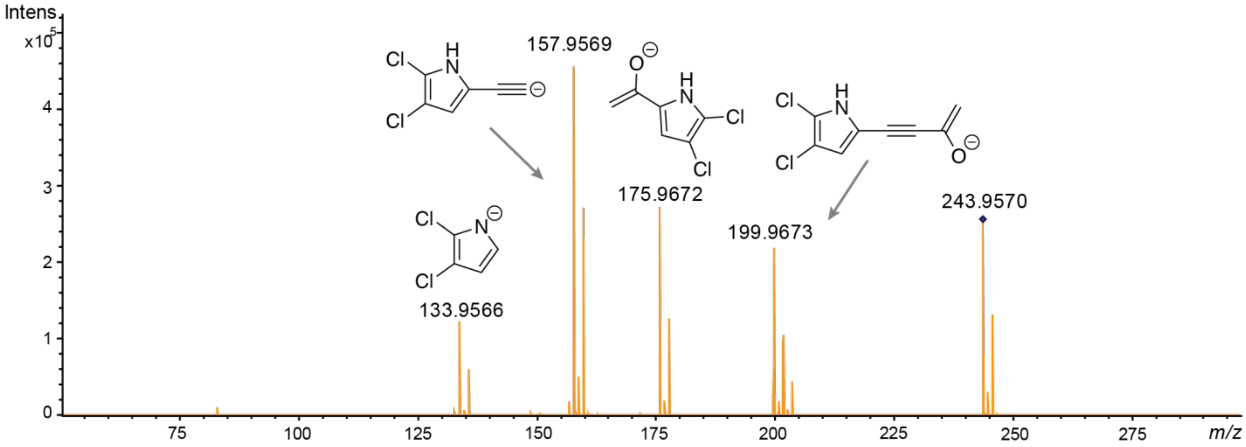

Fig. S3: MS^2 spectra of compound **6** with rationalized structural annotations of fragment ions.

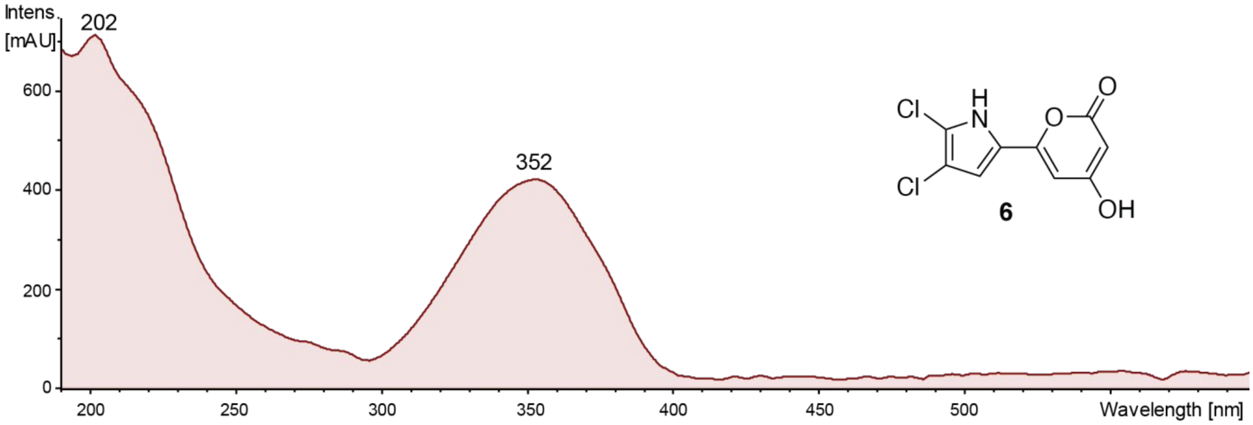

Fig. S4: UV-Vis absorbance profile of compound **6**.

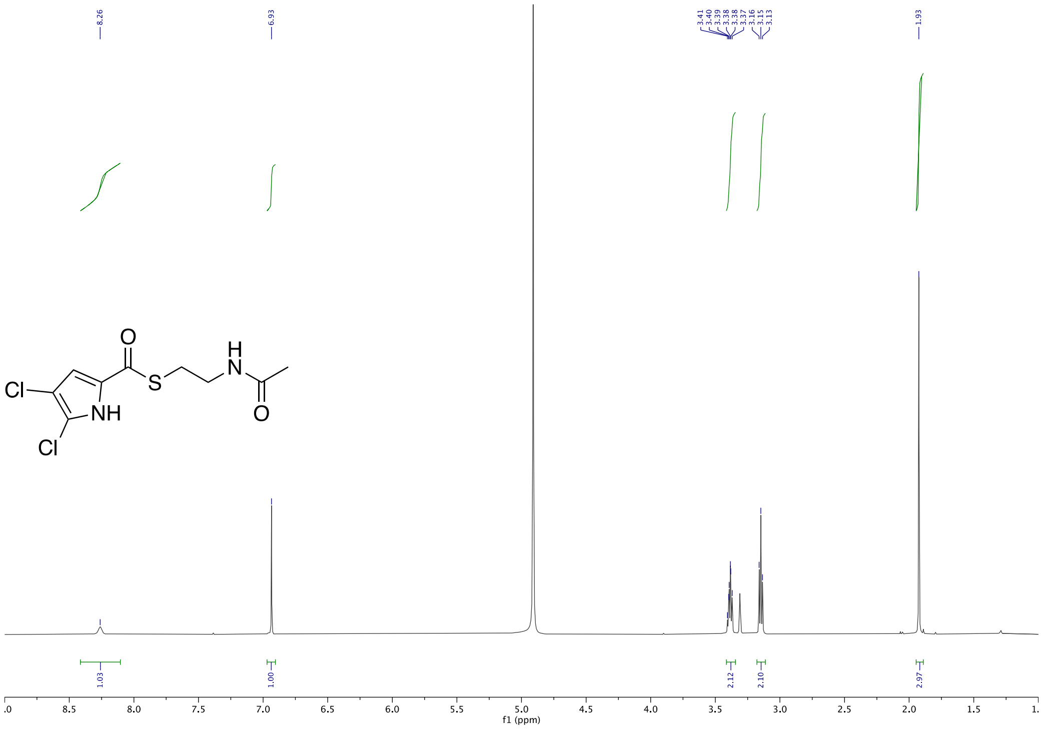

Fig. S5: ^{1}H NMR (500 MHz, MeOD) of compound **8a.**

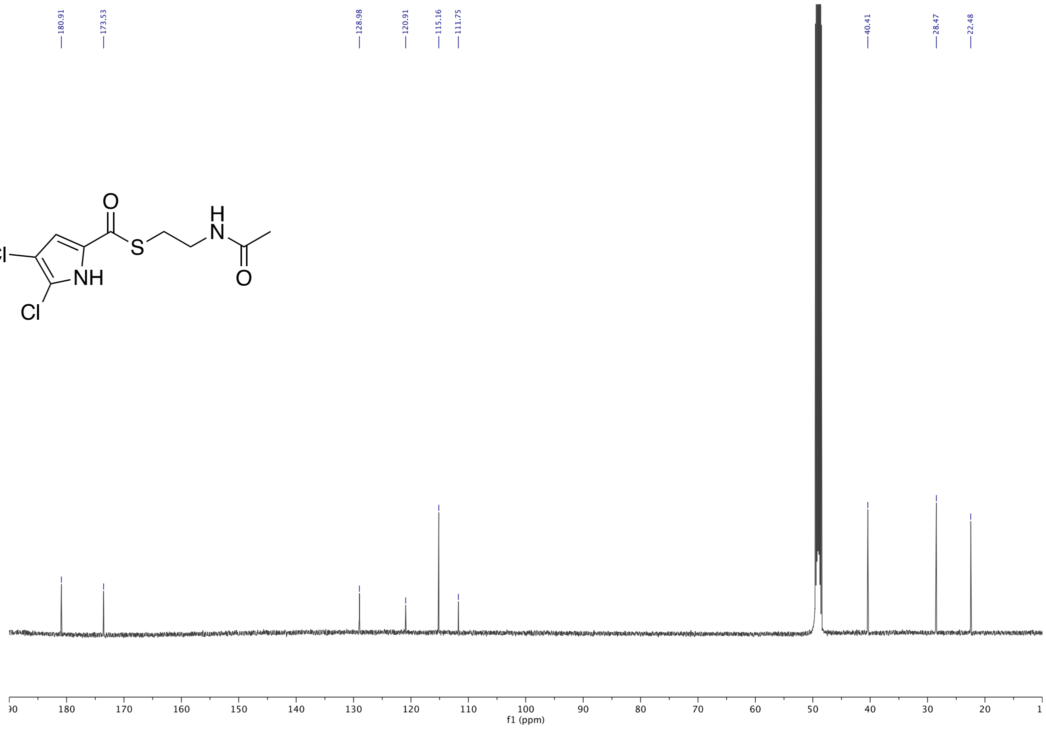

Fig. S6: ^{13}C NMR (126 MHz, MeOD) of compound **8a**.

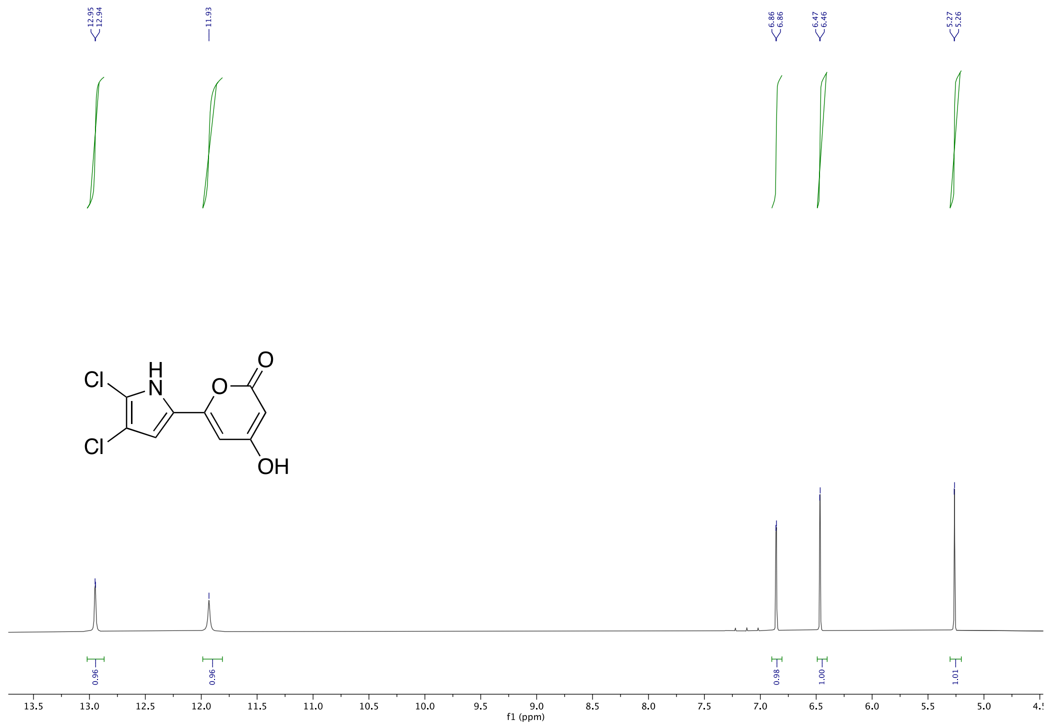

Fig. S7: ^{1}H NMR spectrum (500 MHz, DMSO-d_6) of compound **6**.

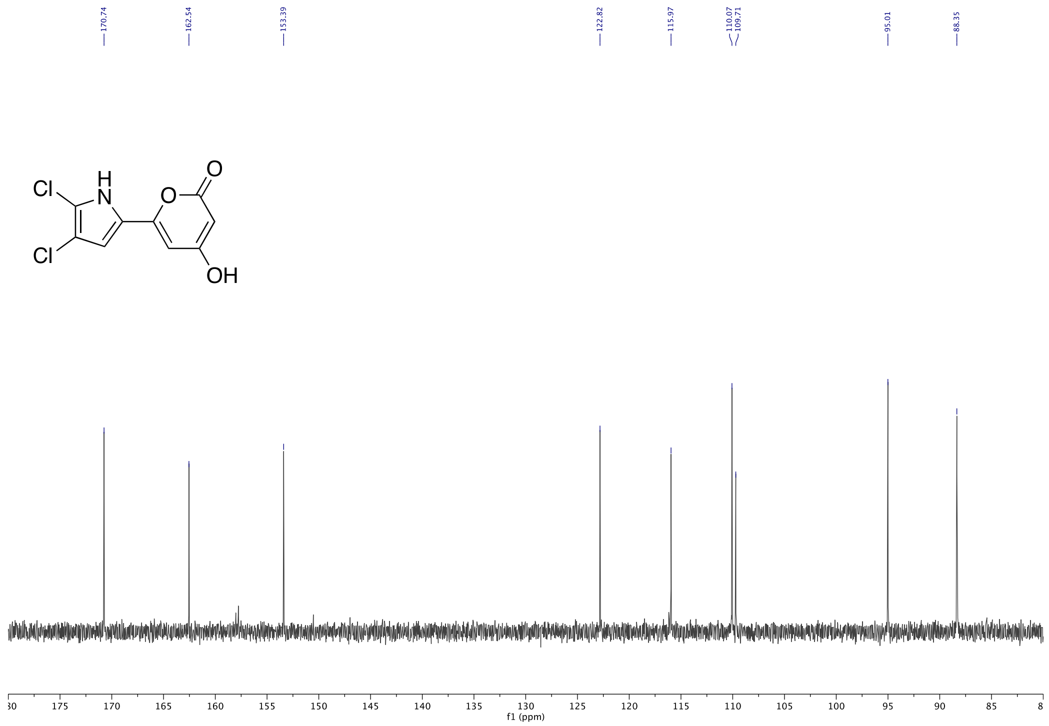

Fig. S8: ^{13}C NMR spectrum (126 MHz, DMSO-d_6) of compound **6**.

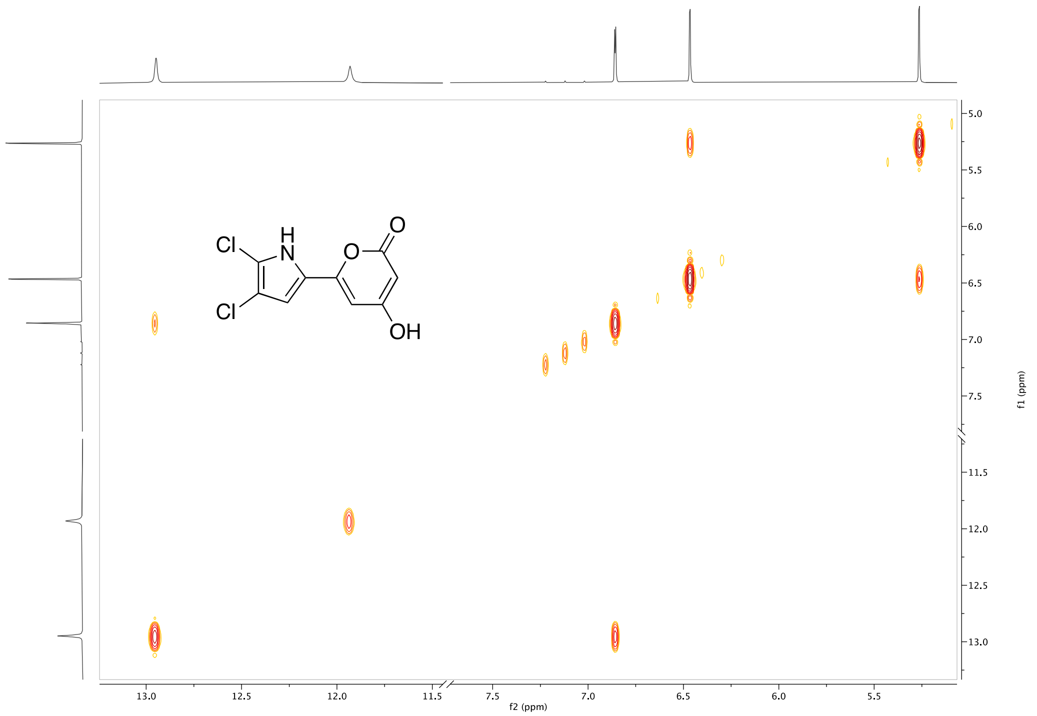

Fig. S9: ^{1}H -^{1}H COSY spectrum compound **6** in DMSO-d_6.

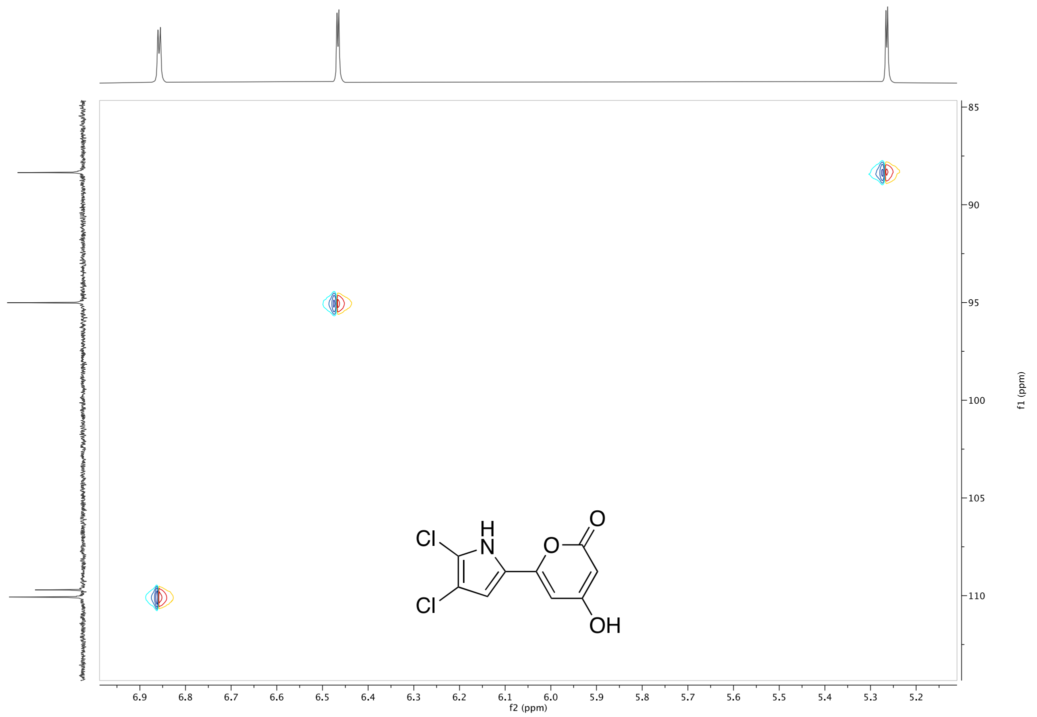

Fig. S10: ^{1}H -^{13}C HSQC spectrum compound **6** in DMSO-d_6.

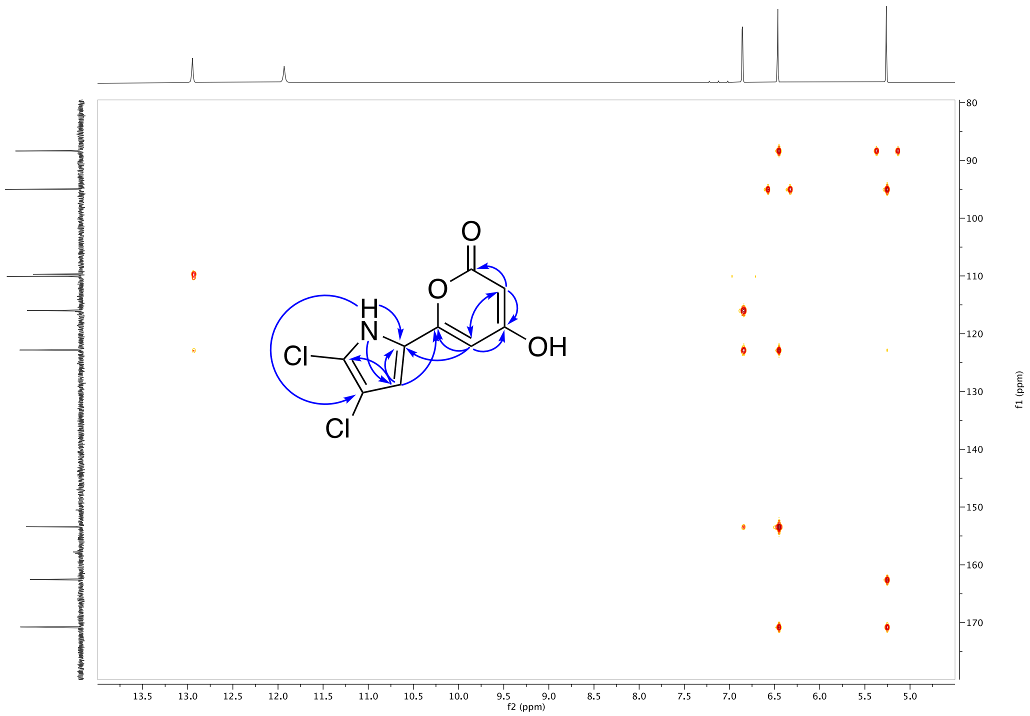

Fig. S11: ^{1}H-^{13}C HMBC spectrum compound **6** in DMSO-d_6.

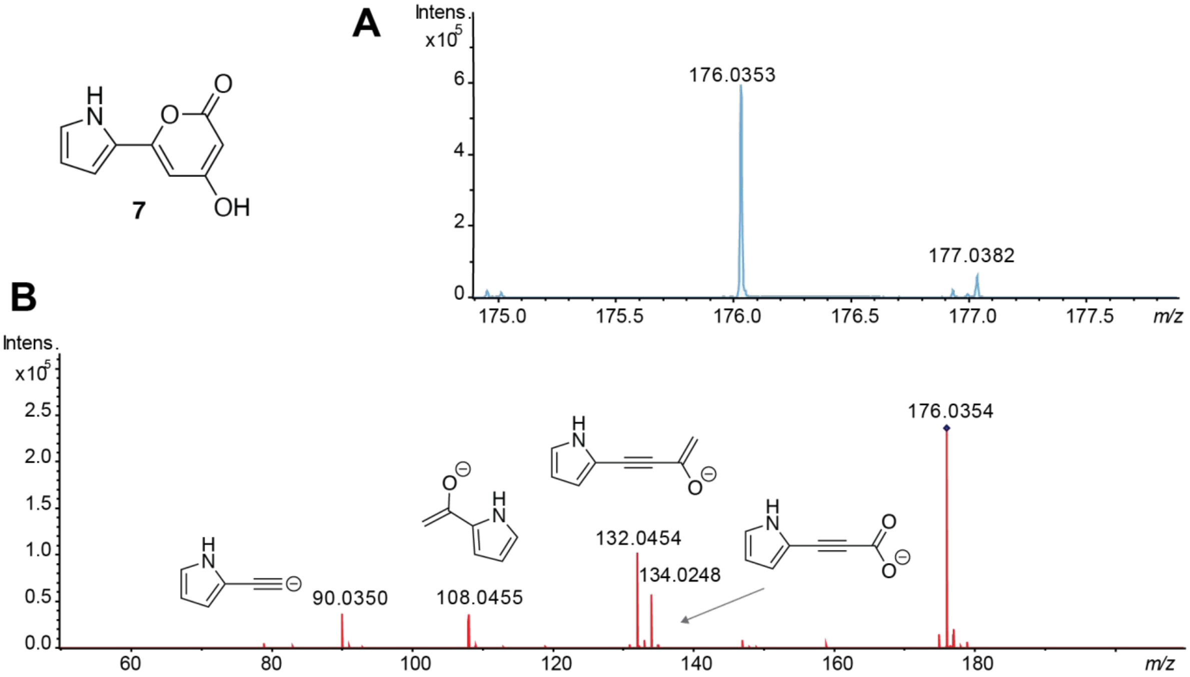

Fig. S12: MS^1 and MS^2 spectra of compound **7**. (A) HRMS (ESI) identified molecule ions corresponding to $[M-H]^-$ for compound **7** (*m/z* calculated for $C_9H_6NO_3$ 176.0353, found 176.0353). (B) MS^2 spectra of compound **7** with rationalized structural annotations of fragment ions. Note the similarity in fragmentation pattern for **7** and **6** (Fig. S3).

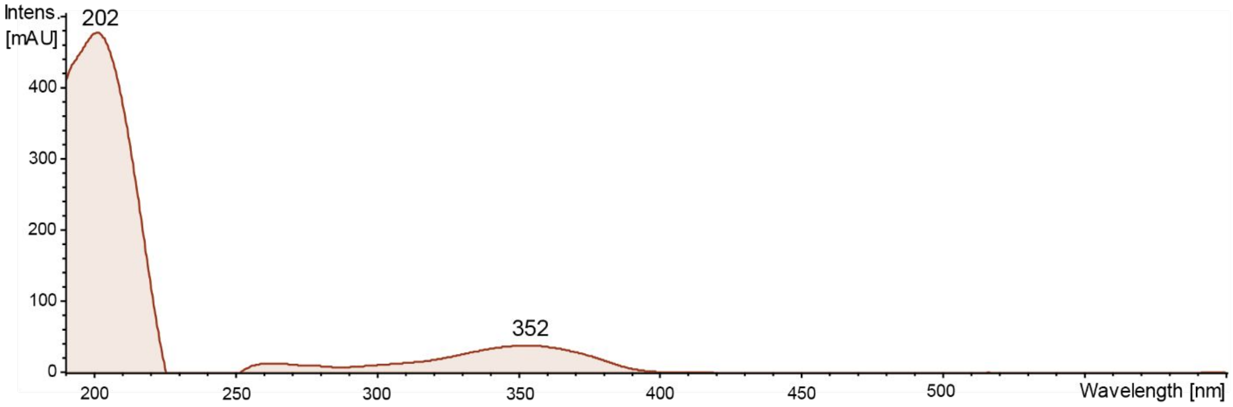

Fig. S13: UV absorbance profile of compound **7**. Note the similarity in UV-Vis absorbance spectra for **7** and **6** (Fig. S4).

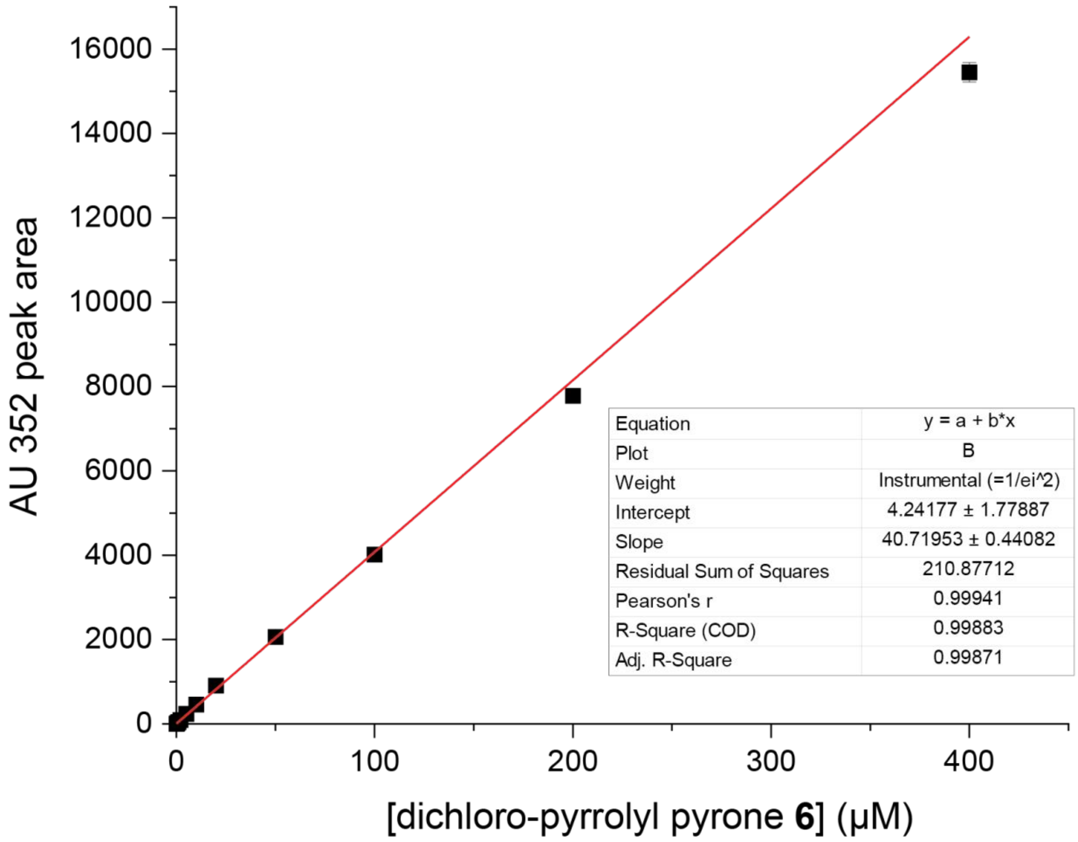

Fig. S14: Standard curve between area under the UV-Vis absorbance chromatogram recorded at 352 nm and concentration of compound **6**. This standard curve was used to quantify the concentration of **6** and **7** produced in assays with PKSs. Note that molecule **7** also possesses an absorbance maximum at 352 nm (Fig. S13).

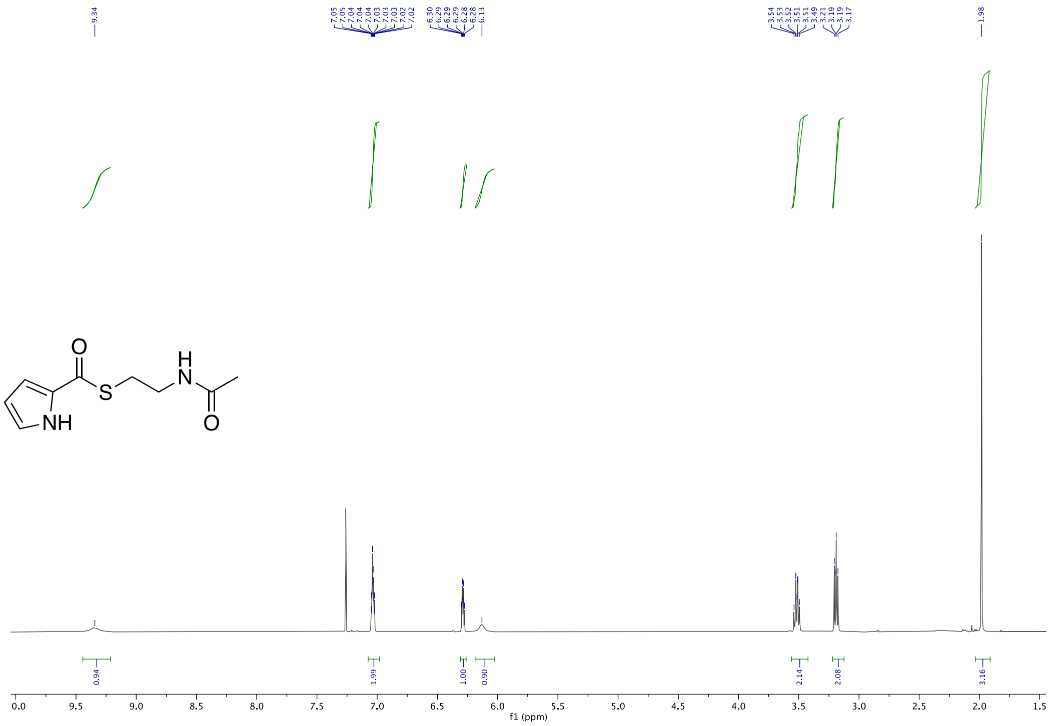

Fig. S15: ^{1}H NMR spectrum (400 MHz, $CDCl_3$) of compound **8b**.

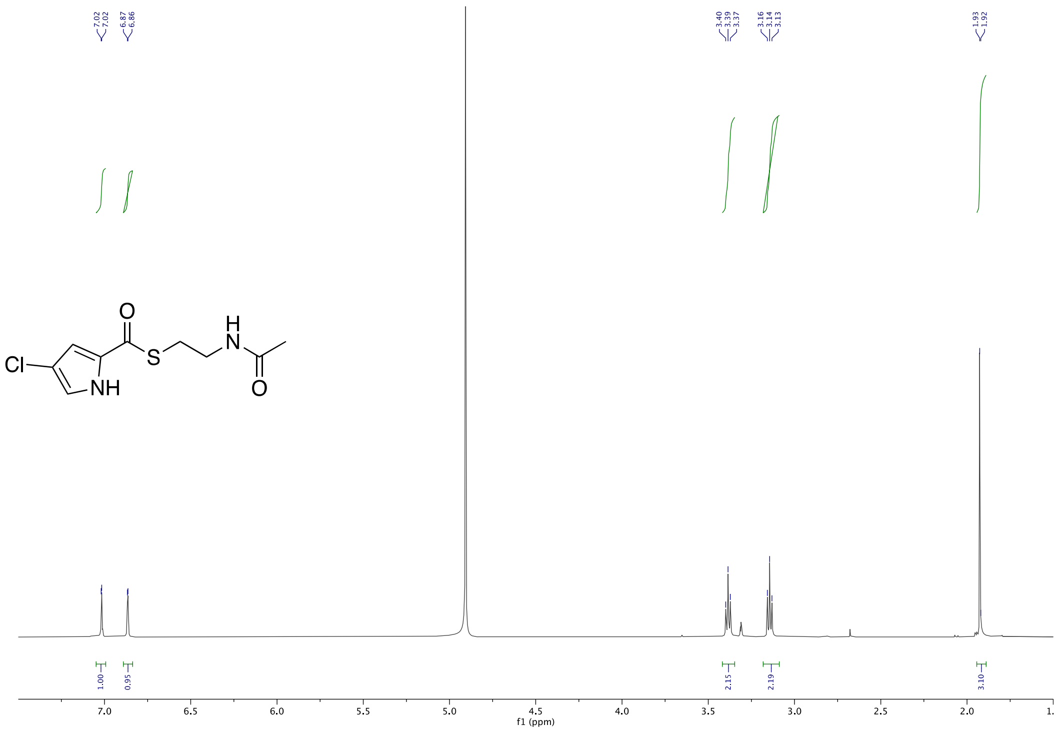

Fig. S16: ^{1}H NMR spectrum (500 MHz, MeOD) of compound **8c**.

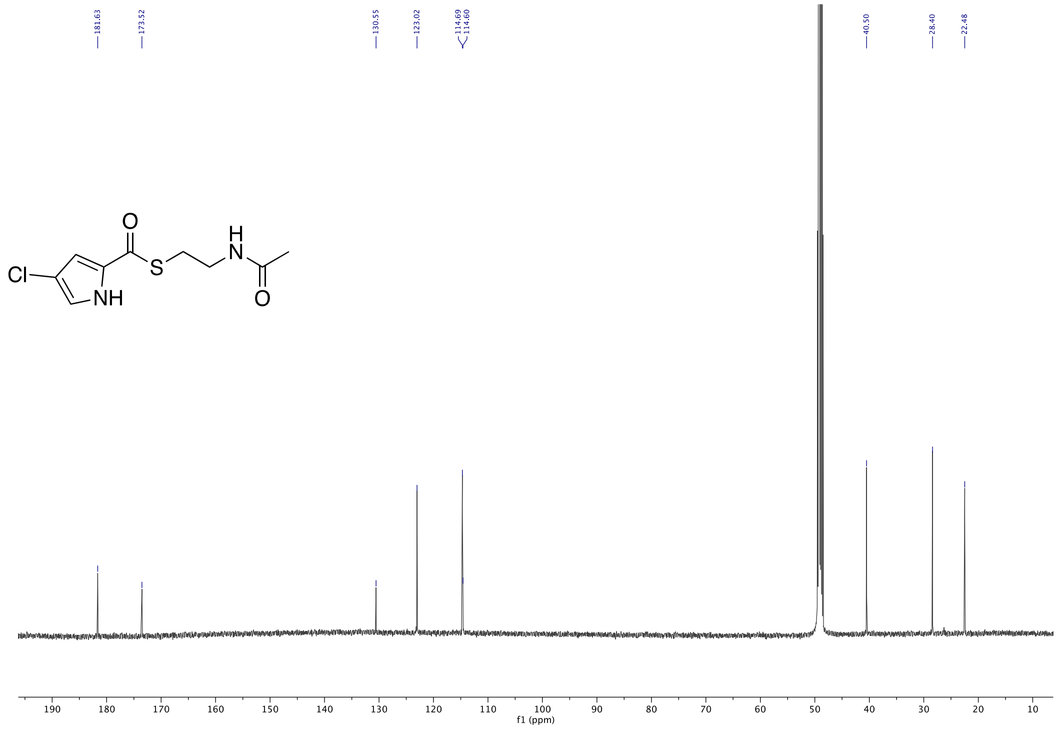

Fig. S17: ^{13}C NMR spectrum (126 MHz, MeOD) of compound **8c**.

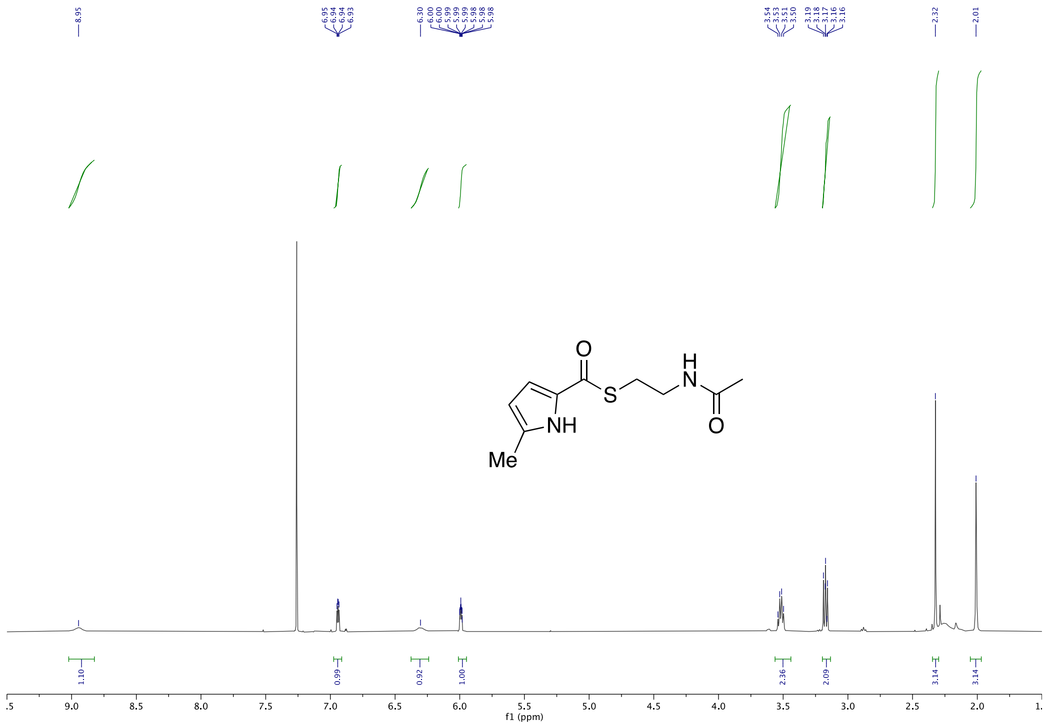

Fig. S18: ^{1}H NMR spectrum (400 MHz, $CDCl_3$) of compound **8d**.

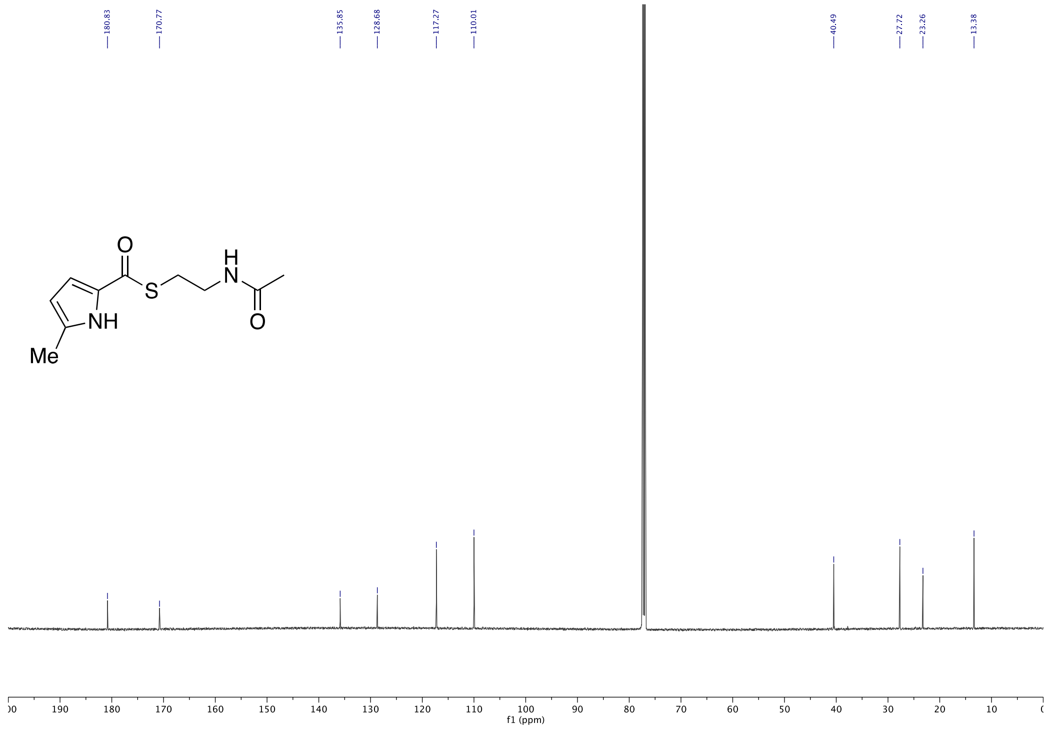

Fig. S19: ^{13}C NMR spectrum (126 MHz, $CDCl_3$) of compound **8d**.

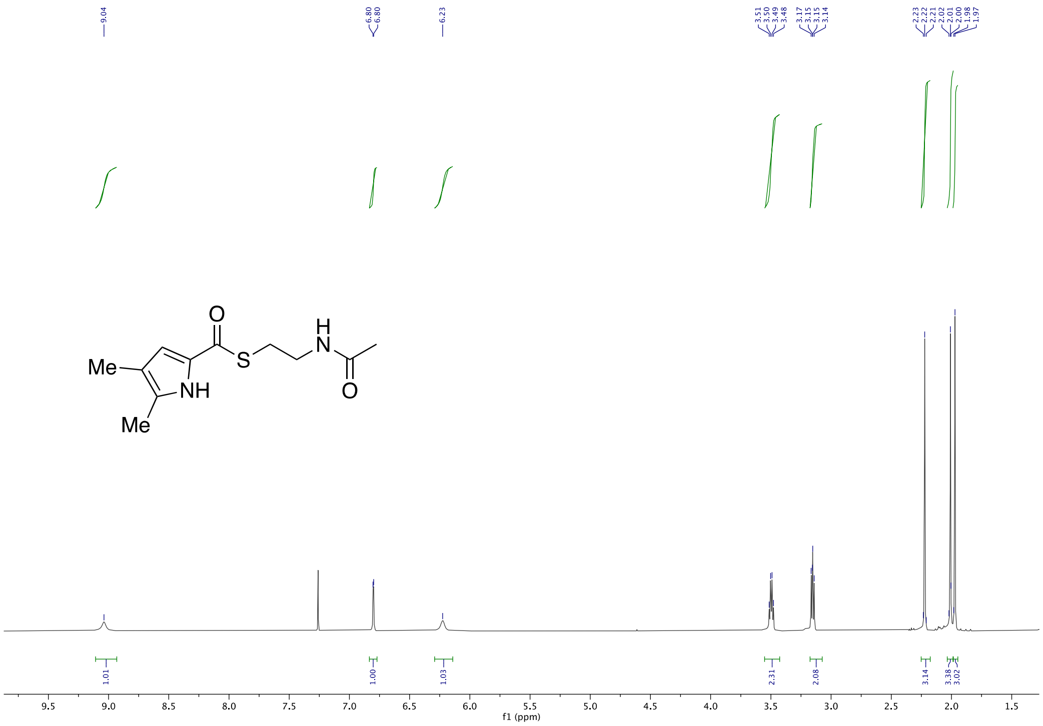

Fig. S20: ^{1}H NMR spectrum (500 MHz, $CDCl_3$) of compound **8e**.

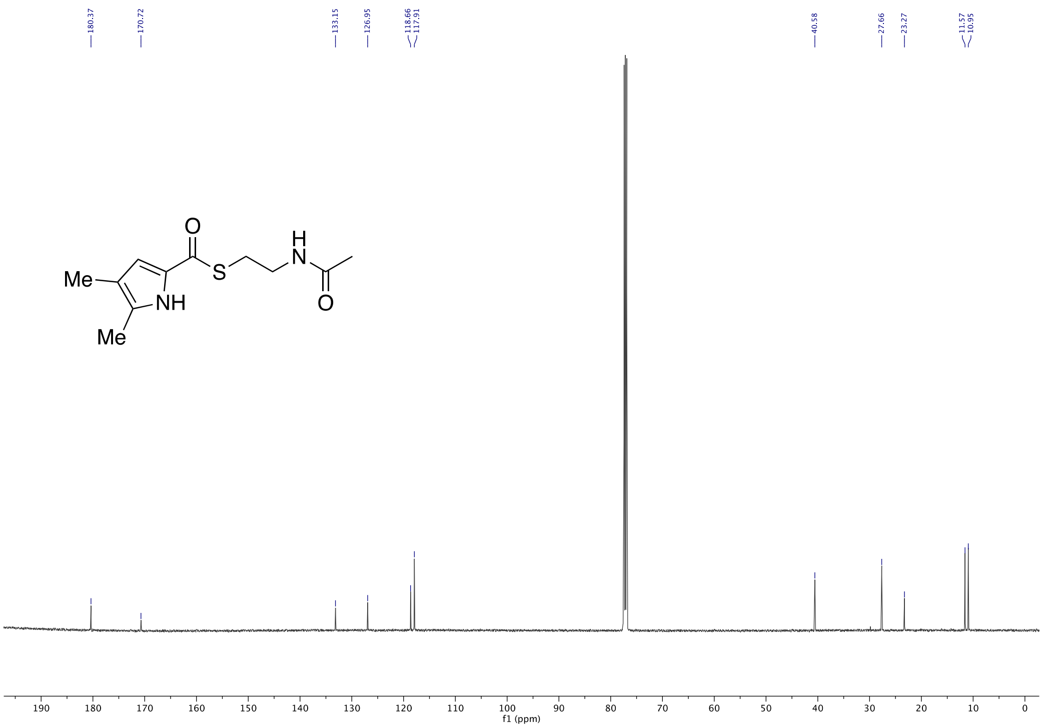

Fig. S21: ^{13}C NMR spectrum (126 MHz, $CDCl_3$) of compound **8e**.

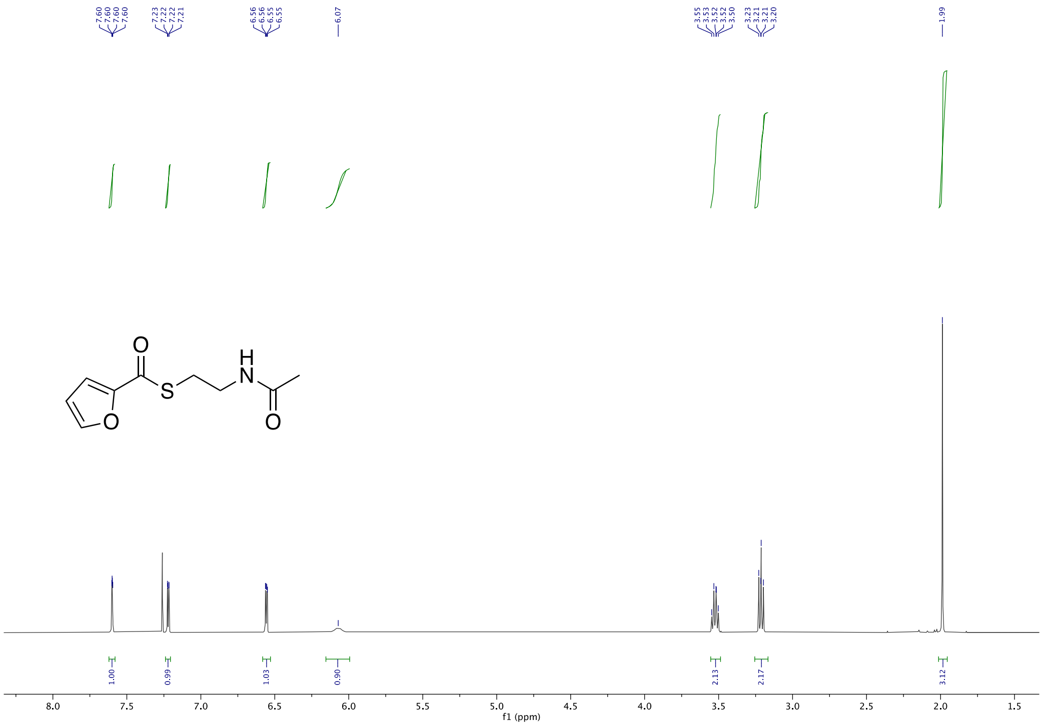

Fig. S22: ^{1}H NMR spectrum (400 MHz, $CDCl_3$) of compound **8f**.

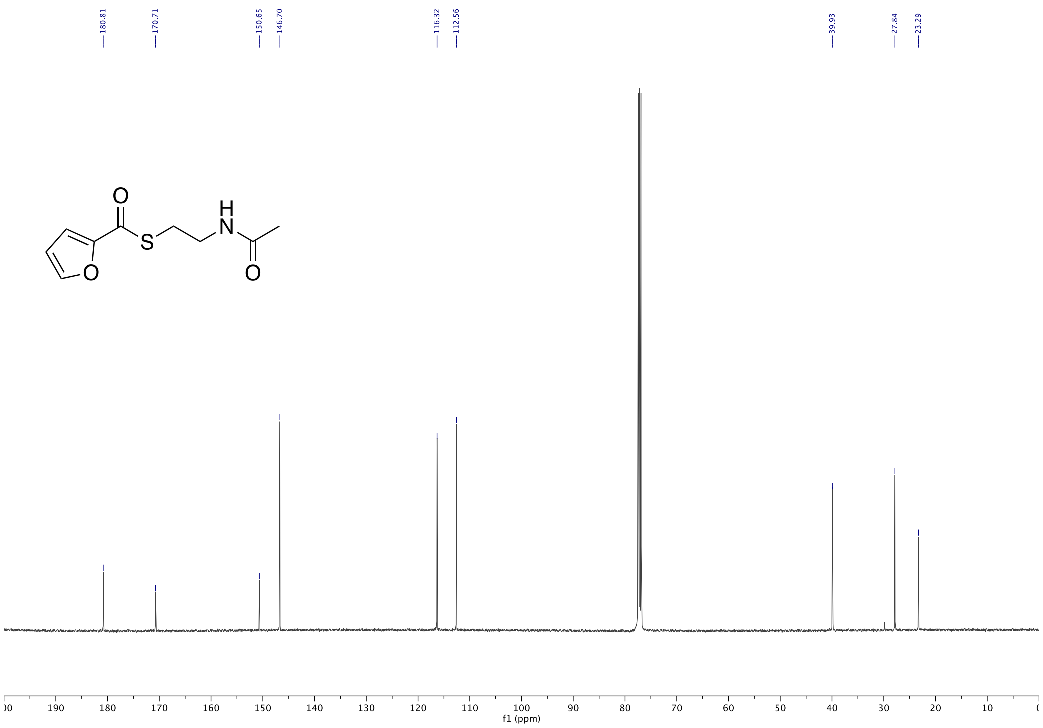

Fig. S23: ^{13}C NMR spectrum (126 MHz, $CDCl_3$) of compound **8f**.

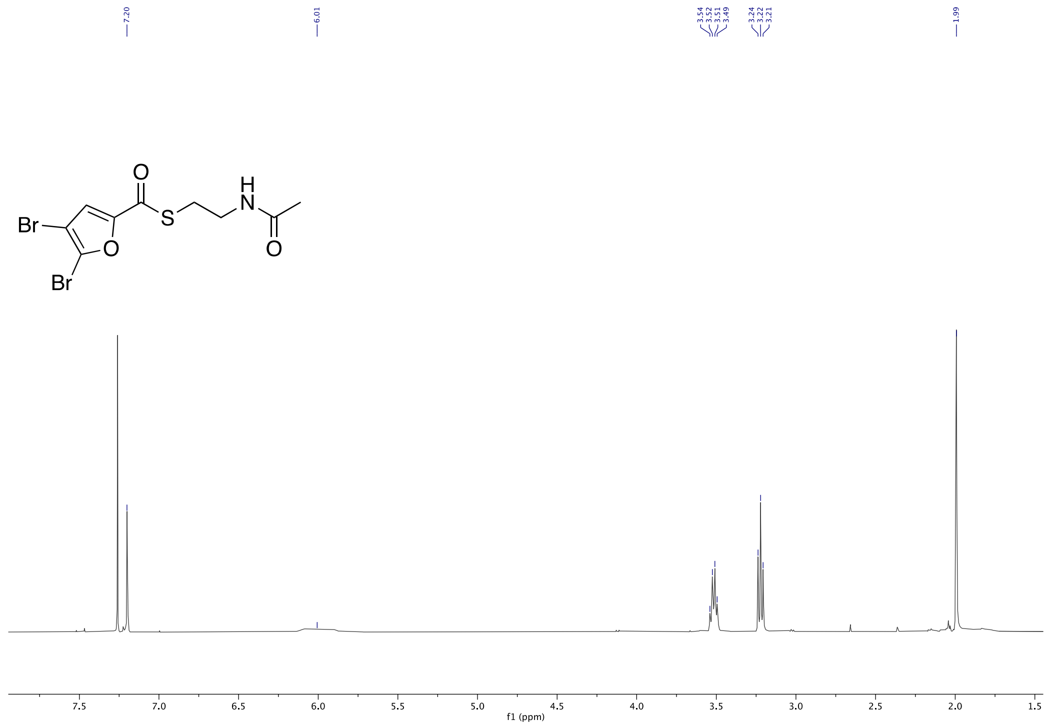

Fig. S24: ^{1}H NMR spectrum (400 MHz, $CDCl_3$) of compound **8g**.

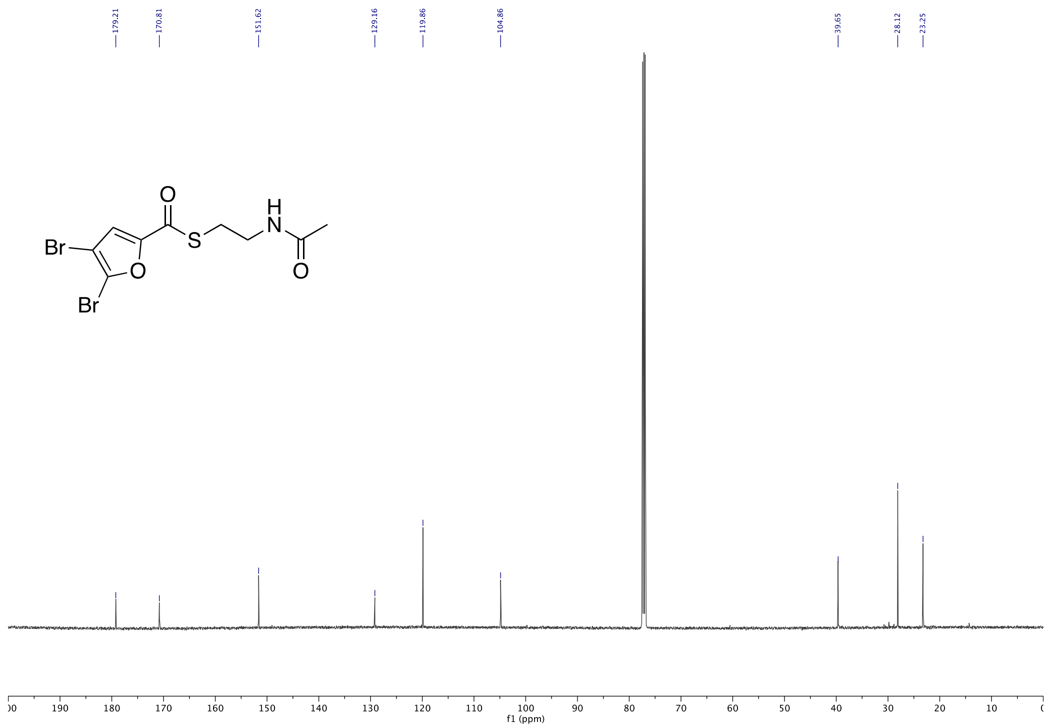

Fig. S25: ^{13}C NMR spectrum (126 MHz, $CDCl_3$) of compound **8g**.

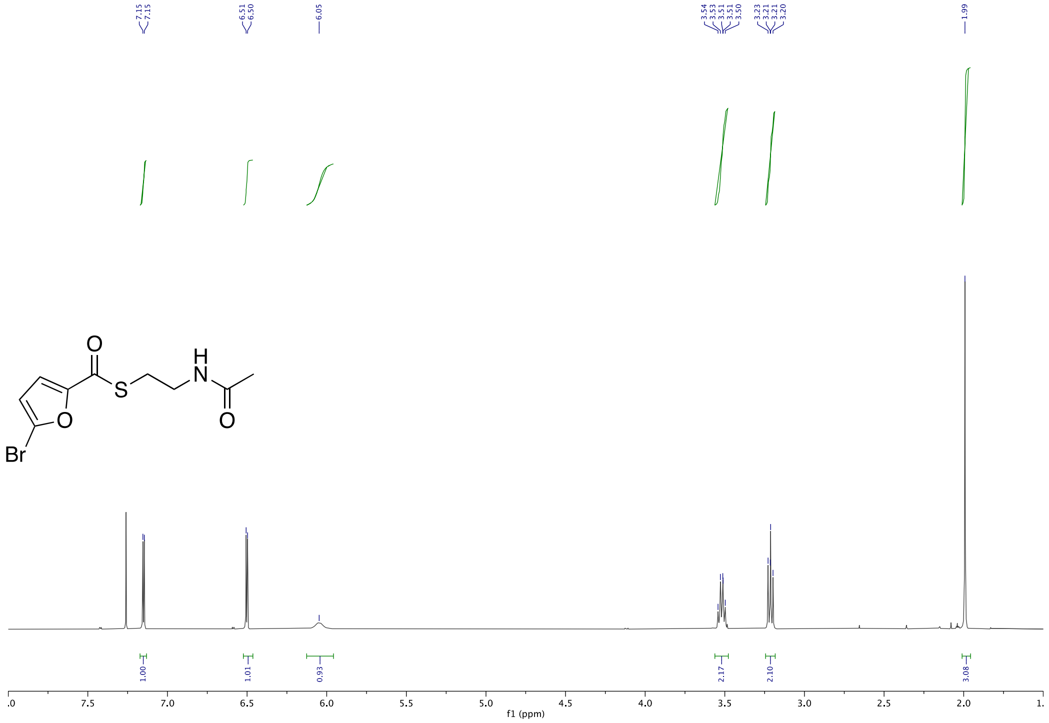

Fig. S26: ^{1}H NMR spectrum (400 MHz, $CDCl_3$) of compound **8h**.

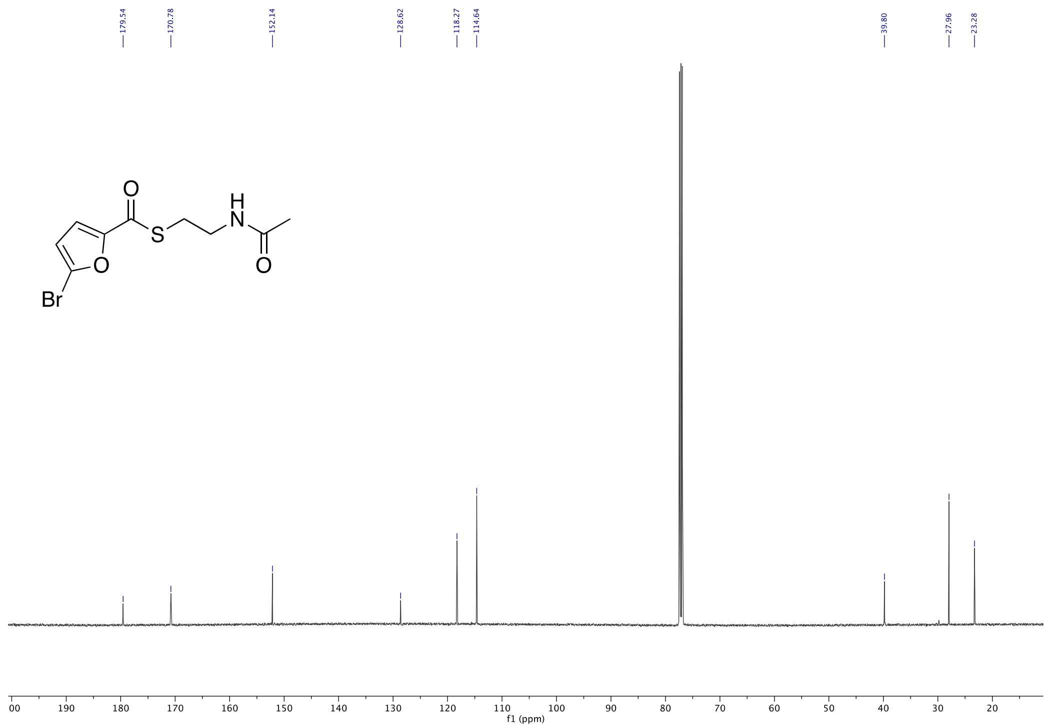

Fig. S27: ^{13}C NMR spectrum (126 MHz, $CDCl_3$) of compound **8h**.

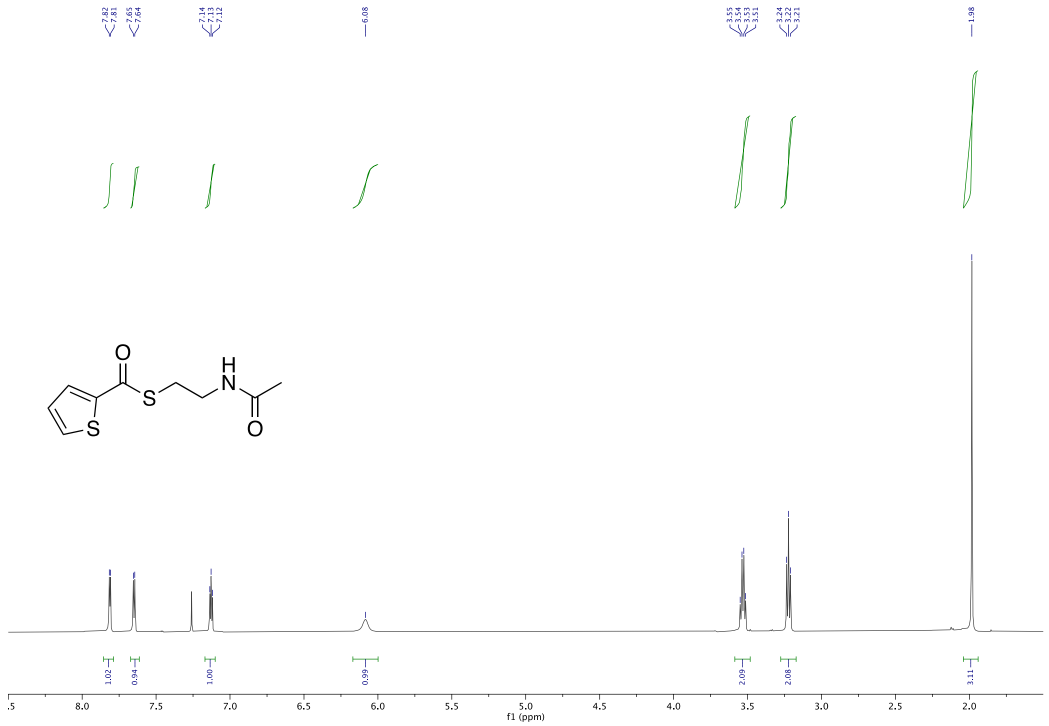

Fig. S28: ^{1}H NMR spectrum (500 MHz, $CDCl_3$) of compound **8i**.

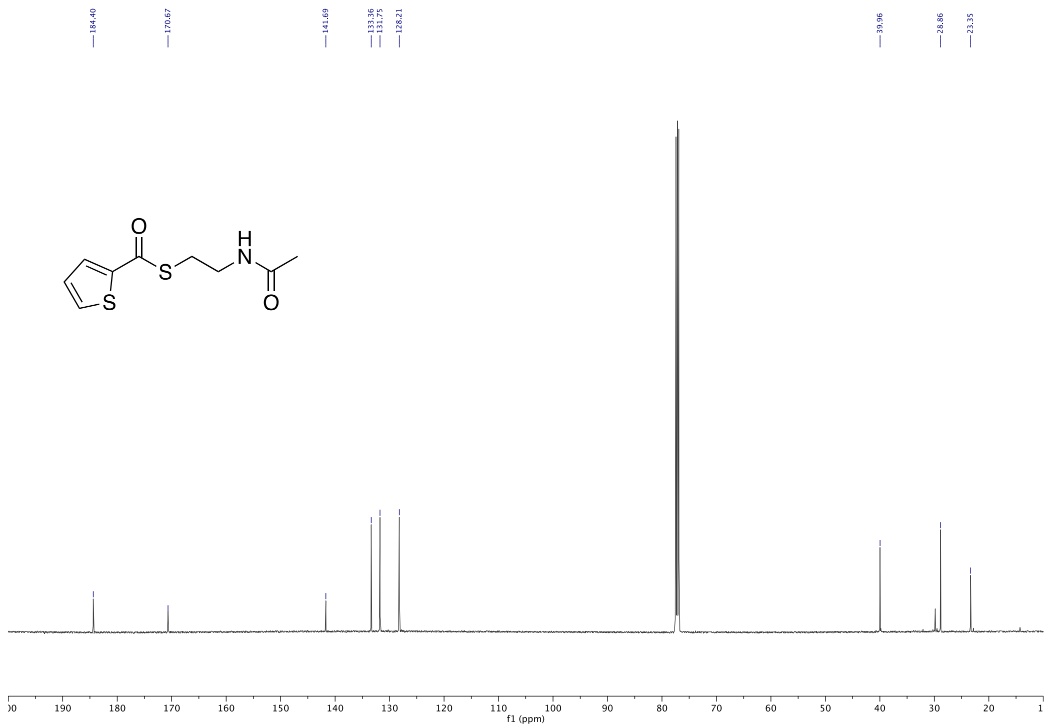

Fig. S29: ^{13}C NMR spectrum (126 MHz, $CDCl_3$) of compound **8i**.

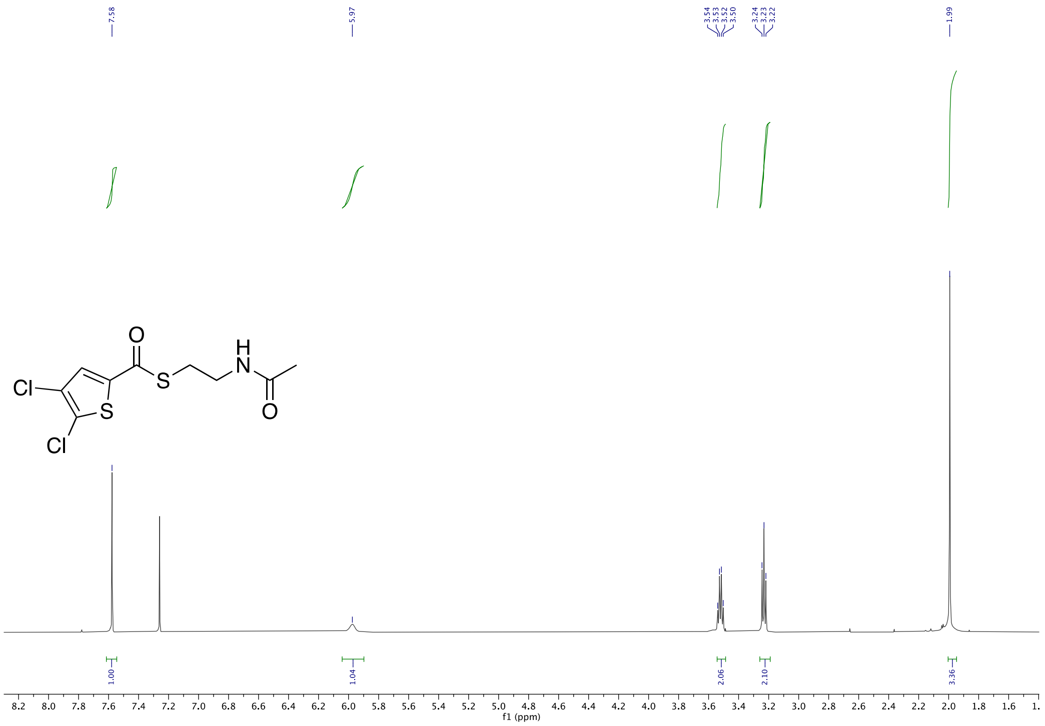

Fig. S30: ^{1}H NMR spectrum (500 MHz, $CDCl_3$) of compound **8j**.

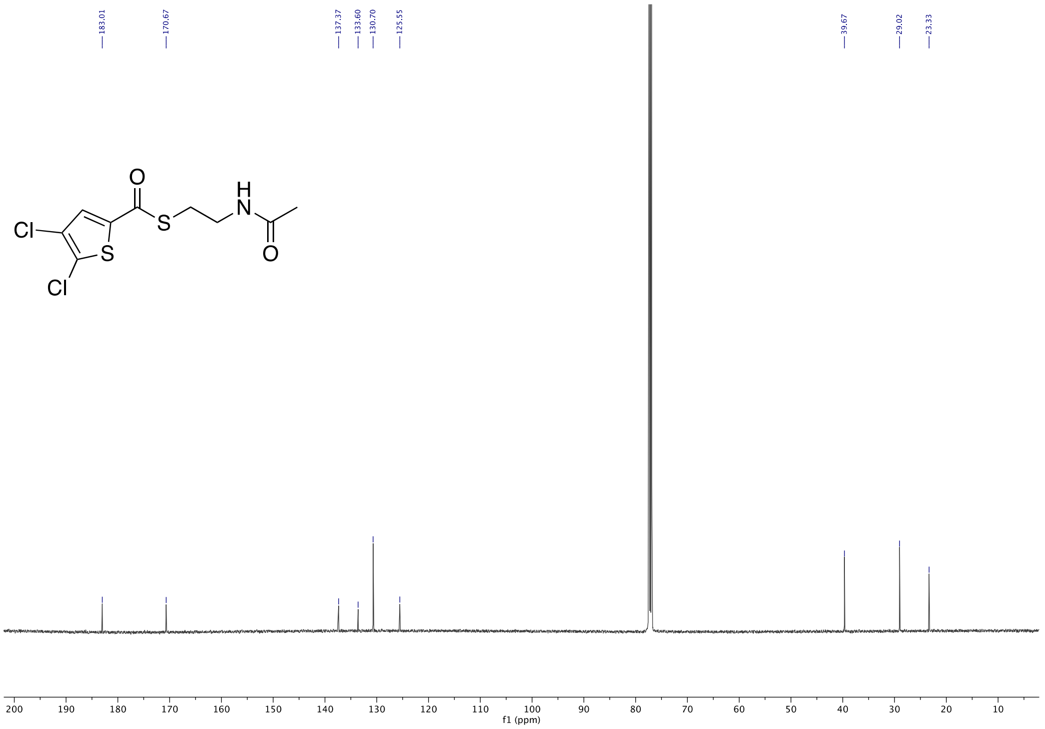

Fig. S31: ^{13}C NMR spectrum (126 MHz, $CDCl_3$) of compound **8j**.

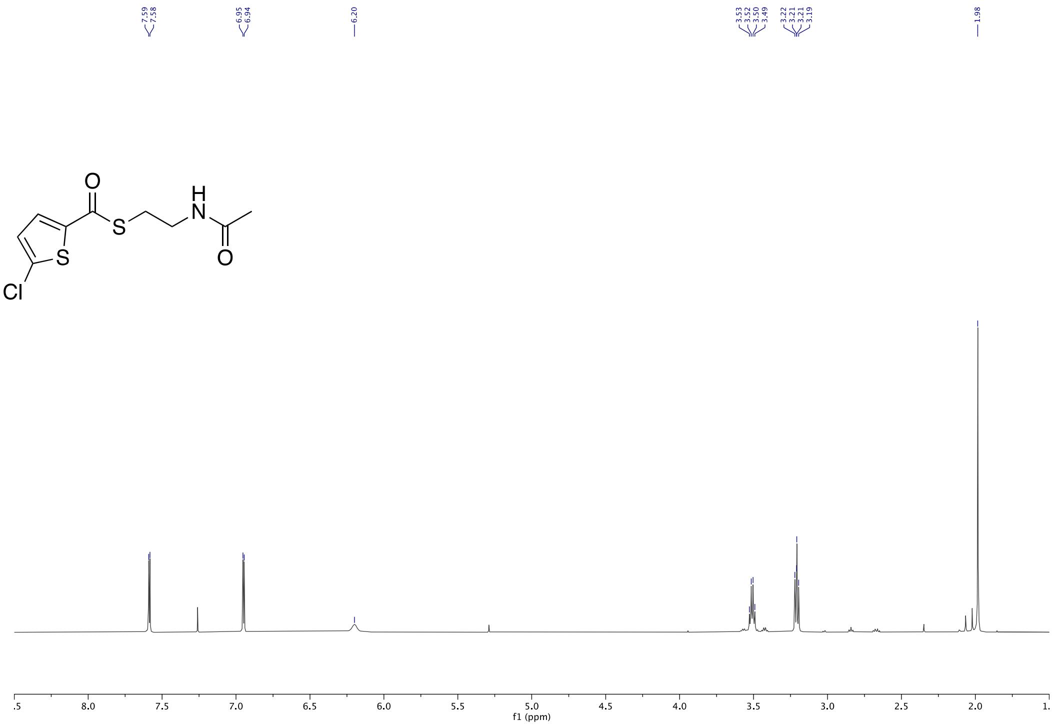

Fig. S32: ^{1}H NMR spectrum (500 MHz, $CDCl_3$) of compound **8k**.

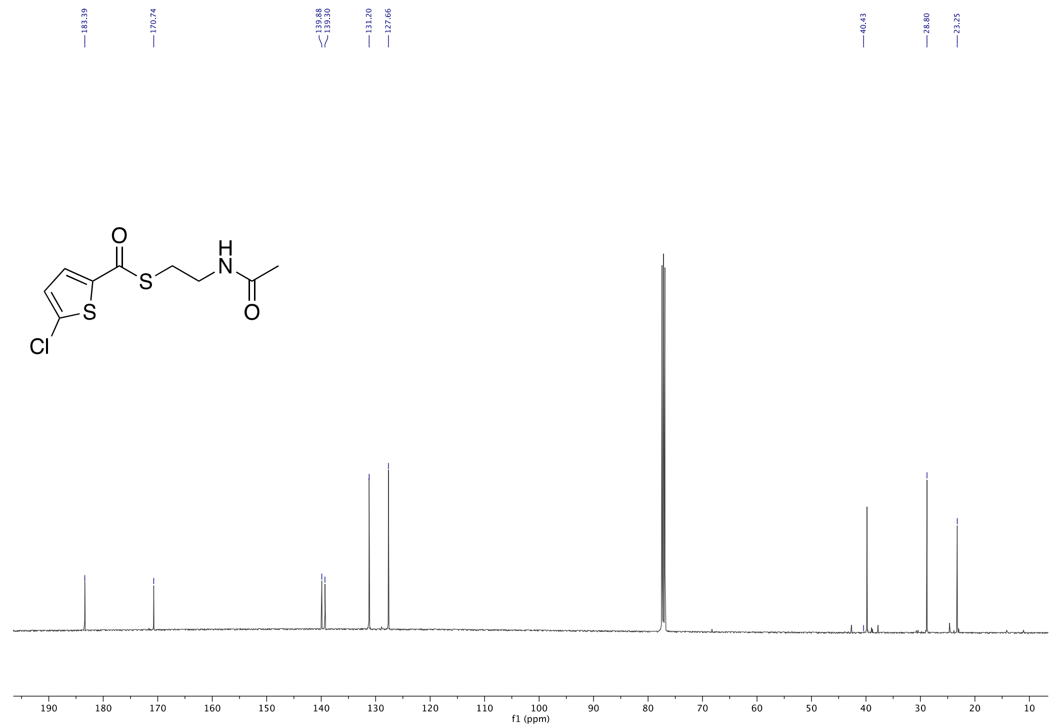

Fig. S33: ^{13}C NMR spectrum (126 MHz, $CDCl_3$) of compound **8k**.

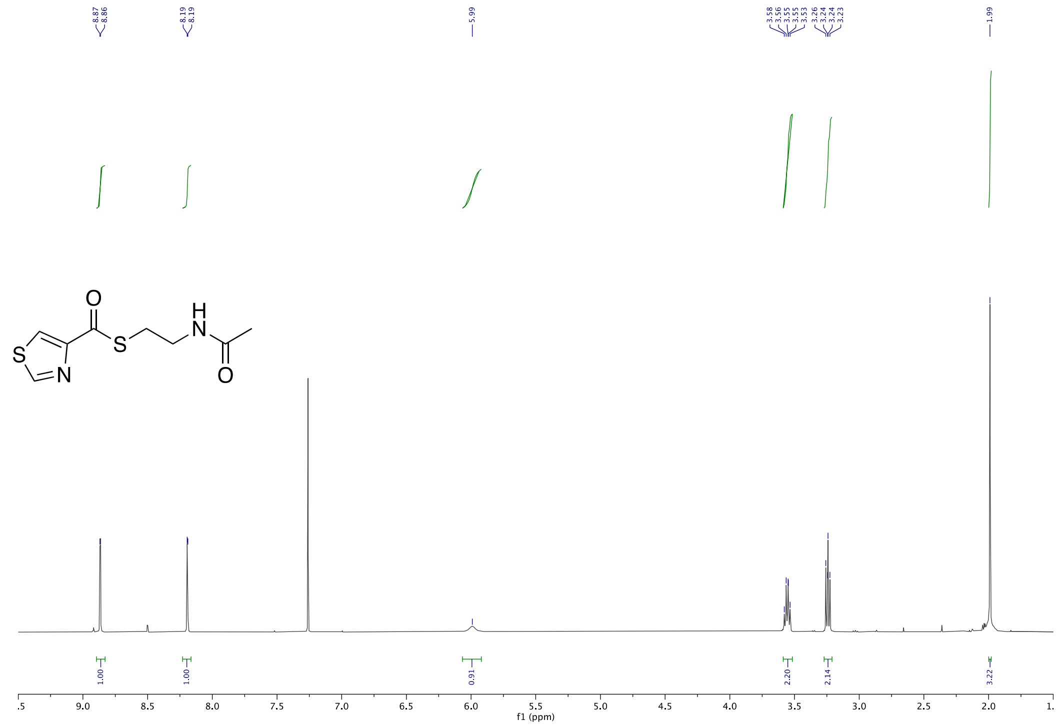

Fig. S34: ^{1}H NMR spectrum (400 MHz, $CDCl_3$) of compound **8l**.

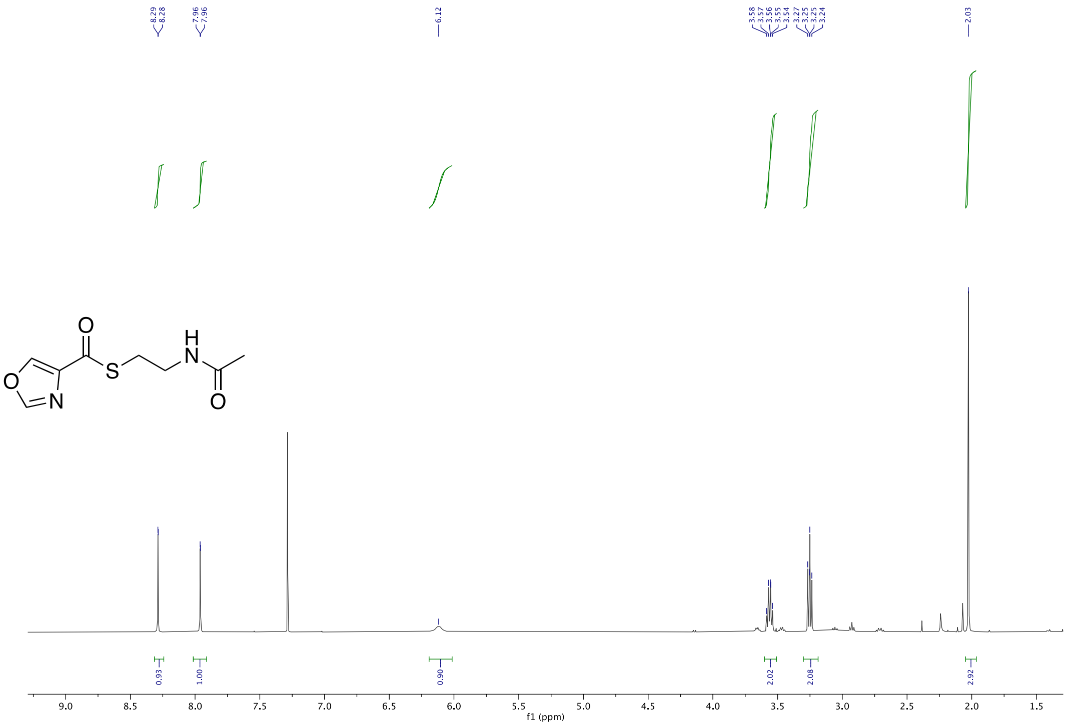

Fig. S35: ^{1}H NMR spectrum (400 MHz, $CDCl_3$) of compound **8m**.

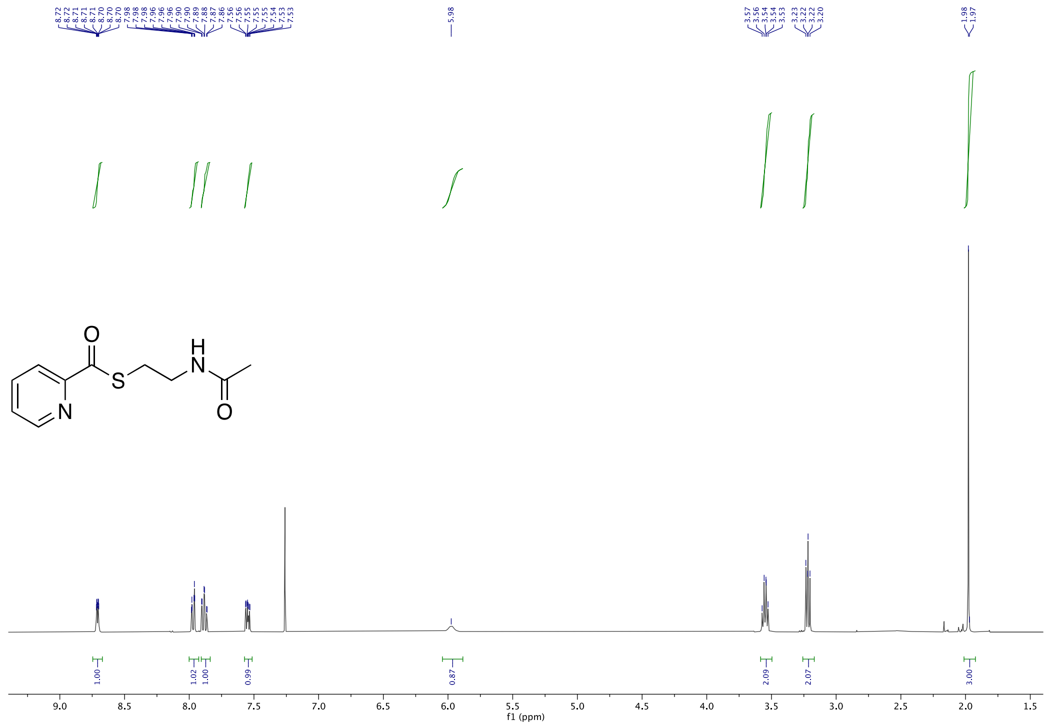

Fig. S36: ^{1}H NMR spectrum (400 MHz, $CDCl_3$) of compound **8n**.

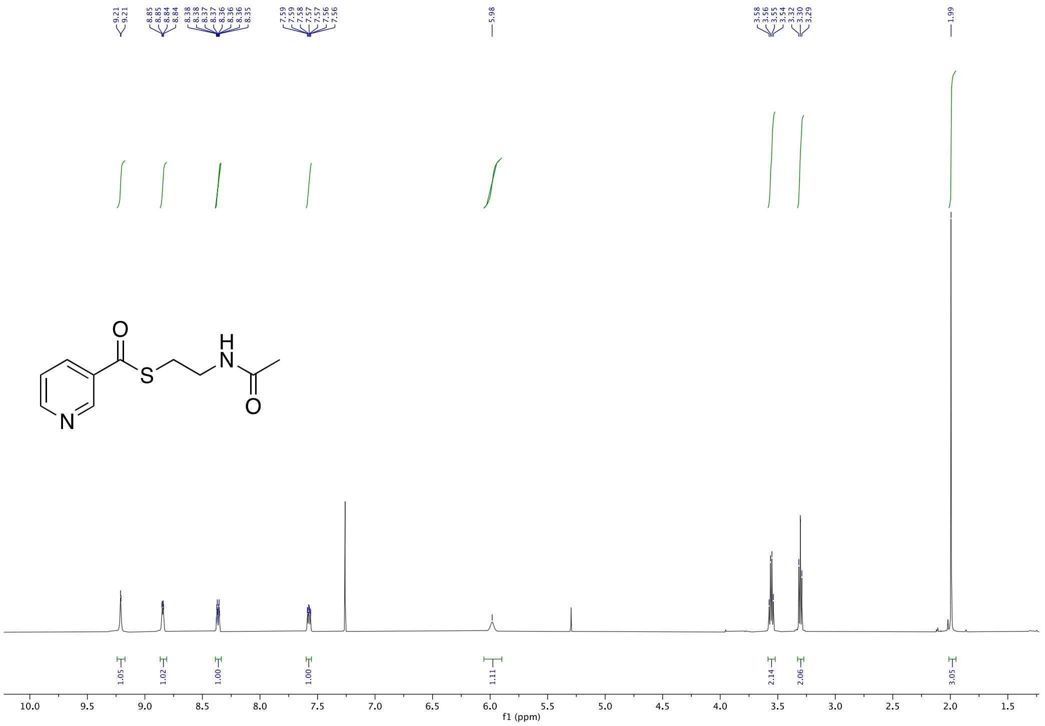

Fig. S37: ^{1}H NMR spectrum (500 MHz, $CDCl_3$) of compound **8o**.

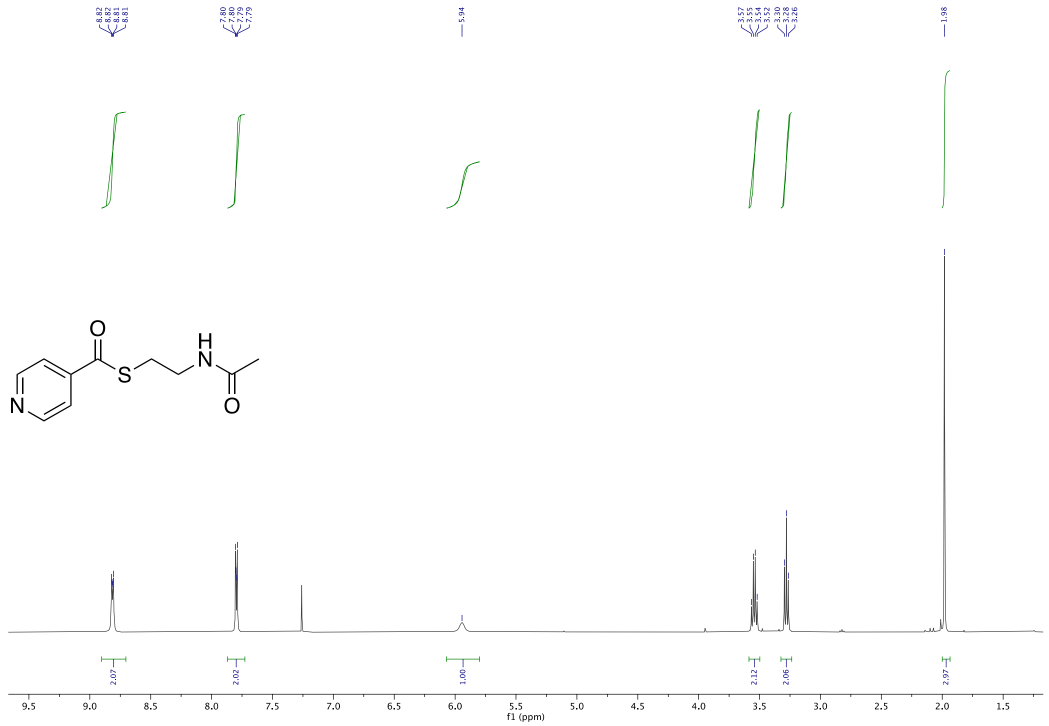

Fig. S38: ^{1}H NMR spectrum (400 MHz, $CDCl_3$) of compound **8p**.

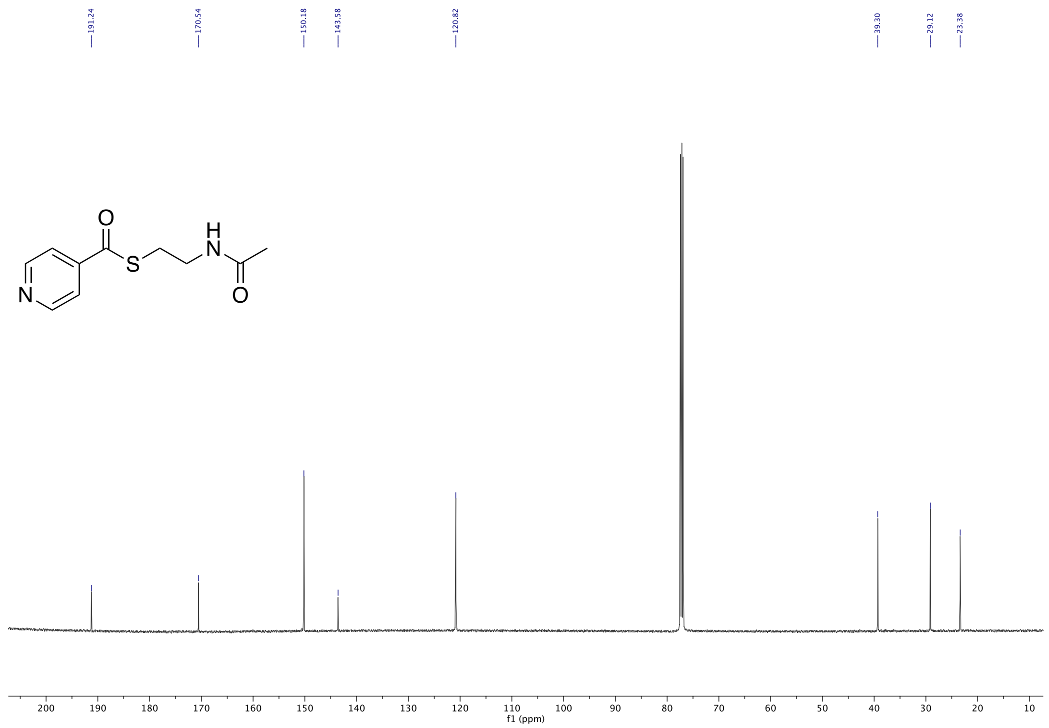

Fig. S39: ^{13}C NMR spectrum (126 MHz, $CDCl_3$) of compound **8p**.

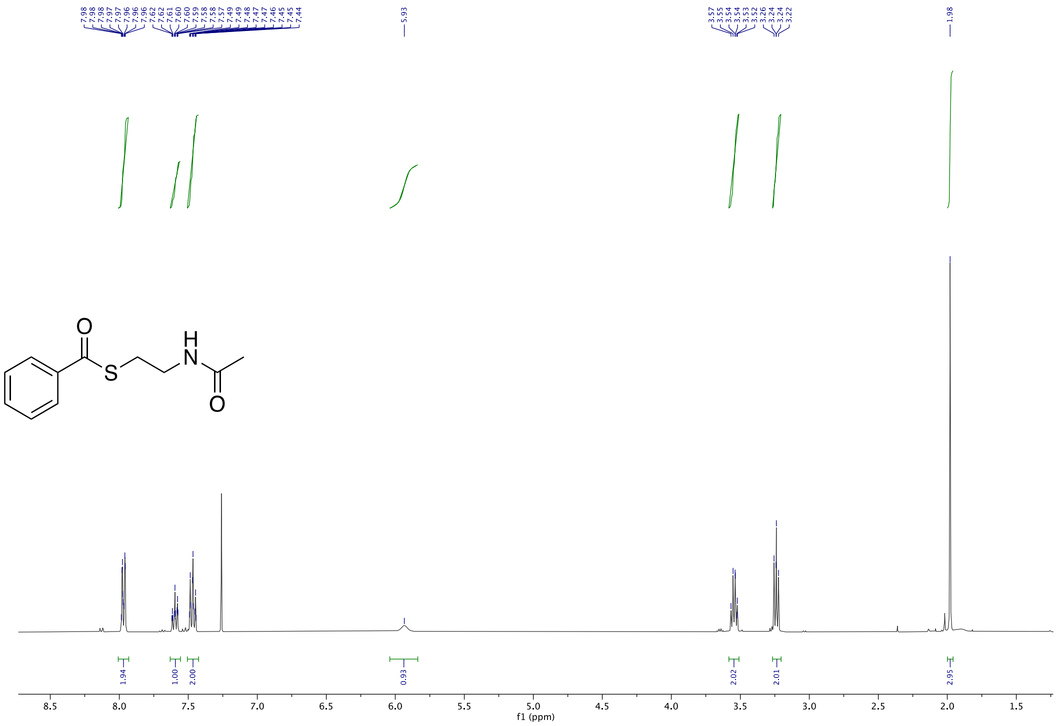

Fig. S40: ^{1}H NMR spectrum (400 MHz, $CDCl_3$) of compound **8q**.

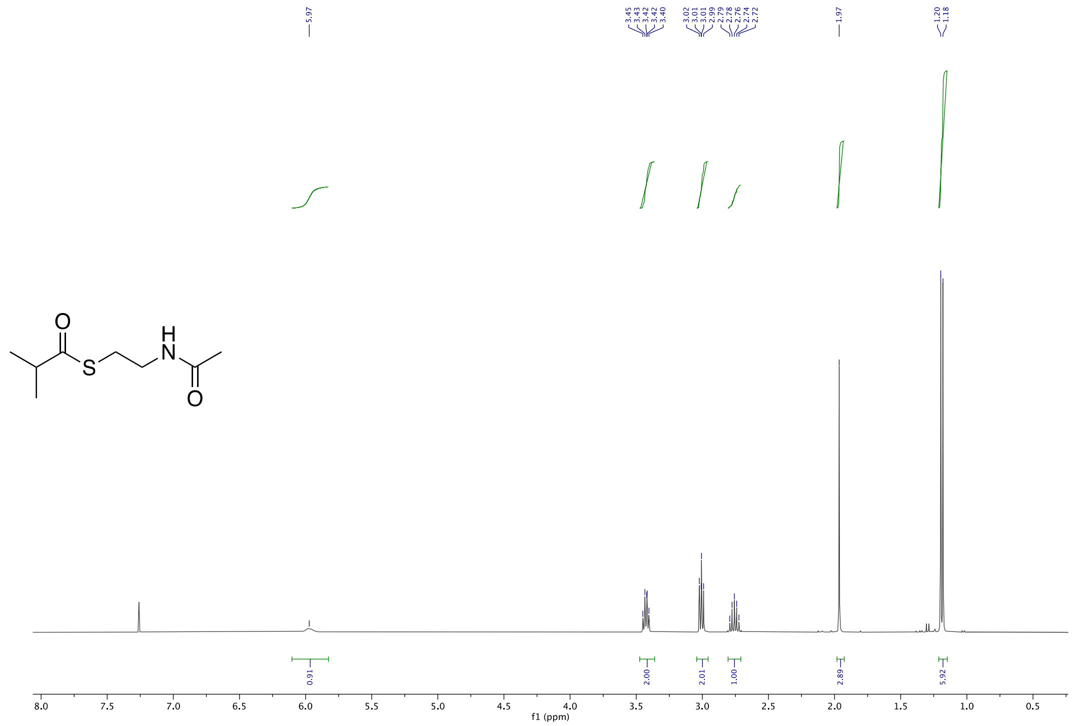

Fig. S41: ^{1}H NMR spectrum (400 MHz, $CDCl_3$) of compound **8r**.

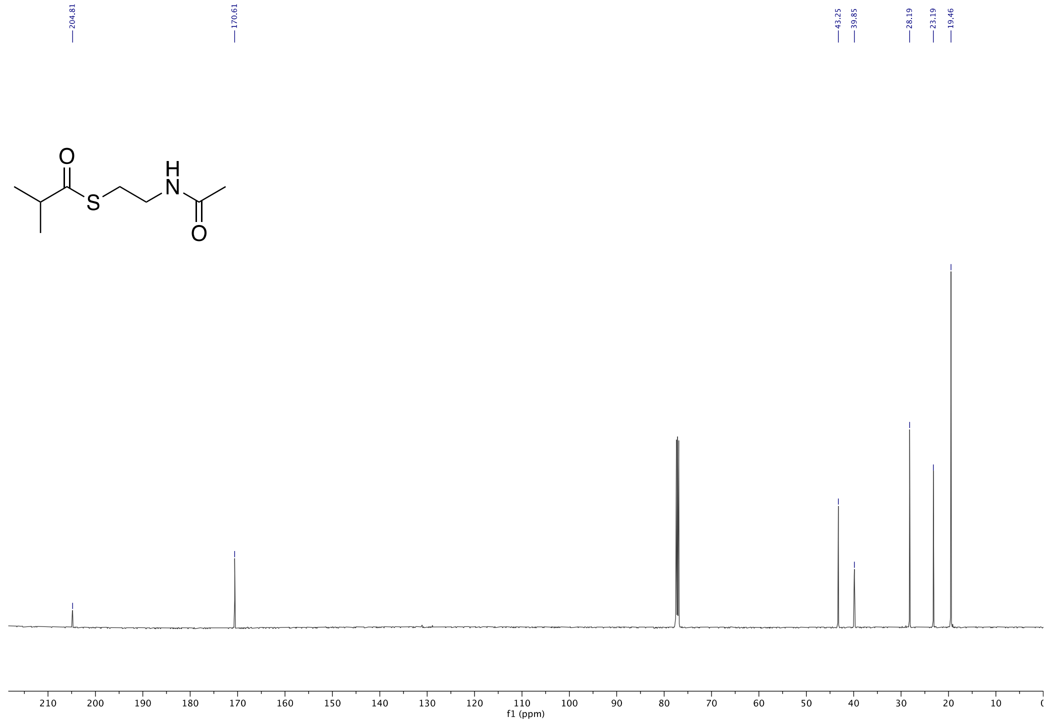

Fig. S42: ^{13}C NMR spectrum (126 MHz, $CDCl_3$) of compound **8r**.

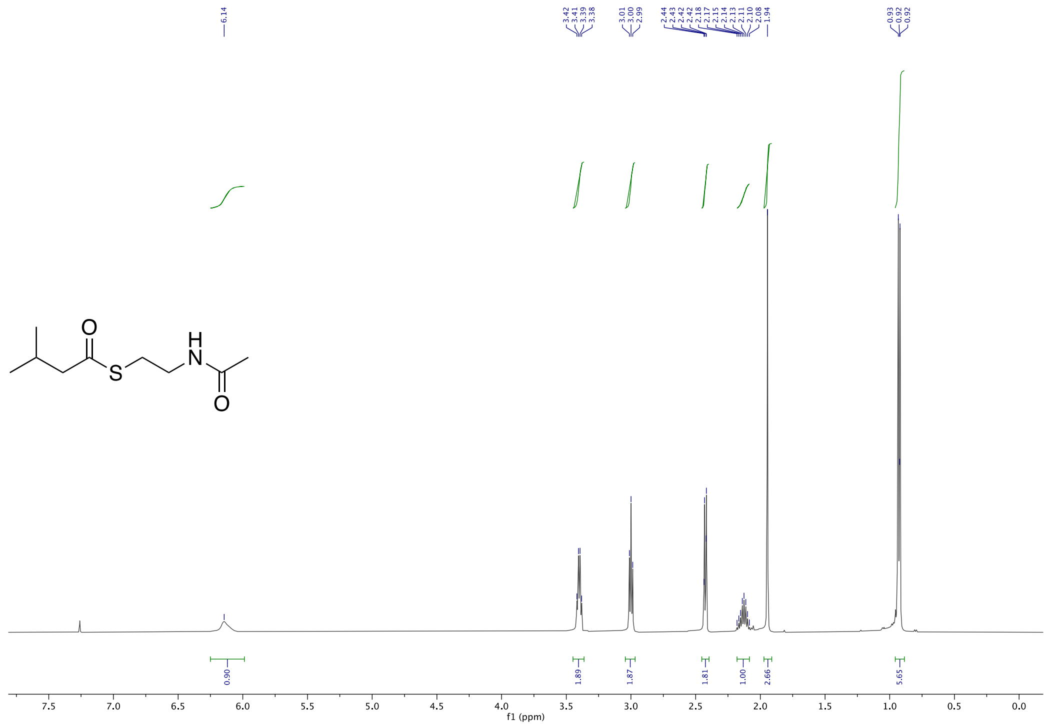

Fig. S43: ^{1}H NMR spectrum (500 MHz, $CDCl_3$) of compound **8s**.

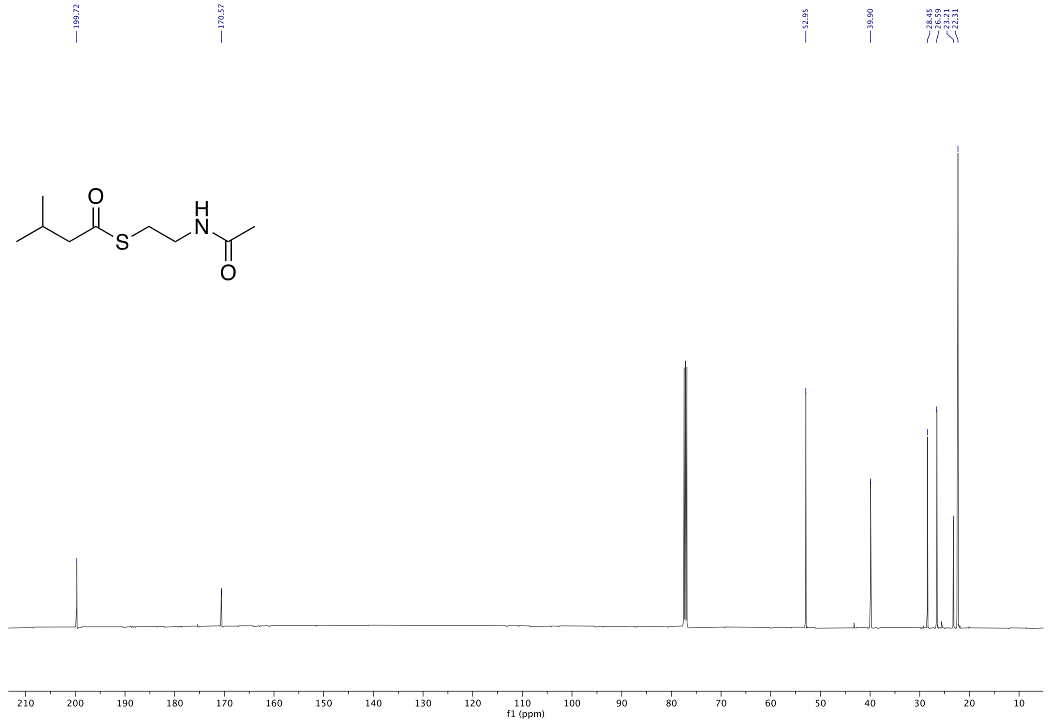

Fig. S44: ^{13}C NMR spectrum (126 MHz, $CDCl_3$) of compound **8s**.

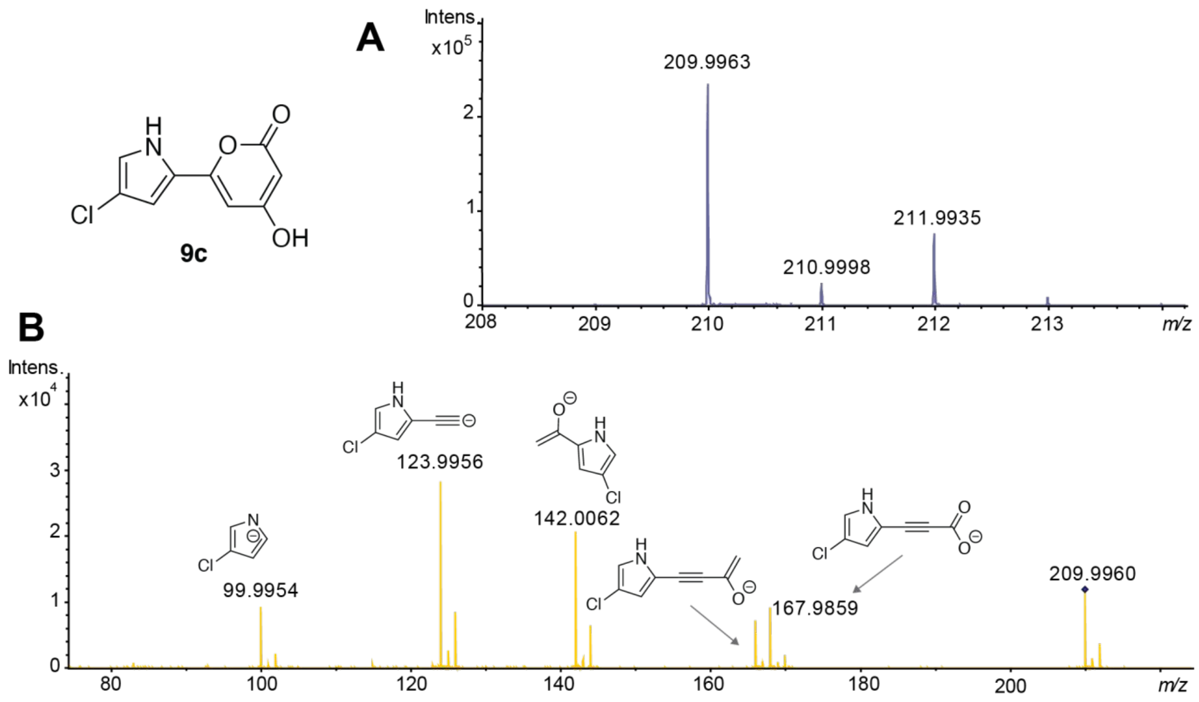

Fig. S45: MS[1] and MS[2] spectra of compound **9c**, product derived from substrate **8c**. (A) HRMS (ESI) identified molecule ions corresponding to $[M-H]^-$ for compound **9c** (*m/z* calculated for $C_9H_5ClNO_3$ 209.9963, found 209.9963). (B) MS[2] spectra of compound **9c** with rationalized structural annotations of fragment ions.

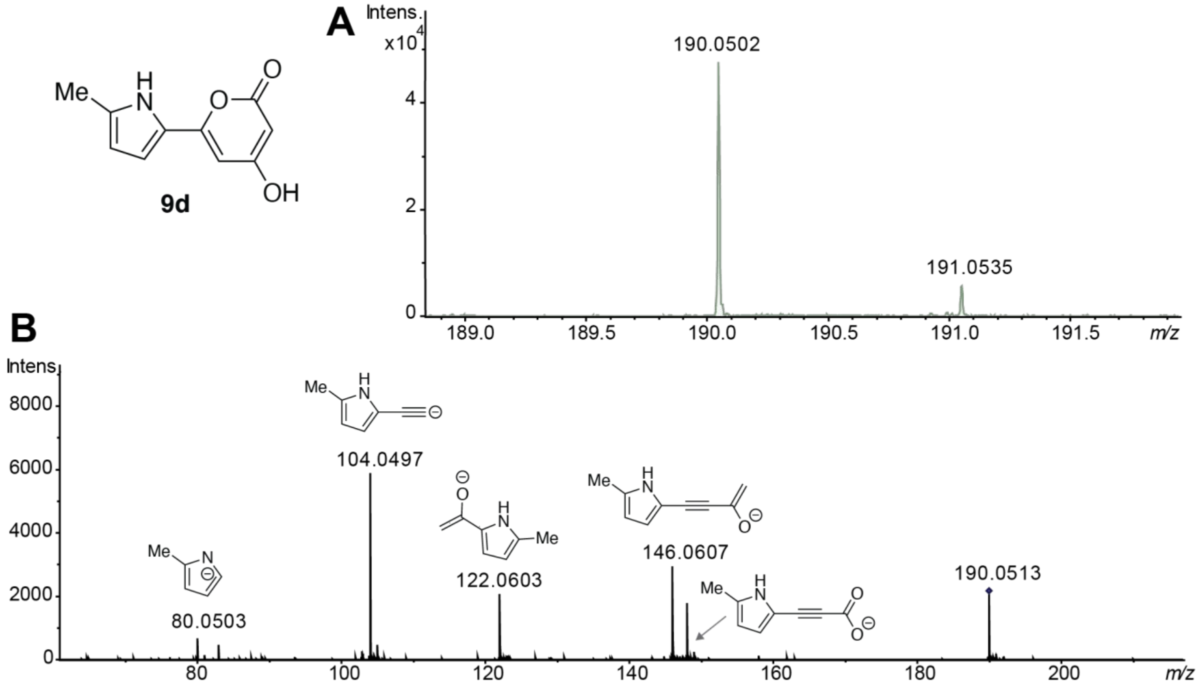

Fig. S46: MS^1 and MS^2 spectra of compound **9d**, product derived from substrate **8d**. (A) HRMS (ESI) identified molecule ions corresponding to $[M-H]^-$ for compound **9d** (*m/z* calculated for $C_{10}H_8NO_3$ 190.0510, found 190.0502). (B) MS^2 spectra of compound **9d** with rationalized structural annotations of fragment ions.

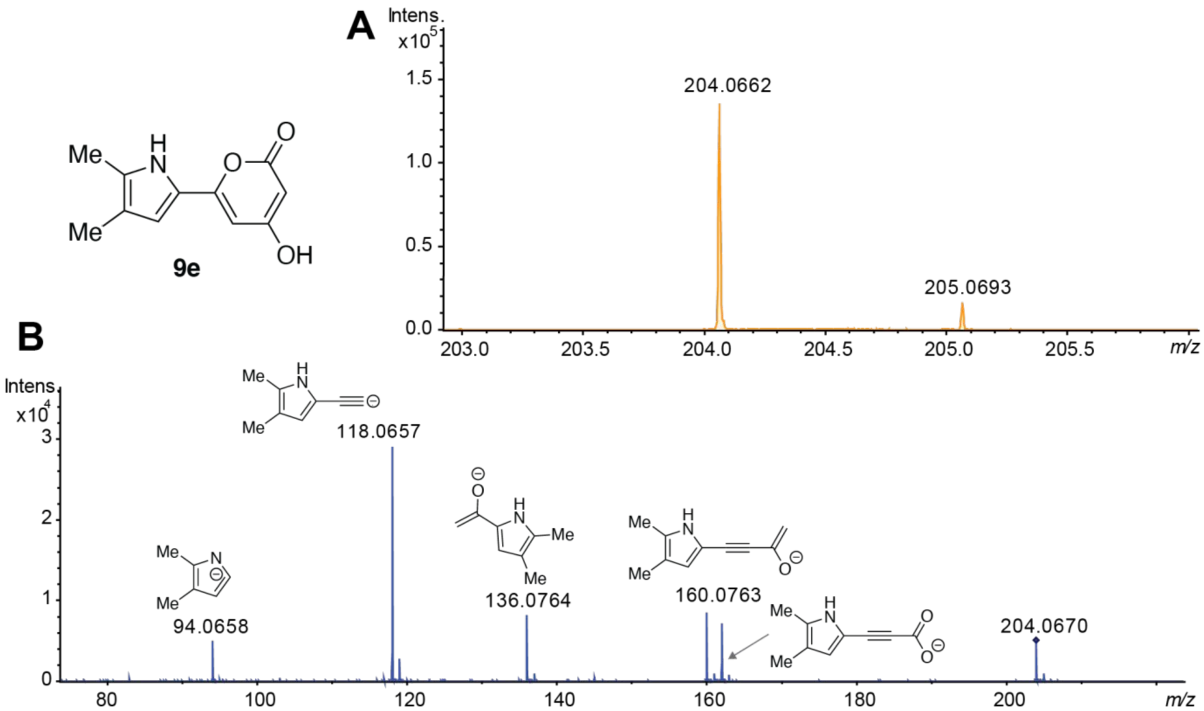

Fig. S47: MS^1 and MS^2 spectra of compound **9e**, product derived from substrate **8e**. (A) HRMS (ESI) identified molecule ions corresponding to $[M-H]^-$ for compound **9e** (*m/z* calculated for $C_{11}H_{10}NO_3$ 204.0666, found 204.0662). (B) MS^2 spectra of compound **9e** with rationalized structural annotations of fragment ions.

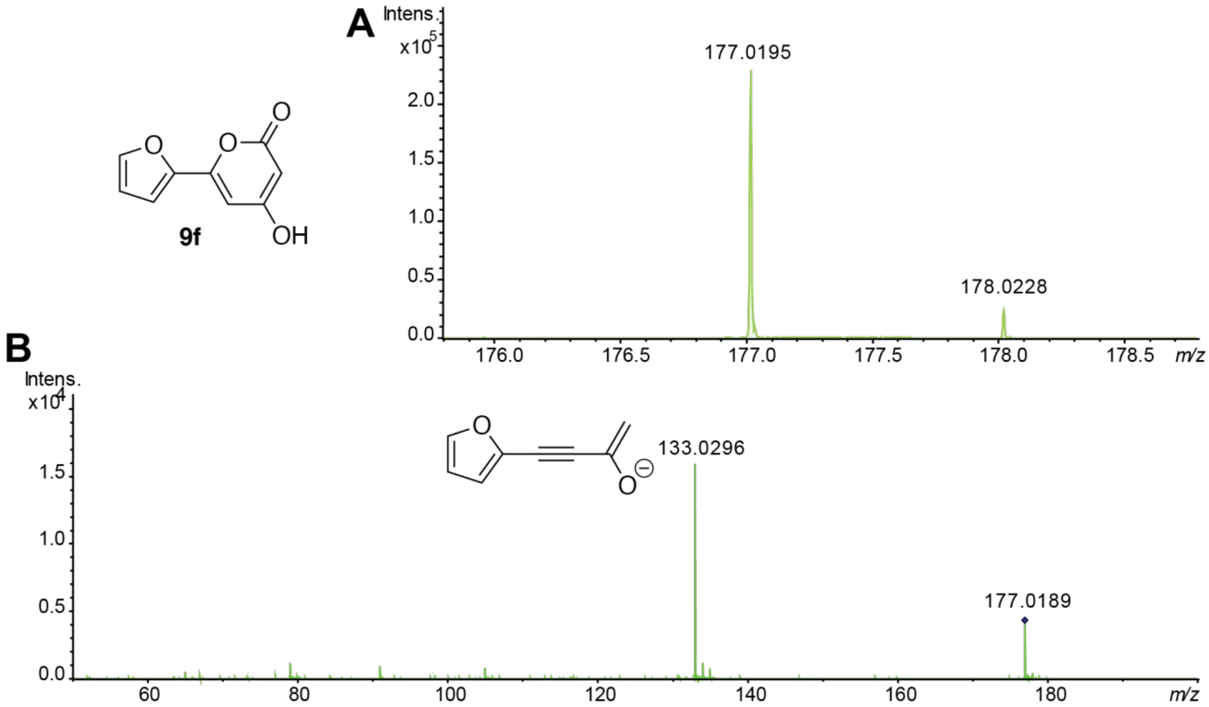

Fig. S48: MS[1] and MS[2] spectra of compound **9f**, product derived from substrate **8f**. (A) HRMS (ESI) identified molecule ions corresponding to $[M\text{-}H]^-$ for compound **9f** (*m/z* calculated for $C_9H_5O_4$ 177.0193, found 177.0195). (B) MS[2] spectra of compound **9f** with rationalized structural annotations of fragment ions.

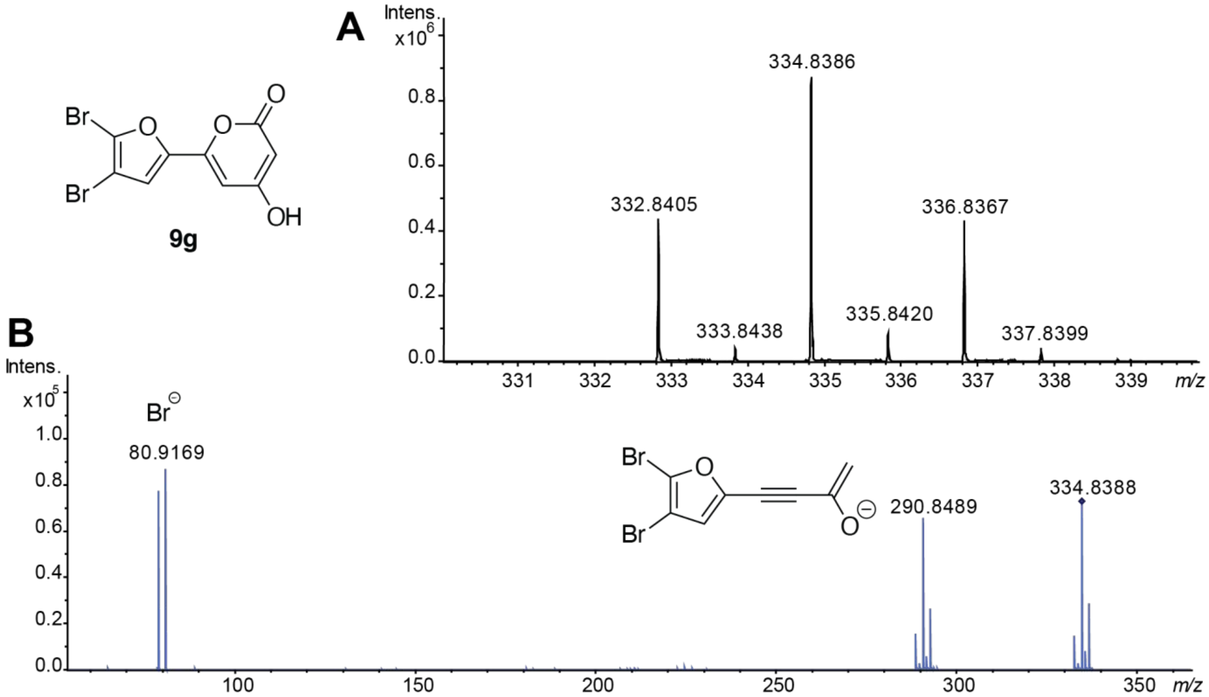

Fig. S49: MS[1] and MS[2] spectra of compound **9g**, product derived from substrate **8g**. (A) HRMS (ESI) identified molecule ions corresponding to $[M-H]^-$ for compound **9g** (*m/z* calculated for $C_9H_3Br_2O_4$ 332.8404, found 332.8405). (B) MS[2] spectra of compound **9g** with rationalized structural annotations of fragment ions.

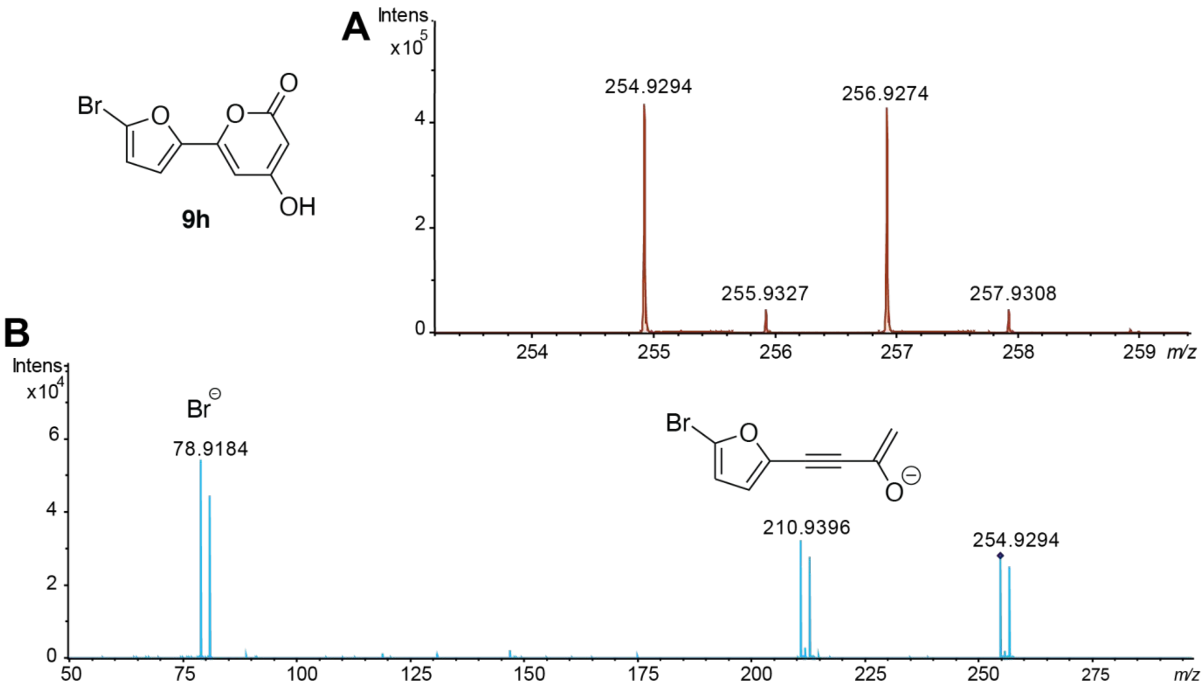

Fig. S50: MS[1] and MS[2] spectra of compound **9h**, product derived from substrate **8h**. (A) HRMS (ESI) identified molecule ions corresponding to $[M-H]^-$ for compound **9h** (*m/z* calculated for $C_9H_4BrO_4$ 254.9298, found 254.9294). (B) MS[2] spectra of compound **9h** with rationalized structural annotations of fragment ions.

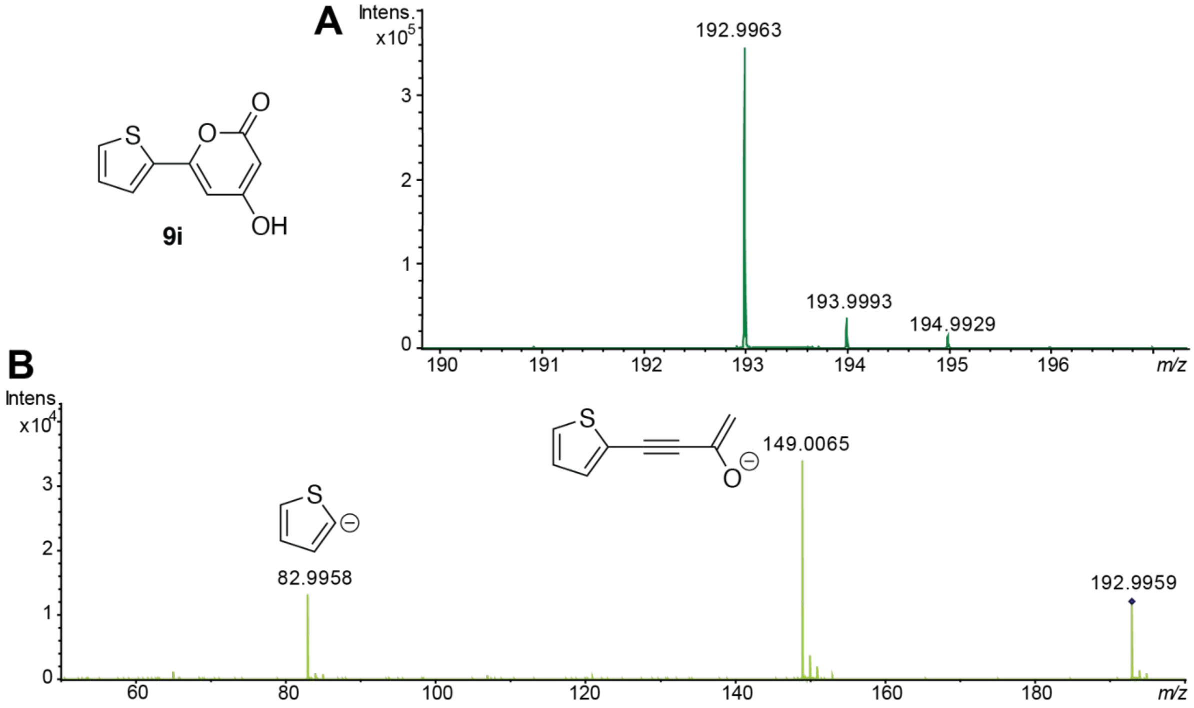

Fig. S51: MS[1] and MS[2] spectra of compound **9i**, product derived from substrate **8i**. (A) HRMS (ESI) identified molecule ions corresponding to [M-H]$^-$ for compound **9i** (*m/z* calculated for $C_9H_5O_3S$ 192.9965, found 192.9963). (B) MS[2] spectra of compound **9i** with rationalized structural annotations of fragment ions.

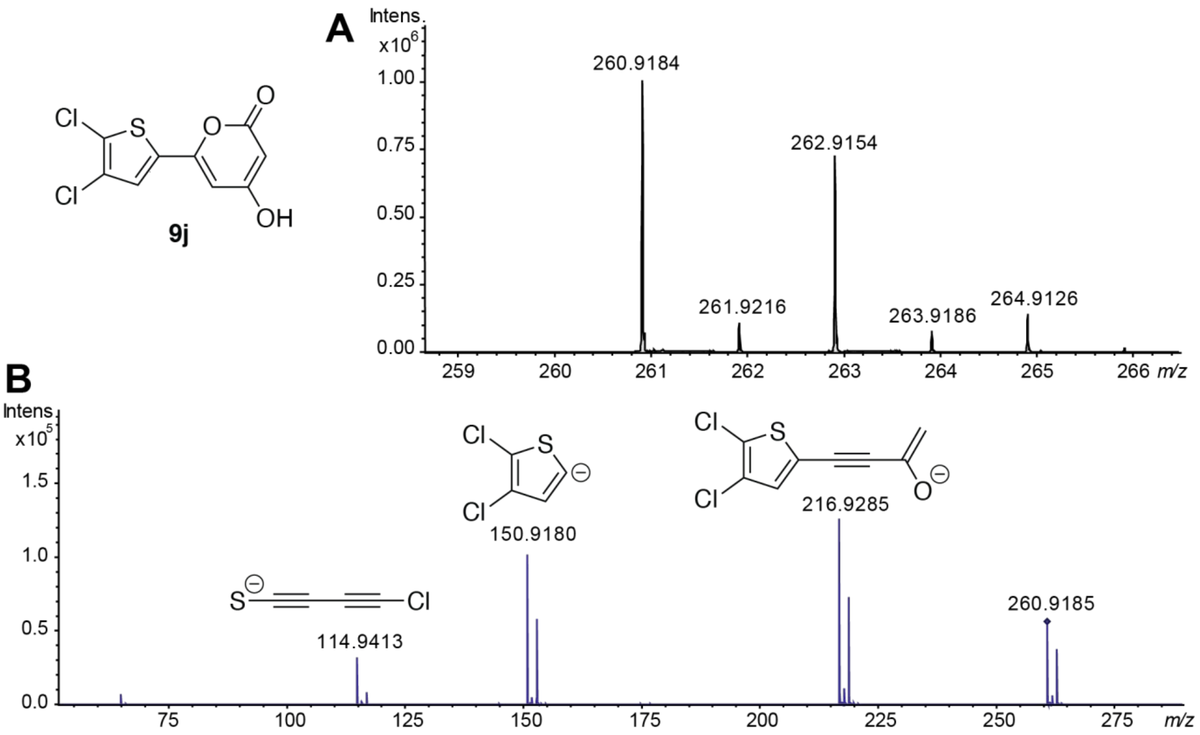

Fig. S52: MS[1] and MS[2] spectra of compound **9j**, product derived from substrate **8j**. (A) HRMS (ESI) identified molecule ions corresponding to $[M\text{-}H]^-$ for compound **9j** (*m/z* calculated for $C_9H_3Cl_2O_3S$ 260.9185, found 260.9184). (B) MS[2] spectra of compound **9j** with rationalized structural annotations of fragment ions.

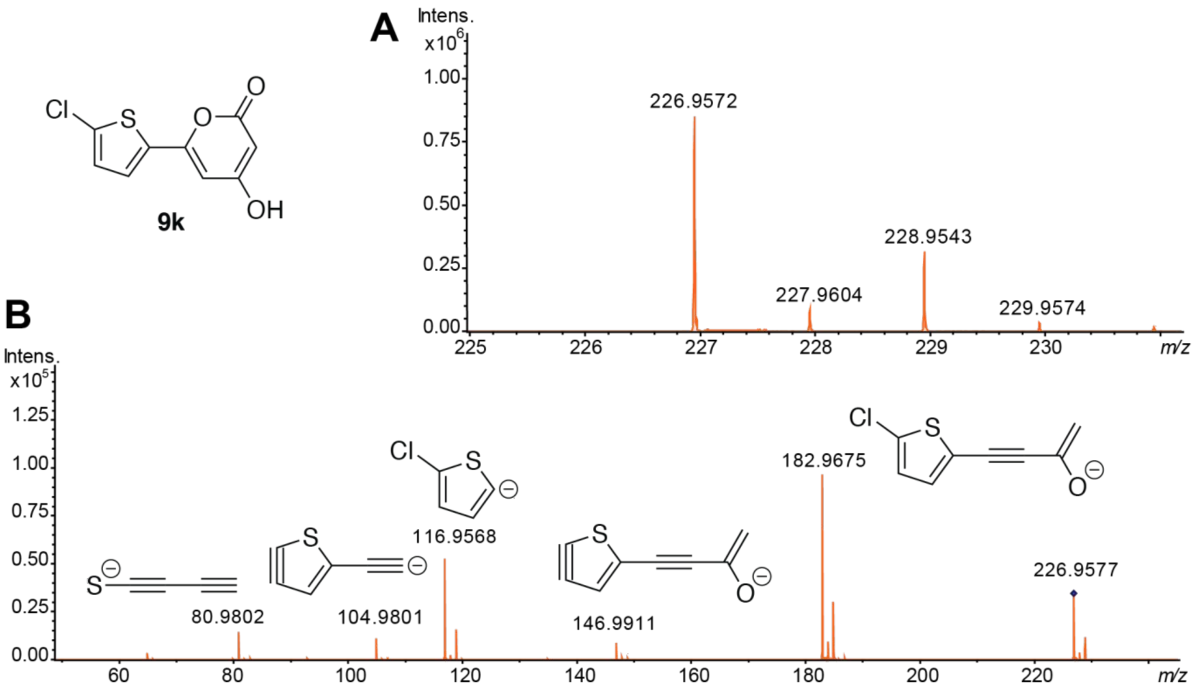

Fig. S53: MS[1] and MS[2] spectra of compound **9k**, product derived from substrate **8k**. (A) HRMS (ESI) identified molecule ions corresponding to [M-H]⁻ for compound **9k** (*m/z* calculated for $C_9H_4ClO_3S$ 226.9575, found 226.9572). (B) MS[2] spectra of compound **9k** with rationalized structural annotations of fragment ions.

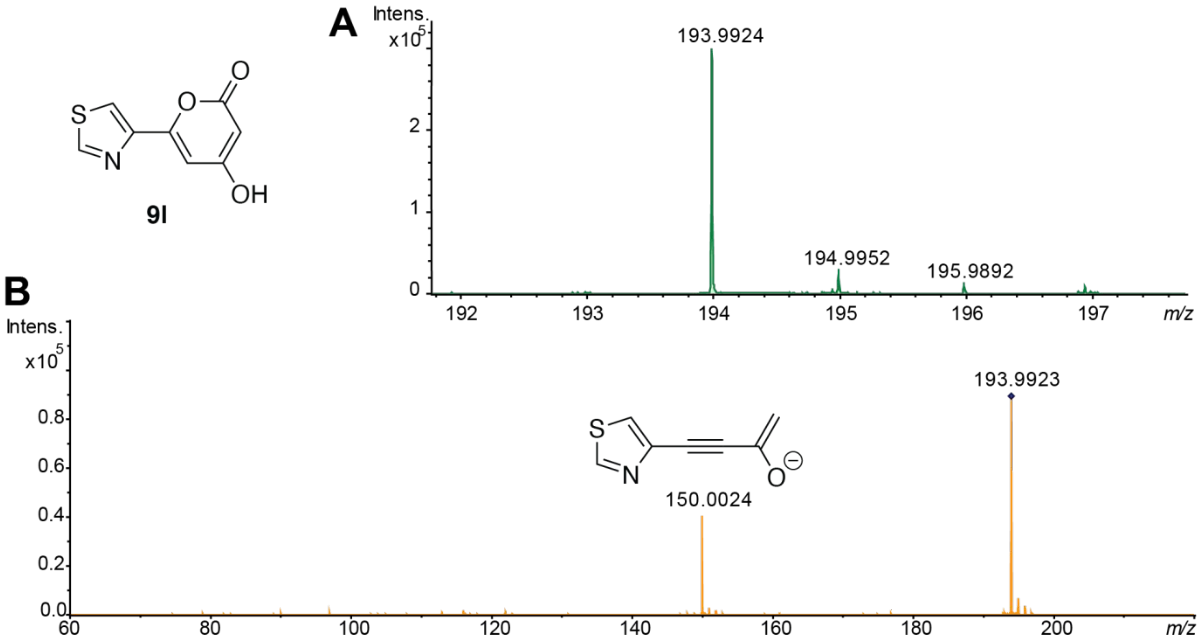

Fig. S54: MS[1] and MS[2] spectra of compound **9l**, product derived from substrate **8l**. (A) HRMS (ESI) identified molecule ions corresponding to [M-H]- for compound **9l** (*m/z* calculated for $C_8H_4NO_3S$ 193.9917, found 193.9924). (B) MS[2] spectra of compound **9l** with rationalized structural annotations of fragment ions.

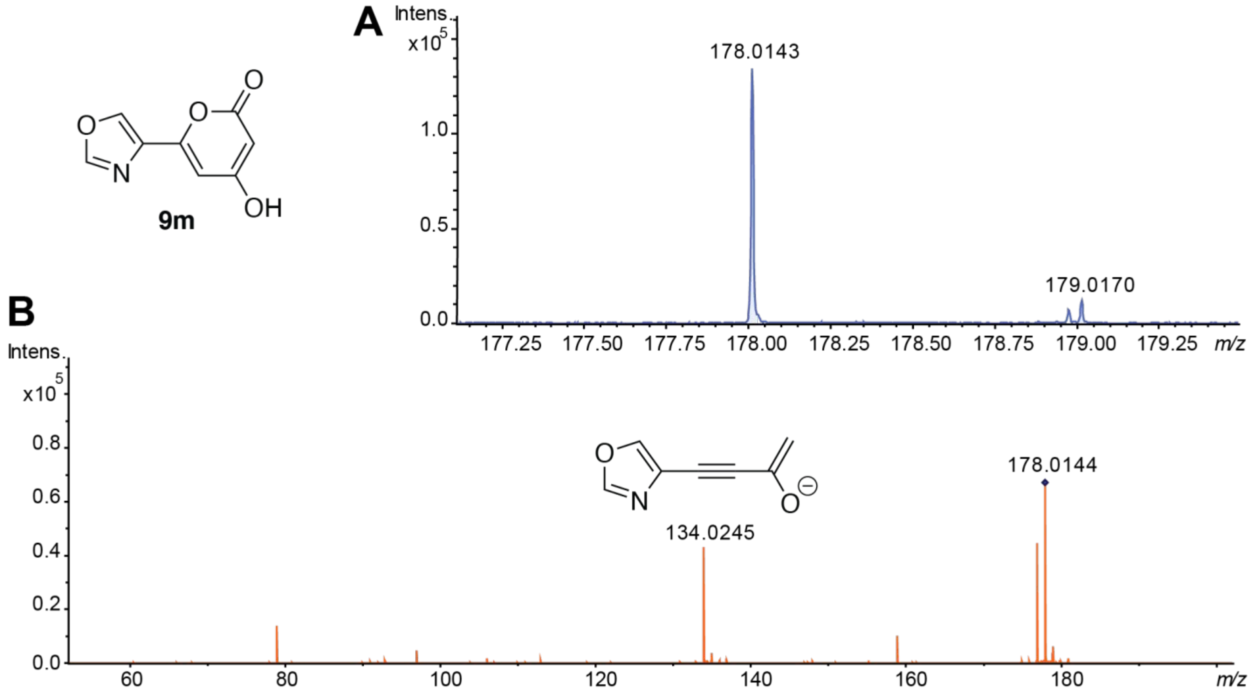

Fig. S55: MS[1] and MS[2] spectra of compound **9m**, product derived from substrate **8m**. (A) HRMS (ESI) identified molecule ions corresponding to $[M-H]^-$ for compound **9m** (*m/z* calculated for $C_8H_4NO_4$ 178.0146, found 178.0143). (B) MS[2] spectra of compound **9m** with rationalized structural annotations of fragment ions.

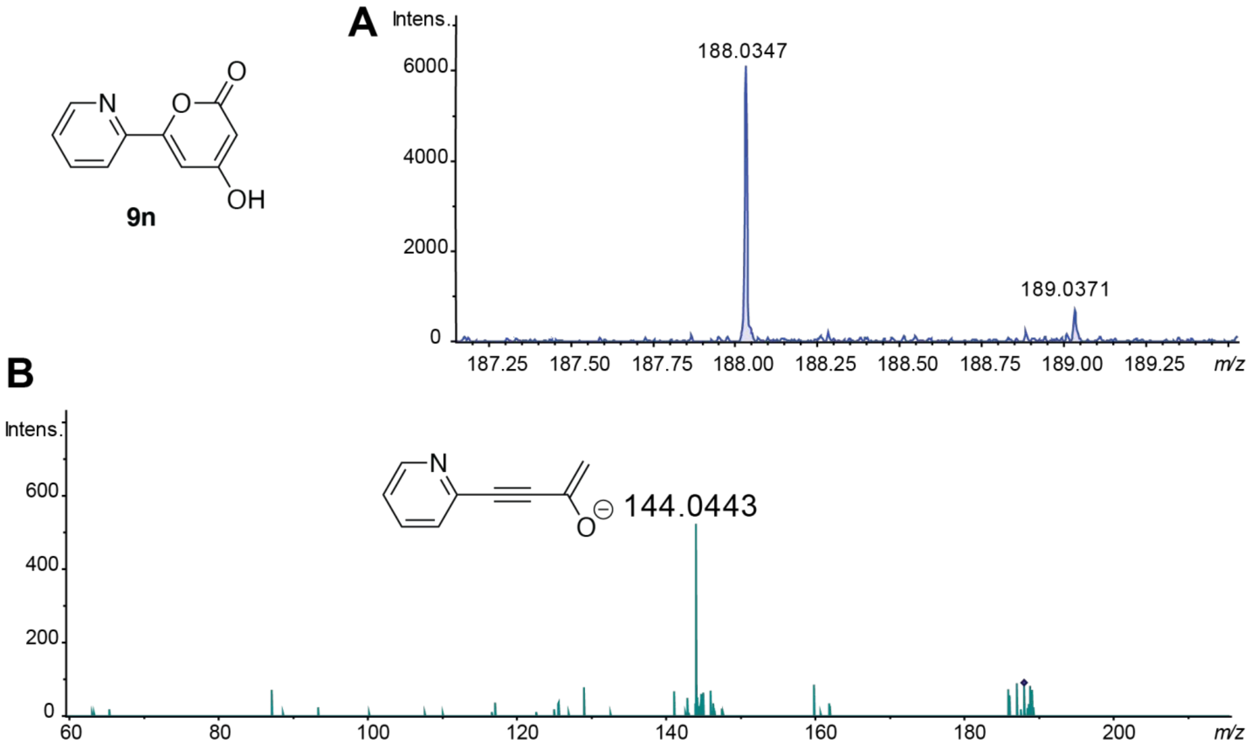

Fig. S56: MS[1] and MS[2] spectra of compound **9n**, product derived from substrate **8n**. (A) HRMS (ESI) identified molecule ions corresponding to [M-H]$^-$ for compound **9n** (*m/z* calculated for $C_{10}H_6NO_3$ 188.0353, found 188.0347). (B) MS[2] spectra of compound **9n** with rationalized structural annotations of fragment ions.

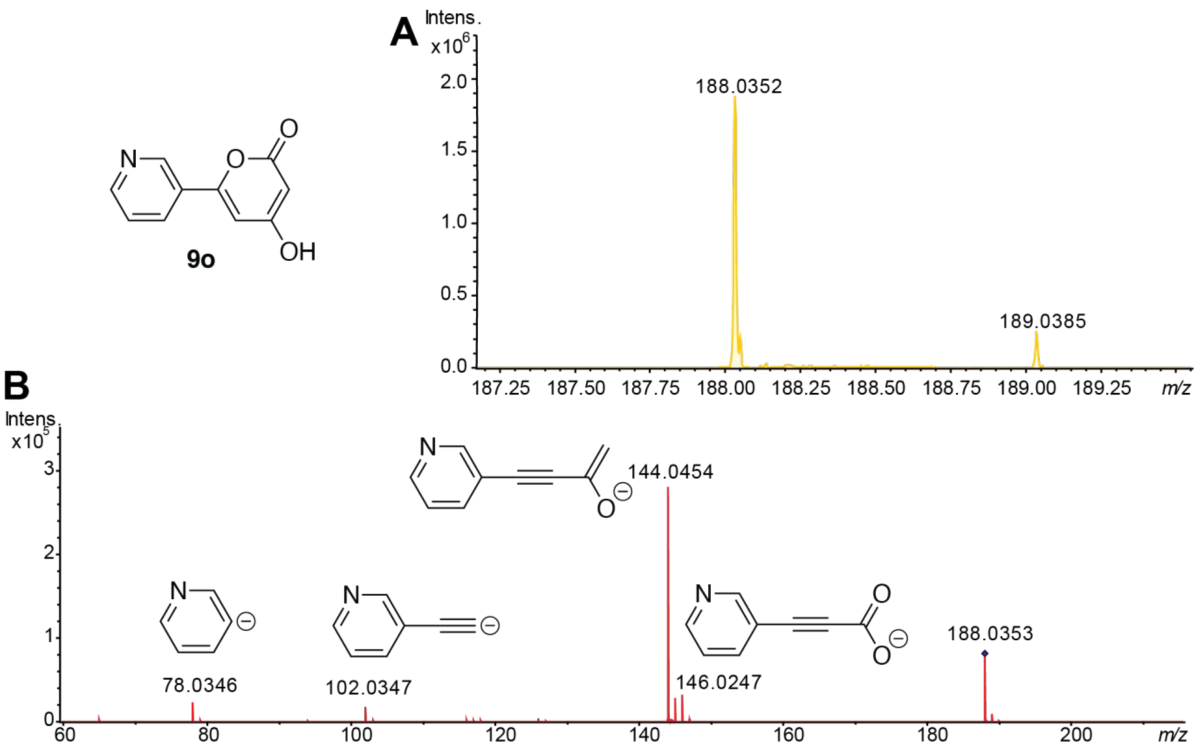

Fig. S57: MS[1] and MS[2] spectra of compound **9o**, product derived from substrate **8o**. (A) HRMS (ESI) identified molecule ions corresponding to [M-H]$^-$ for compound **9o** (*m/z* calculated for $C_{10}H_6NO_3$ 188.0353, found 188.0352). (B) MS[2] spectra of compound **9o** with rationalized structural annotations of fragment ions.

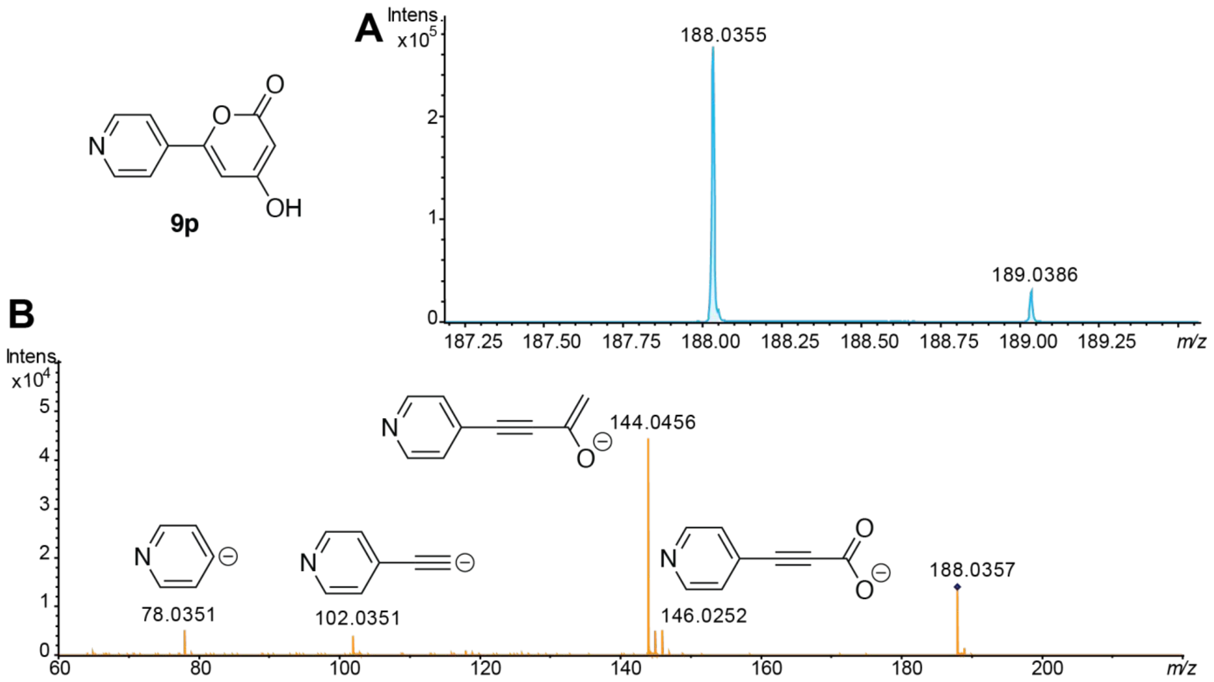

Fig. S58: MS[1] and MS[2] spectra of compound **9p**, product derived from substrate **8p**. (A) HRMS (ESI) identified molecule ions corresponding to $[M-H]^-$ for compound **9p** (*m/z* calculated for $C_{10}H_6NO_3$ 188.0353, found 188.0355). (B) MS[2] spectra of compound **9p** with rationalized structural annotations of fragment ions.

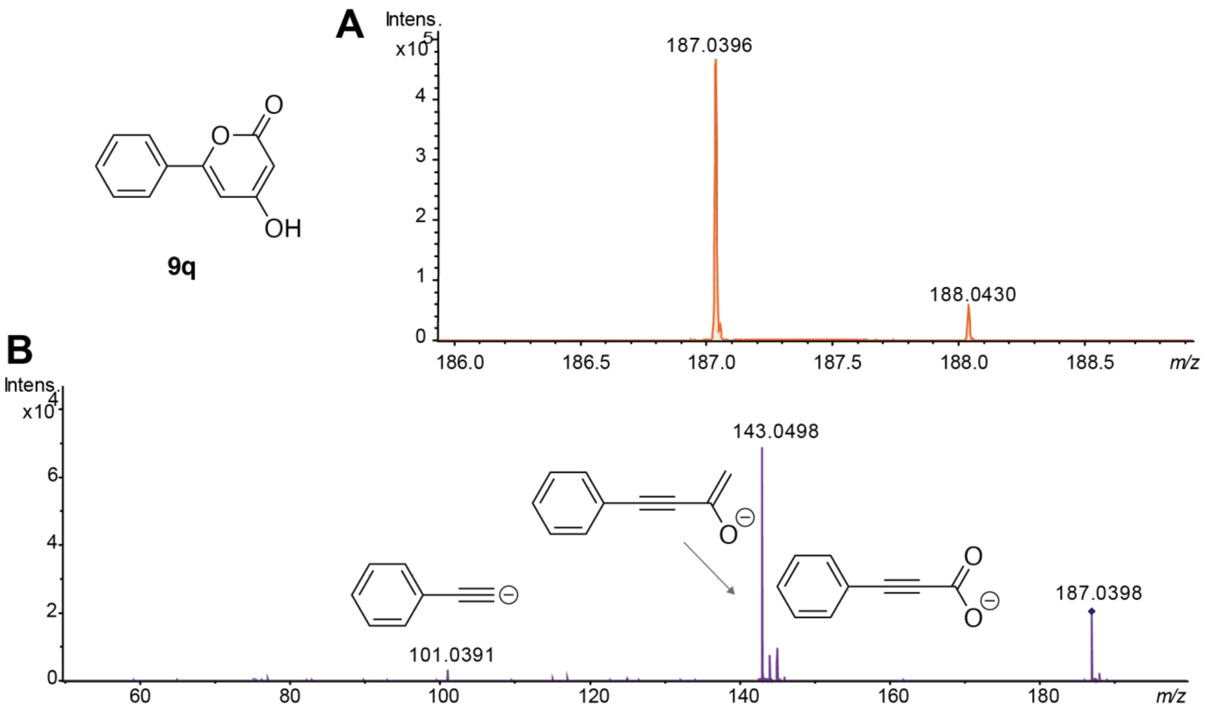

Fig. S59: MS[1] and MS[2] spectra of compound **9q**, product derived from substrate **8q**. (A) HRMS (ESI) identified molecule ions corresponding to $[M-H]^-$ for compound **9q** (*m/z* calculated for $C_{11}H_7O_3$ 187.0401, found 187.0396). (B) MS[2] spectra of compound **9q** with rationalized structural annotations of fragment ions.

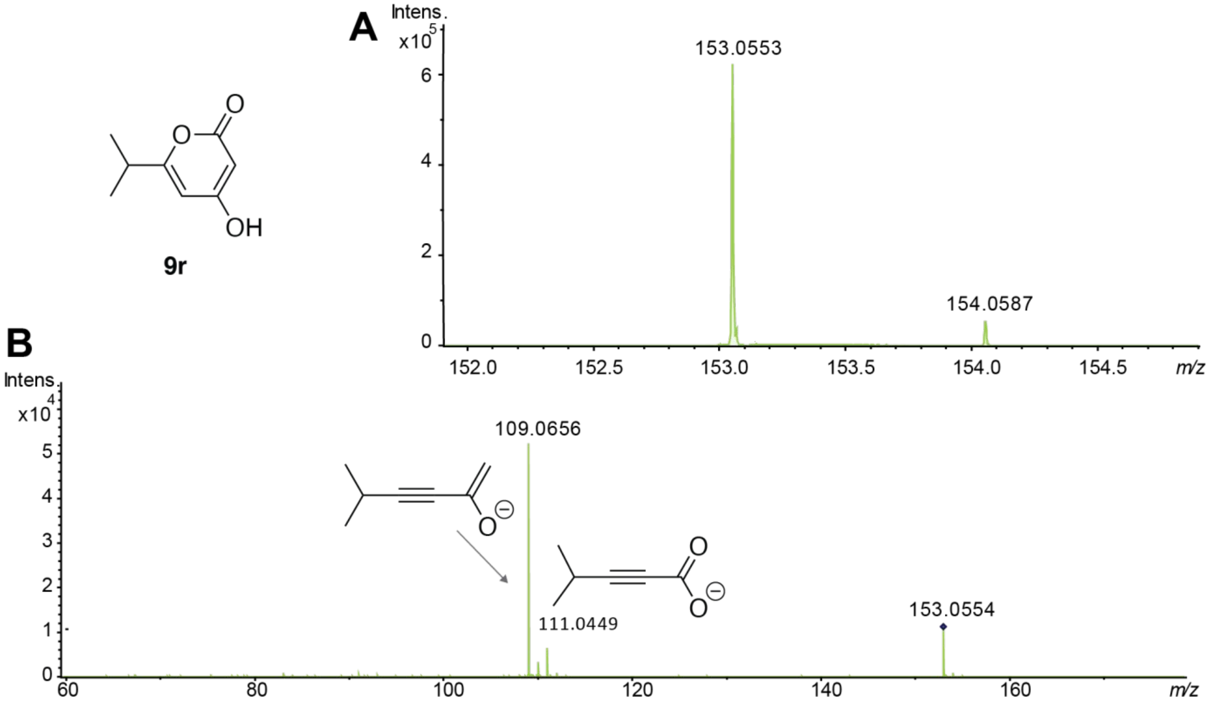

Fig. S60: MS^1 and MS^2 spectra of compound **9r**, product derived from substrate **8r**. (A) HRMS (ESI) identified molecule ions corresponding to $[M\text{-}H]^-$ for compound **9r** (*m/z* calculated for $C_8H_9O_3$ 153.0557, found 153.0553). (B) MS^2 spectra of compound **9r** with rationalized structural annotations of fragment ions.

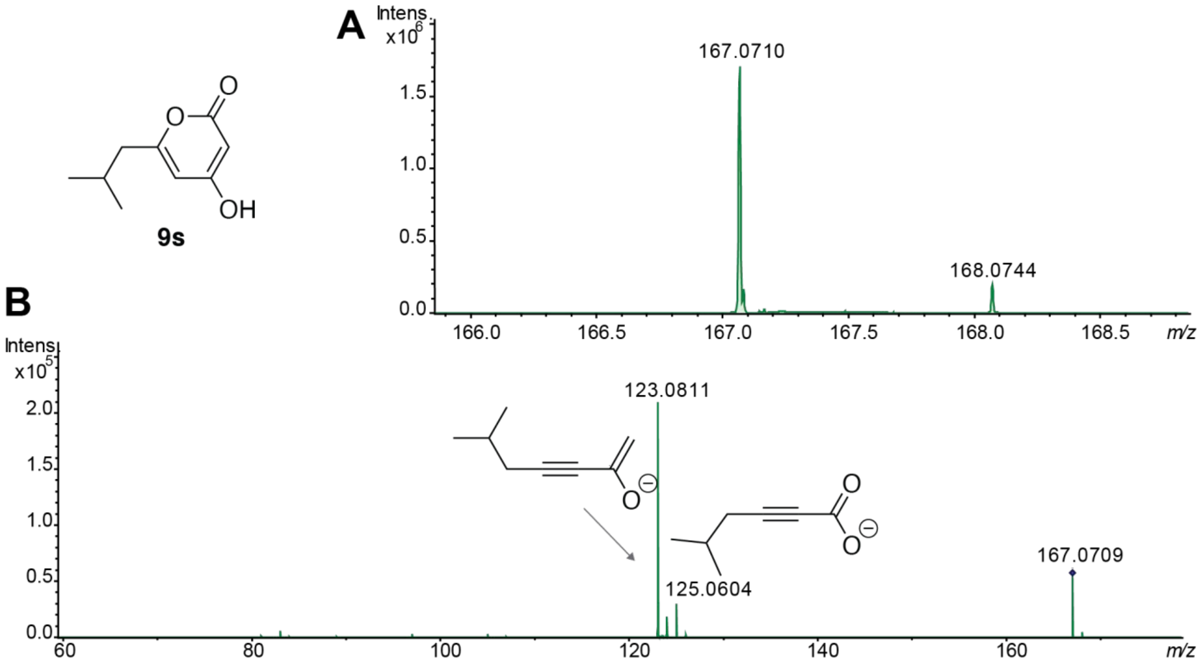

Fig. S61: MS^1 and MS^2 spectra of compound **9s**, product derived from substrate **8s**. (A) HRMS (ESI) identified molecule ions corresponding to $[M\text{-}H]^-$ for compound **9s** (*m/z* calculated for $C_9H_{11}O_3$ 167.0714, found 167.0710). (B) MS^2 spectra of compound **9s** with rationalized structural annotations of fragment ions.

SUPPLEMENTARY REFERENCES

1. Yi, D.; Acharya, A.; Gumbart, J. C.; Gutekunst, W. R.; Agarwal, V., Gatekeeping ketosynthases dictate initiation of assembly line biosynthesis of pyrrolic polyketides. *J. Am. Chem. Soc.* **2021,** *143* (20), 7617-7622.

2. Yi, D.; Niroula, D.; Gutekunst, W. R.; Loper, J. E.; Yan, Q.; Agarwal, V., A nonfunctional halogenase masquerades as an aromatizing dehydratase in biosynthesis of pyrrolic polyketides by type I polyketide synthases. *ACS Chem. Biol.* **2022,** *17* (6), 1351-1356.

3. Agarwal, V.; Diethelm, S.; Ray, L.; Garg, N.; Awakawa, T.; Dorrestein, P. C.; Moore, B. S., Chemoenzymatic synthesis of acyl coenzyme A substrates enables *in situ* labeling of small molecules and proteins. *Org. Lett.* **2015,** *17* (18), 4452-4455.

4. Wang, M. Z.; Xu, H.; Liu, T. W.; Feng, Q.; Yu, S. J.; Wang, S. H.; Li, Z. M., Design, synthesis and antifungal activities of novel pyrrole alkaloid analogs. *Eur. J. Med. Chem.* **2011,** *46* (5), 1463-72.

5. Meiser, P.; Weissman, K. J.; Bode, H. B.; Krug, D.; Dickschat, J. S.; Sandmann, A.; Müller, R., DKxanthene biosynthesis—understanding the basis for diversity-oriented synthesis in myxobacterial secondary metabolism. *Chem. Biol.* **2008,** *15* (8), 771-781.

6. Thiede, S.; Wosniok, P. R.; Herkommer, D.; Debnar, T.; Tian, M.; Wang, T.; Schrempp, M.; Menche, D., Total synthesis of leupyrrins A1 and B1, highly potent antifungal agents from the myxobacterium *Sorangium cellulosum*. *Chem. Eur. J* **2017,** *23* (14), 3300-3320.

7. Kusebauch, B.; Brendel, N.; Kirchner, H.; Dahse, H.-M.; Hertweck, C., Assessing oxazole bioisosteres as mutasynthons on the rhizoxin assembly line. *Chembiochem* **2011,** *12* (15), 2284-2288.
